# Innovative socio-sanitary rehabilitation models in central nervous system disorders: a systematic review

**DOI:** 10.3389/fpubh.2026.1742821

**Published:** 2026-02-13

**Authors:** G. Morlino, M. Chimienti, O. Zerellari, R. Shkreli, D. Ndreca, K. Stefanidhi, S. Gjergji, F. Leonforte, V. Nicosia, A. Mistretta, E. Buonomo, G. Liotta, L. Palombi

**Affiliations:** 1 Catholic University Our Lady of Good Counsel, Tirana, Albania; 2 Department of Biomedicine and Prevention, University of Rome Tor Vergata, Rome, Italy; 3 Department of Integrated Hygiene, Organizational and Service Activities (Structural Department), Health Management, University Hospital Polyclinic “G. Rodolico - San Marco”, Catania, Italy; 4 Department of Medical and Surgical Sciences and Advanced Technologies “G.F Ingrassia”, University of Catania, Catania, Italy

**Keywords:** central nervous system, integrated care, neurorehabiliation, rehabiliatation, socio-sanitary

## Abstract

**Introduction:**

Because neurological disorders profoundly affect patients’ lives, care models are increasingly oriented toward an integrated clinical and socio-sanitary approach. This being said, the actual context of this integration and the literature itself presents notable gaps and inconsistencies. The aim of this study was to review the existing literature to provide an overview of the current implementation of these services, while identifying gaps and potential areas for improvement.

**Methodology:**

We conducted a systematic review following the PRISMA guidelines including only peer-reviewed articles retrieved from PubMed, Scopus, and Web of Science that focused on neurological conditions and socio-sanitary services. For each peer-reviewed study, we identified specific characteristics to review them.

**Results:**

Sixty-four studies were included, with stroke as the most frequently studied condition, followed by Alzheimer’s disease, multiple sclerosis, Parkinson’s disease, and spinal cord injury. Most of the services were embedded within public health systems with a prevalence of home or territorial-setting.

**Conclusion:**

The review identifies promising evidence of positive impacts associated with socio-sanitary services, particularly in relation to functional outcomes, patient satisfaction and support for community living. At the same time there are important gaps in their implementation, integration, and the methodological consistency of existing studies. Across multiple settings, continuity of care appears fragmented, and case management functions are inconsistently implemented or, in some cases, entirely absent. Together, these findings suggest that while socio-sanitary models can offer meaningful benefits, persistent gaps in coordination and follow-up represent key structural barriers to achieving effective and sustainable integration.

## Introduction

In 2021, neurological disorders affected around 3.4 billion people globally, accounting for 443 million disability-adjusted life years (DALYs) and also causing over 11 million deaths, making them the leading cause of global disease burden (1).

Important elements of this burden include aphasia, that can lead to social isolation, as well as motor deficits, that can lead to limitations of autonomy. As a result, during the course of the disease, patients encounter a deterioration of their health ranging from depression to poor self-maintenance (2).

One important and growing aspect of the rehabilitation process of these patients is socio-sanitary support. In this article, socio-sanitary services are defined as integrated models of care that combine health, rehabilitation, and social support interventions, consistent with WHO frameworks on integrated people-centered care, long-term care, and rehabilitation (3–5). Numerous studies support the integration of social support in achieving the final goal which is to guarantee a good standard of living for these patients by addressing not only physical health but also the psychosocial part of the illness, particularly in neurological pathologies with a high impact on both these dimensions (6, 7). This is particularly relevant for disorders which are associated with long-term disability. The most mentioned long-term disability CNS diseases, and that as a consequence, emerged from our literature search are stroke, dementia, multiple sclerosis, Parkinson’s disease, traumatic brain injury, and spinal cord injury. These conditions exemplify clinical situations where social vulnerability is a vital concern and on the other hand socio-sanitary integration is crucial for continuity of care, case management, and community reintegration.

Nonetheless despite growing recognition of integrated care, as a component of the treatment of neurological disease, the literature on social and sanitary services available for these patients remains fragmented and lacks a unified framework. Moreover the primary studies themselves revealed scientific limitations but also gaps of the services that hinder their implementation. Therefore, the objectives of this systematic review are:

1. To review and synthesize the existing models of socio-sanitary service for individuals with neurological disease, including their mode of conduct, placement and type of service.
2. To identify key limitations, barriers and gaps in service implementation.
3. To identify limitations of the present literature that render its conclusions provisional.

These insights could be helpful in the future for creating inclusive and long term public health policies for neurological conditions, that address both clinical and psychosocial needs of the patients.

## Methodology

To maintain consistency and transparency in the selection process, the inclusion and exclusion criteria were defined prior to screening, in accordance with the objectives of this review using the PICOS framework. These criteria were developed with reference to the **PRISMA** (8) (Preferred Reporting Items for Systematic Reviews and Meta-Analyses) guidelines, which support methodological clarity in systematic review design (Table 1).

**Table 1:** PICOS.

PICOS	Inclusion Criteria	Exclusion Criteria
(P)Population	Studies including individuals diagnosed with one or more neurological conditions, including: Stroke Multiple Sclerosis Parkinson’s disease Dementia Traumatic Brain Injury (TBI) Neuromuscular disorders (e.g., ALS, muscular dystrophies)	Studies not involving individuals with neurological conditions. Studies limited to a single age group (only children or only older adults) unless age focus is clearly relevant to the neurological condition. Studies focusing on non-neurological conditions (e.g., diabetes, cancer, psychiatric illnesses) without a meaningful neurological overlap.
(I)Intervention/concept	Studies addressing the planning, delivery, or evaluation of social and/or socio-sanitary services, including: Community-based rehabilitation programs Neurological rehabilitation services Programs for social integration or functional rehabilitation Support for living conditions (e.g., home care, residential care) Psychosocial or social support interventions Services aiming to improve quality of life, access to care, or equity	Studies focusing solely on medical or surgical interventions (pharmacological treatments, neurosurgical procedures) without any socio-sanitary or social service component. Interventions unrelated to socio-sanitary services (e.g., use of assistive technologies or robotic devices) when not integrated into broader care services.
(C)Comparison	No specific comparator required	No comparator required (not a basis for exclusion)
(O)Outcomes	Studies evaluating outcomes related to: Quality of life Access to care and equity Functional improvement Impact or effectiveness of socio-sanitary services	Studies that do not evaluate outcomes related to socio-sanitary service impact (e.g., studies with purely clinical or biomedical outcomes).
(S)Study designs	Quantitative studies (e.g., randomized controlled trials, cohort or case–control studies) Qualitative studies (e.g., interviews, focus groups) Studies from any geographical region or care setting (hospital, outpatient, community, residential facilities) Articles published in English or Italian	Editorials, opinion papers, commentaries, and non–research articles. Studies without full-text availability. Articles published in languages other than English or Italian.

A comprehensive literature search was conducted across three international databases: PubMed, Scopus and Web of Science for studies published from 2013 to 2024. This time frame was selected to capture the most recent decade of reforms in integrated and socio-sanitary care, coinciding with global policy shifts promoted by WHO, including the *Rehabilitation 2030* initiative and the *Integrated Care for Older People* framework, which emphasize people-centered, coordinated, and integrated service models (3–5).

The search terms were grouped into four main conceptual domains, with the goal being to capture a broad spectrum of literature relating the neurological conditions and socio-sanitary services.

The first domain focused on different neurological pathologies, such as all types of *dementia*, *cerebrovascular disorders, Parkinson, multiple sclerosis, ALS, spinal cord injury.*

The second domain addressed social and environmental conditions, with concepts: *community resource, social condition, social exclusion, vulnerability and socioeconomic factors.*

The third domain targeted different types of socio-sanitary services in the long term rehabilitation of neurological disorders, such as: *neurological rehabilitation, early rehabilitation, continuity of care, nursing care, home-based care and day care.*

Lastly, the fourth domain included exclusion criteria with the scope to eliminate irrelevant studies such as: c*ardiovascular disease, diabetes, cancer, psychiatric disorders, children, robotics, surgery, Covid-19, advanced technologies.*

For a more logical search the Boolean words **AND, OR, NOT** were used. The complete search strings for each database are provided in the Supplementary File 1.

### Risk of bias assessment

For the assessment of bias risk, the JBI (Joanna Briggs Institute) tool (9) and the RTI Item database (10) were used for experimental and observational studies, respectively.

In particular a modified version of these tools was used giving more weight to aspects that matter most for the validity of the results. We did not penalise studies for not blinding participants or providers, since this is often unrealistic in non-pharmacological and socio-sanitary interventions. These limitations were acknowledged, but they were not enough on their own to reduce the overall quality rating of a study. On the other hand we focused mainly on elements such as how the randomisation was done, whether allocation was concealed, if assessors were blinded, how drop-outs were managed, and whether analyses followed intention-to-treat principles. This weighted approach was implemented through an increase of the cut-off from for overall risk-of-bias classification from four to five risk-increasing responses, as preliminary testing showed that the lower threshold disproportionately penalised studies due to structurally unavoidable design features. In particular, articles with 0–5 risk-increasing responses were rated as low risk of bias, 6–7 moderate risk and 8 or more high risk.

The complete risk of bias assessment tables are available in the Supplementary File 1.

Two researchers conducted a double-blind assessment of the selected articles. In the event of conflicting results, a third expert reviewer resolved the conflict by providing their own assessment.

After the risk of bias assessment for each included article, data were systematically extracted using a predefined framework. Two complementary data extraction tables were developed. The first table summarizes core study characteristics, including bibliographic information, study design, neurological condition, service setting, intervention components, and targeted outcomes. The second table focuses on implementation-related aspects, reported facilitators and barriers, and contextual characteristics. Both tables are provided in the Supplementary File 1.

## Results

### Articles selection

Initially, a total of 1,727 articles were identified through three databases: **PubMed** (1,219 articles), **Scopus** (366 articles) and **Web of Science** (142 articles). Before screening, 235 duplicate articles were removed, leaving 1,492 articles to be screened. Both abstract and full-text screenings according to the predefined PICOS criteria were performed independently by two reviewers in a double-blind manner. Disagreements between reviewers were resolved by a third senior reviewer with greater methodological expertise. In the first screening phase, only the abstracts of the 1,492 articles were reviewed. As a result 1,270 articles were excluded because they did not meet the initially established PICOS criteria. More specifically because they focused on non-neurological conditions, investigated acute or highly specialized clinical interventions unrelated to socio-sanitary integration, lacked a clear social or community-based component, or did not evaluate service models or pathways. The remaining 222 articles were then subject to a second, full-text screening phase. During the full-text screening, the most frequent reasons for exclusion were the absence of a socio-sanitary or social service component despite initial relevance, exclusive focus on biomedical or pharmacological outcomes, lack of reported results, and language restrictions. As a result, 158 articles were excluded and in particular 5 articles were written in a language other than Italian or English, and 147 articles did not meet the PICOS criteria upon detailed examination. A total of 64 studies met the inclusion criteria and were incorporated into the final review.

### Quality assessment

**Figure 1:**
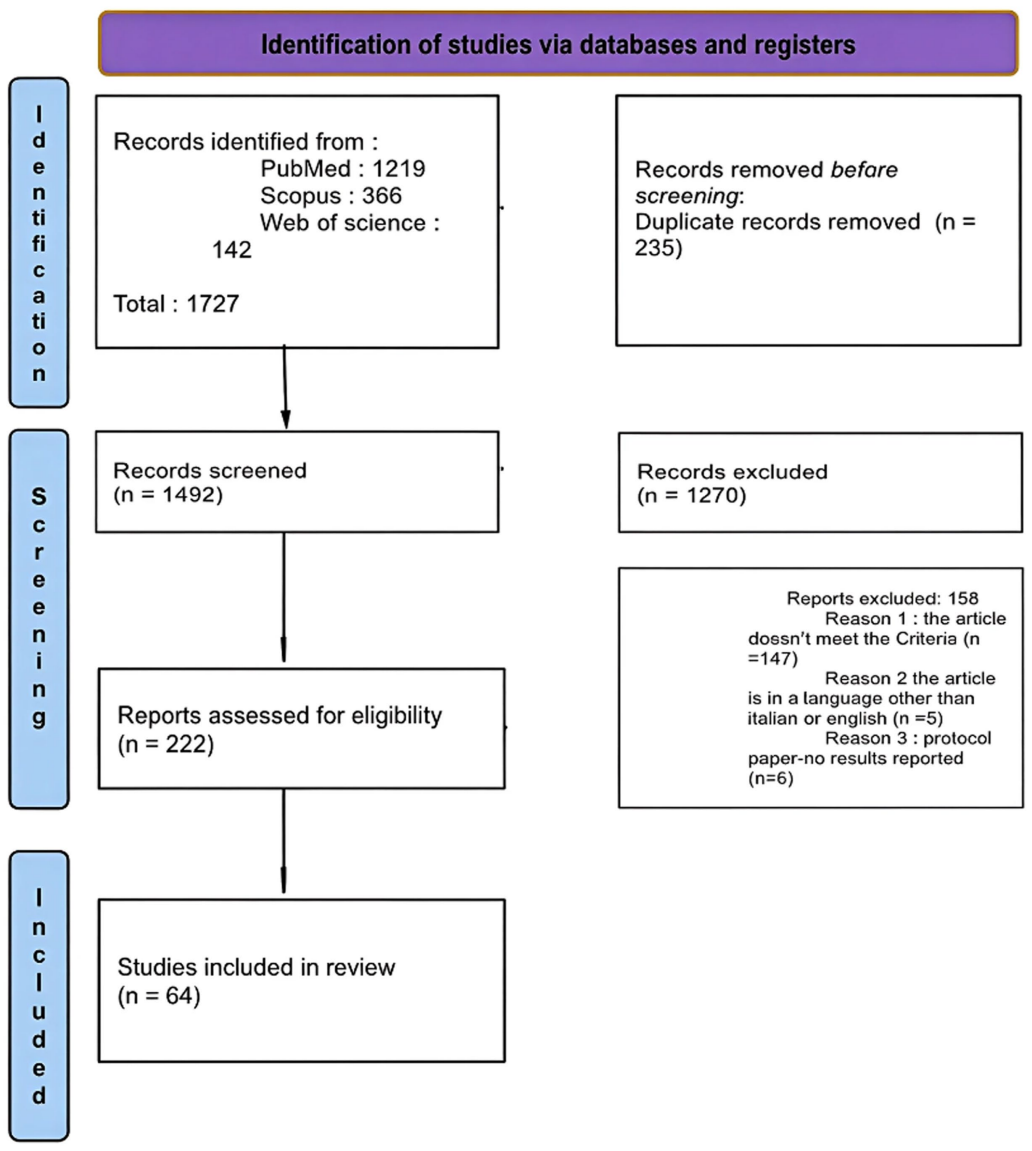
Flow diagram.

**Figure 2:**
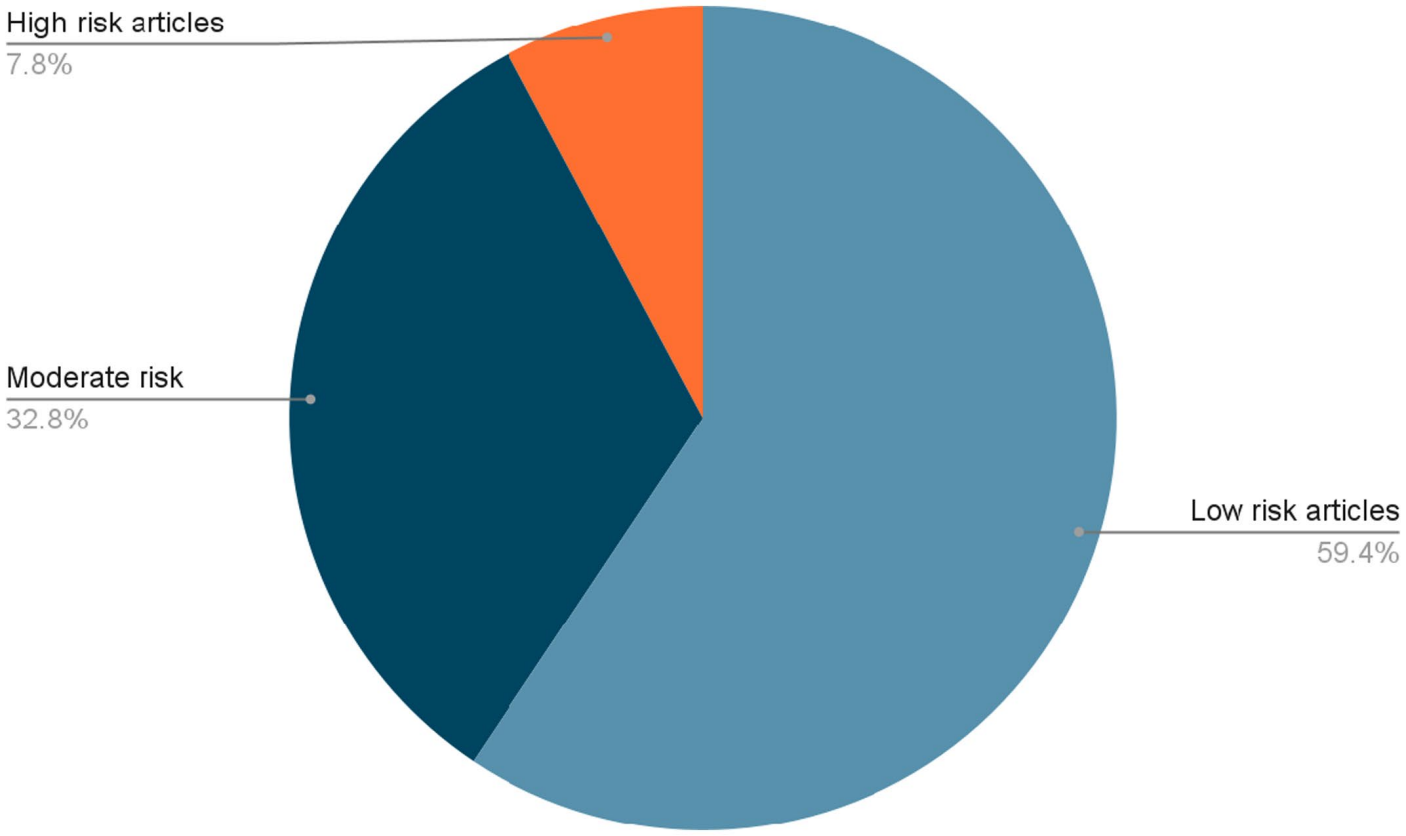
The percentage of articles for each level or studies.

The risk of bias assessment resulted in a total of 5 articles with high risk of bias, 21 with a moderate risk of bias and 38 with a low risk of bias. Figure 1 shows the percentage of studies for each risk level. Figure 2 shows the percentage of articles at each risk level for each disease.

While a considerable proportion of the included studies were assessed as having a moderate risk of bias, this classification does not constitute a comprehensive reflection of their overall quality and does not compromise the validity or quality of this study.

**Figure 3:**
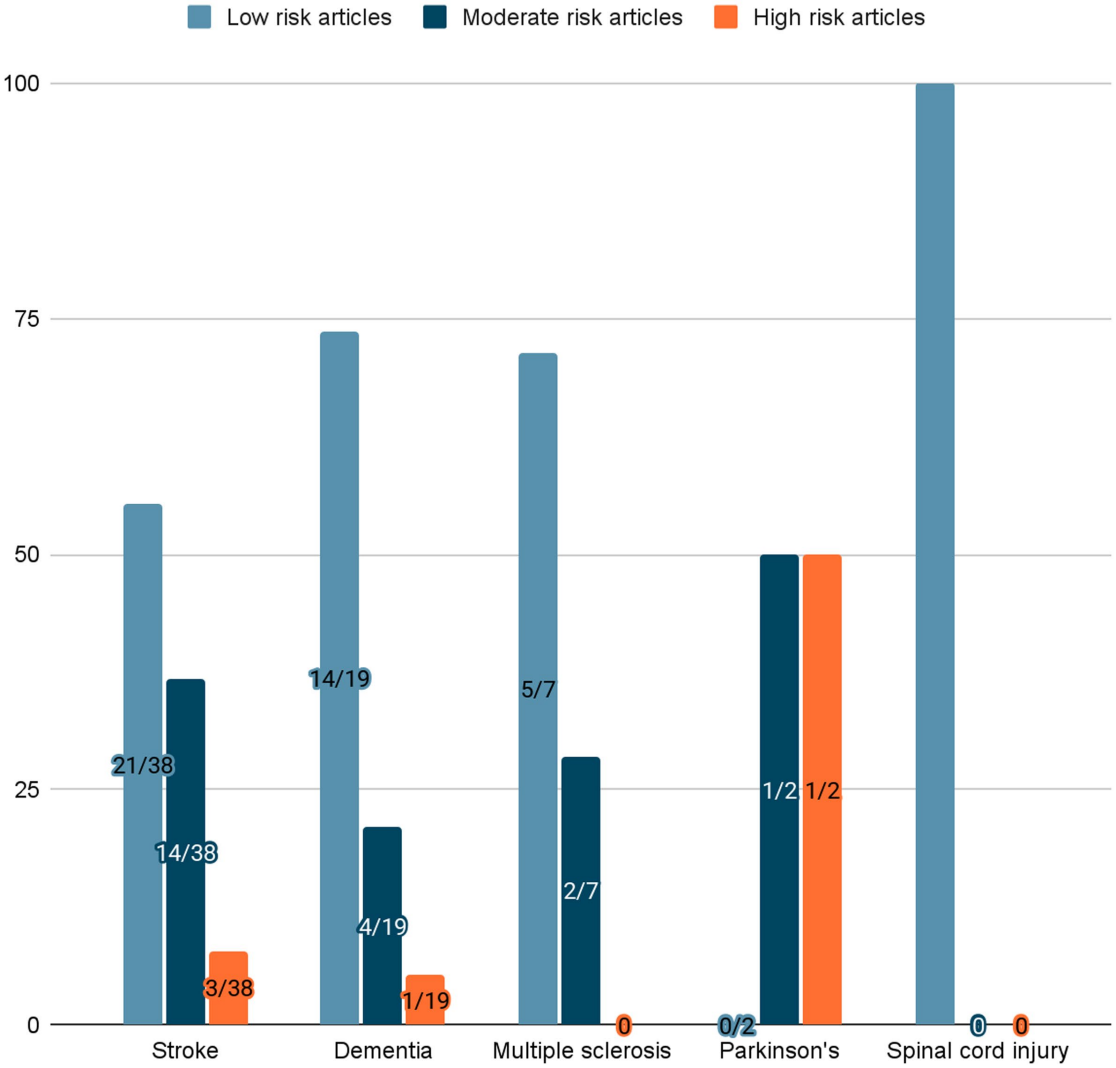
Percentage of articles at each risk level for each disease.

Given the methodological heterogeneity of included studies, risk-of-bias ratings were used to contextualize findings rather than to establish direct comparability or hierarchical ranking across study designs (Figure 3).

### Narrative synthesis

The review brought together studies examining a diverse set of sociosanitary interventions for individuals with central nervous system (CNS) conditions. These were grouped according to the type of service and neurological diagnosis. The overarching goals were fairly consistent—enhancing patient autonomy, quality of life, and both motor and cognitive function.

#### Overview of included studies and service settings

Among the 64 included studies, 54 were conducted in fully public contexts (11–60), while 2 studies described service delivery in mixed healthcare systems, such as Norway (91% public, 9% private) (56) and the United States, where public facilities operate within a mixed, insurance-driven system (57, 61). Only one study explicitly reported a fully private context, involving a Japanese vocational adaptation program for individuals with cognitive decline (49). In 7 studies, the service setting was either unclear or not deducible from the information provided (19, 23, 33, 36, 37, 62, 63).

Home-based services were described in 23 studies (11, 12, 18, 31–34, 48, 52, 53, 57–60, 63–69), involving occupational, physical, or cognitive rehabilitation and psychological support delivered in patients’ homes. Outpatient services were used in 18 studies (14, 15, 21, 25, 27, 28, 38–40, 42, 45–47, 55, 56, 59, 60, 63), particularly for group interventions, cognitive training, and psychotherapy. Community services, such as day centers, group activities, peer support, and structured home visits, were described in 9 studies (43, 44, 47, 50, 51, 53, 56, 57, 70).

Hospital-based or specialized rehabilitation center services were documented in 13 studies (13, 16, 21–26, 62, 71), particularly for intensive multidisciplinary programs in the subacute phase. The organization of interventions was monodisciplinary in 19 studies (11, 12, 14, 15, 17, 18, 20, 33, 35–39, 48, 52, 59, 63, 65, 72) and multidisciplinary in 27 studies (13, 16, 21–23, 25–31, 34, 40, 42, 47, 58, 61, 62, 69, 71, 73).

The reported outcomes reflect a combination of those targeted by the study and secondary outcomes that emerged during implementation. Primary outcomes typically corresponded to functional recovery, autonomy, participation and quality of life, while secondary outcomes included caregiver burden, service utilization and emotional wellbeing. This distinction, as well as the specific outcomes assessed in each study, is systematically reported in the data extraction table provided in the Supplementary File 1.

**Figure 4:**
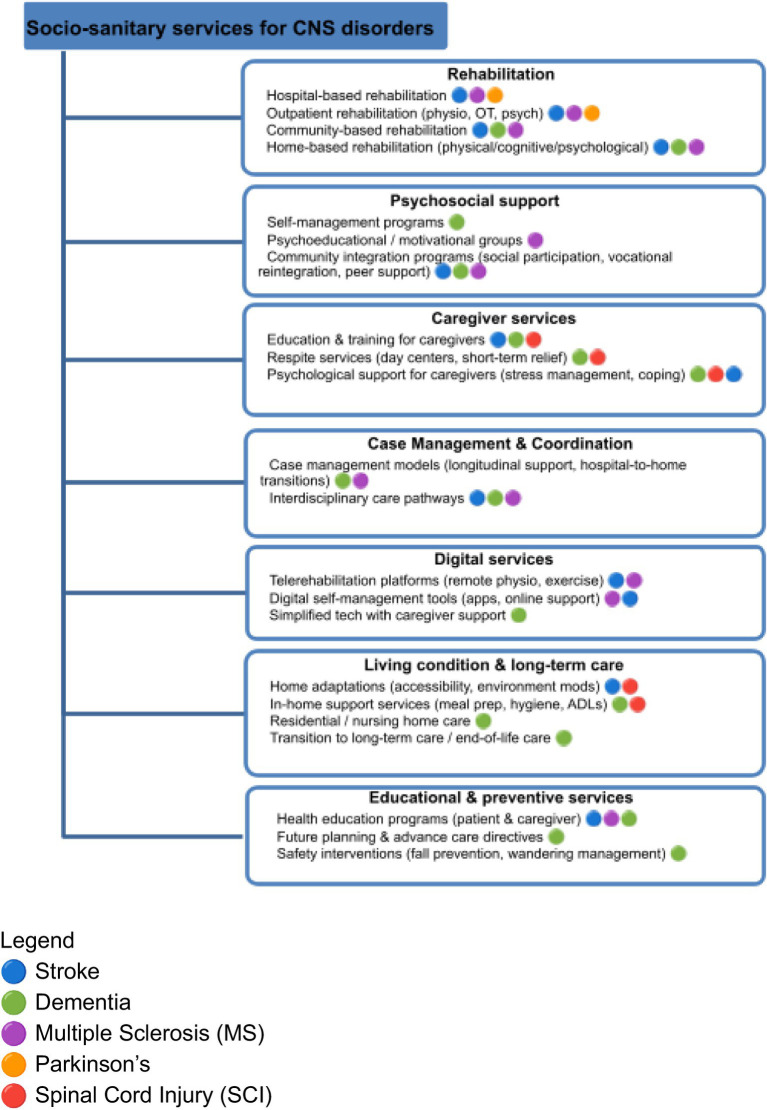
Mapping of social and health services for CNS disorders.

Stroke was the most frequently studied condition, with study types including randomized and controlled designs other than observational studies. In contrast, progressive neurological conditions such as dementia, Parkinson’s disease, multiple sclerosis, and spinal cord injury were less represented and more frequently investigated through small-scale or pilot studies. Conclusions are interpreted in light of the strength and quality of the available evidence for each neurological condition, as summarized in (Table 2), with findings based on limited or heterogeneous evidence framed as exploratory rather than confirmatory. Across all neurological conditions, long-term outcomes beyond one year were rarely assessed. Equity-related dimensions such as socioeconomic status, geographical access, and digital literacy were inconsistently addressed. Moreover formal economic or cost effectiveness evaluations were largely absent (Figure 4).

**Table 2:** Mapping of social and health services with results and limitations for CNS disorders.

Neurological disease	Socio-sanitary services identified	Outcomes	Limitations and evidence strength
Stroke	Hospital-based multidisciplinary rehabilitation Outpatient rehabilitation (physio, OT, cognitive therapy) Home-based rehabilitation and telerehabilitation Community reintegration programs Psychological and social support Caregiver education and involvement	Improved motor performance (TUG, 6MWT, UPDRS) Enhanced ADL independence (BI, FIM) Better QoL (EQ-5D, PDQ-39) Reduced caregiver burden and depressive symptoms	Evidence strength: Moderate to high, supported by multiple studies including randomized and controlled designs, predominantly with moderate risk of bias and consistent findings across service models. Limited long-term follow-up Poor continuity after discharge (hospital→home gap) Lack of standardized coordination models Few studies on cost-effectiveness and equity
Dementia	Home-based cognitive rehabilitation Case management and care coordination Self-management and functional goal-setting programs Community activities and day centers Caregiver education, respite, and psychological support Residential and long-term care	Improved daily functioning (DAD), autonomy, goal achievement (GAS) Reduced depression and anxiety (HADS, BDI) Greater social participation Fewer hospitalizations, better continuity of care	Evidence strength: Moderate to high, supported by multiple studies including randomized and controlled designs, predominantly with moderate risk of bias and consistent findings across service models. Case management not systematized (project-based) Few studies integrating technology Caregiver stress and training needs persist Scarce long-term outcome data
Multiple sclerosis (MS)	Structured physical exercise (aerobic, resistance, balance, gait) Combined physical + psychoeducational group interventions Home-based telerehabilitation or remote supervised programs Interdisciplinary person-centered rehabilitation	Improved balance, mobility, endurance, and fatigue reduction Increased motivation, self-efficacy, and psychological wellbeing	Evidence strength: Low to moderate, primarily derived from small-scale interventions and observational studies, with moderate risk of bias and limited long-term follow-up. Heterogeneous interventions and outcome measures Few economic or longitudinal evaluations Limited inclusion of caregivers • Small sample sizes, often single-center
Parkinson’s disease	Multidisciplinary rehabilitation programs Occupational therapy and ADL training Goal-oriented interventions (GAS, COPM) Caregiver participation in planning	Improved ADL performance (UPDRS II, COPM) Higher satisfaction and goal achievement Enhanced personal motivation	Evidence strength: Low, based on a very limited number of studies, mostly pilot or observational in design; findings should be interpreted as exploratory and hypothesis-generating. Very limited number of studies Short follow-up durations Lack of psychosocial or community interventions
Spinal cord injury (SCI)	Fragmented rehabilitation and social service pathways Environmental/home adaptations Limited caregiver support and counseling	Reported improvement mainly in accessibility and daily autonomy where services existed	Evidence strength: Low, based on a small qualitative evidence base; conclusions are exploratory and primarily descriptive of service fragmentation rather than effectiveness. Very few studies; mostly qualitative No structured integrated care or case management High caregiver stress and poor coordination between medical and social sectors

### Condition-specific findings

#### Stroke: physical rehabilitation and telerehabilitation

In stroke patients, physical exercise programs, multidisciplinary rehabilitation, and telerehabilitation were the mainstay and they were associated with significant improvements in motor performance (TUG, 6MWT, UPDRS) (13–20, 23, 24, 27–30, 62, 72), ability in activities of daily living (BI, FIM) (14, 15, 19, 21, 25, 26, 31, 71), quality of life (EQ-5D, PDQ-39) (16, 17, 19, 36, 64), and emotional wellbeing (HADS, BDI) (25, 26, 38, 39, 71). Additional positive impacts consisted in social reintegration (21, 34) and reduction in caregiver burden (31, 32). However, interventions focused solely on psychological or social support showed less consistent results, with some studies reporting partial benefits (39, 40) and others highlighting limitations related to continuity and personalization of care (34, 40).

#### Dementia: Case-management and home-based cognitive programs

For patients with dementia, five service models were identified, including self-management programs (45, 46, 50, 51), home-based cognitive rehabilitation (48, 52, 54, 65, 67), case management (46, 49, 53, 55, 66, 74), and community activities (47, 56). Primary outcomes included improvements in daily functioning (DAD) (48, 65), achievement of functional goals (GAS) (52, 65), reduction in depression (HADS, BDI) (45, 52, 65), and increased social participation (50, 51). In particular, home-based programs with therapist support promoted autonomy and task management (48, 52, 65), while case management services improved access to care (46, 53), reduced hospitalizations (74), and enhanced continuity of care (49, 66).

#### Multiple sclerosis: structured physical and psychoeducational programs

In multiple sclerosis (MS), identified services could be grouped into three main categories. The first includes five studies (57–60, 68) evaluating structured physical exercise programs, such as aerobic, resistance, balance, and gait training, conducted either in rehabilitation centers (59, 60, 63, 68, 69) or at home (57, 63), with or without professional supervision. Results showed improvements in mobility, balance, physical endurance, and perceived fatigue (57, 60, 63, 68), along with psychological benefits like increased sense of control and motivation (59, 60, 68). The second category includes two studies (59, 69): one integrated physical activity with psychoeducational and motivational group components based on Social Cognitive Theory, while the other analyzed psychosocial adaptation in Jordanian patients. The third category is a single study (68) that evaluated an intensive interdisciplinary person-centered approach rehabilitation program in a hospital setting. Patients reported improvements in motivation and autonomy.

#### Parkinson’s disease: goal-oriented rehabilitation

In Parkinson’s disease, preliminary evidence suggests improvements in activities of daily living (COPM, UPDRS II) (11, 12), personal satisfaction, and achievement of functional goals (GAS) (11).

#### Spinal cord injury

In spinal cord injuries (SCI), the only qualitative study reported limitations regarding fragmented services and lack of structured follow-up, leading to a negative impact on the quality of life and long-term autonomy (61).

#### Caregiver involvement across conditions

Caregivers played a central role in many studies, especially in home settings or individualized interventions. These occurred across studies regarding dementia (46, 48, 50, 54), Parkinson’s (11, 12), and also stroke (31, 32). Their role consisted in active involvement in goal setting and care planning led to perceived benefits and better disease understanding. However, many caregivers—particularly in SCI (61) and dementia (48, 49, 66)—reported high stress levels, lack of training, and absence of formal support.

#### Digital services

In digital services for stroke (36, 37, 64) and MS (63), challenges emerged with technology use among older adults and lack of motivation without direct interaction.

## Discussion

This systematic review synthesized 64 studies evaluating integrated sociosanitary interventions for individuals with central nervous system (CNS) disorders, including stroke, dementia, Parkinson’s disease, multiple sclerosis (MS), and spinal cord injury (SCI). A range of diverse models of service delivery was examined starting from individual physical and cognitive rehabilitation to case management and community-based initiatives. Moreover the studies provided a variety of conduct settings of these services such as hospital, home, outpatient, and digital contexts. The analysis resulted in the unfolding of several consistent themes concerning both the effectiveness and limitations of current practices. Moreover null or mixed findings were reported in several studies, particularly where implementation barriers, such as limited resources, poor coordination, or insufficient follow-up, limited fidelity or continuity.

The predominance of stroke-related studies likely reflects stroke’s high global prevalence, its recognition as a major public health priority, and the existence of established rehabilitation pathways that attract greater research funding and policy attention, particularly in high-income countries. On the other hand this reflects the need for equal awareness and involvement in the spectrum of socio-sanitary progress for the diseases with less evidence, in particular Parkinson’s and SCI. As a consequence the findings regarding these diseases are insufficient to draw confirmatory conclusions, but should be viewed in the spectrum of hypothesis-generating.

### Integration and coordination

Despite the claim for integrated care, true longitudinal integration was the exception rather than the rule. Overall, effectiveness appeared to depend less on diagnosis and more on service characteristics, including personalization, continuity, caregiver involvement, and coordination across care settings. This being the case even in publicly funded systems—where the majority of studies were conducted (11–60)—integration was often pilot-based, short-term, or tied to specific professionals.

The most frequently observed form of “integration” was multidisciplinary rehabilitation. This was more frequent in the stroke populations (13–18, 20–22, 24–30, 62, 71, 72) with combined programs such as physiotherapy, occupational therapy, and occasionally psychological support. These findings can be interpreted in light of global frameworks such as the WHO Rehabilitation 2030 initiative and the Integrated Care for Older People (ICOPE) model, both of which emphasize person-centered, coordinated, and longitudinal care pathways (3–5).

Most interventions identified in this review partially aligned with these principles but failed to achieve full system-level integration. In specific only a few systems ensured continuity after initial recovery phase and discharge (19, 21, 26), with studies where even well-designed hospital programs left patients with limited support once they returned home (34, 40).

Moreover, the SCI study (61) underscored this gap of fragmented socio-sanitary landscape without any aid of clear guidance. Unfortunately, when this was put into context of the environmental barriers, insurance limitations, and perceived clinical incompetence the sensation only worsened (61).

One important thing that patients often mentioned is the concept of case management, or more correctly the lack of it. The role and potential of case managers were highlighted in particular in the dementia group, even though this study nonetheless fell into the “exceptions” group with the presence of a single dedicated professional rather than embedded institutional practices. Nonetheless in these studies case management models (46, 49, 53, 55, 66, 74) stood out for their ability to maintain contact over time, respond flexibly to evolving needs and even help to reduce avoidable hospitalizations (74). All this led to smoother transitions and better alignment between services. This highlights the role of case-management functions—sustained contact, flexible response, navigation—as classic public-health coordination levers that can, with proper funding mechanisms, lower potentially avoidable hospital use, while improving access and continuity.

All this being said, the combination of fragmentation and lack of coordination add up to lack of adherence at the least from the clinical standpoint and at a public health perspective, system inefficiency. Therefore, aiming to stop this vicious cycle, embedding end-to-end pathways that span hospital-to-home is a necessity.

### Caregiver burden: indispensable yet under-supported

The involvement of caregivers not only helped with implementation but also contributed to better planning, emotional stability, and even clinical outcomes thanks to sustaining day-to-day routines (11, 65).

Yet, this was only the minority, as in many cases caregiver needs were addressed only indirectly. Stress, lack of respite, and limited training were common concerns, especially in dementia and SCI studies (48, 49, 61, 66). This is a significant oversight, considering that a caregiver’s wellbeing directly affects sustainability and adherence.

Equally important is the fact that these circumstances put at risk the caregiver’s own health and even render them a population-level risk factor for premature institutionalization and lost productivity. Thus it is important that caregiver training, respite and psychological support should be viewed and treated as preventive public-health priorities, not as add-ons.

### The lack and potential of personalized care

Among the different types of socio-sanitary services, the ones that truly made a difference were those that placed the patient at the center of care. When the socio-sanitary service was adapted to each patient’s goals, daily realities, environment, there was shown not only better physical outcomes but also patient refer more engagement and motivation during their recovery (31–34, 48, 52, 53, 57–60, 63–68). This meant for the patients to be seen through their diagnosis and along their legitimate needs and goals (52, 65, 68). A particularly effective model of personalized care is described in home-based services across stroke, multiple sclerosis and dementia population (52, 57, 58, 63, 65, 68). Patients receiving care in their own environment often reported higher motivation and adherence, especially when interventions were tailored to individual goals. In MS, for instance, programs that combined home-based physiotherapy with periodic professional supervision led to improvements in endurance and reductions in fatigue (57, 58, 63). One study (68) further demonstrated the value of structured personalisation by incorporating goal-setting through the Canadian Occupational Performance Measure (COPM). In dementia care, involving caregivers in tailoring activities to the specific needs of each household added another layer of relevance and effectiveness (52, 65). Yet, not all services are designed with flexibility in mind. Too often patients are part of rigid programs that cannot adapt to their changing needs, or that overlook the role of family and caregivers in everyday care (38, 39, 48, 52, 58). As a consequence even in well-intentioned interventions patients refer to feeling excluded. This observation opens a broader issue regarding the patient’s own “holistic health habitat” and how much the system is really structured around it, from planning and delivery to ongoing evaluation.

### Digital tools: promising, but not a substitute

Technology-based interventions appeared in a subset of stroke and MS studies (18, 24, 57, 58, 63). These included telerehabilitation platforms and remote exercise programs, many of which improved most of all endurance and access. In particular adherence and engagement tended to be higher when supervision was available, even only intermittently (24, 58, 63).

On the other hand unsupervised or purely digital formats came with challenges, so much so that several MS studies reported low motivation, difficulties maintaining routines, and dropout over time (59, 69).

Particular care in implementing these services was necessary in dementia, where technological tools required simplification or caregiver involvement due to cognitive limitations (48, 65). Overall, these findings suggest that digital tools work best as complementing strategies rather than replacements of human interaction. Their effectiveness does not only depend on technological designs, but also on how well they are integrated into care models and how well they adapt to individual capacities of the patients, providing ongoing guidance.

### Strengths and limitations

One of the most persistent issues was inconsistency in outcome measurement. Stroke interventions relied heavily on physical performance indicators, but gave less attention to quality of life or emotional status. In MS, outcomes focused on fatigue, balance, and endurance, while psychological and caregiver outcomes were rarely assessed. Only one study used a goal-oriented measure like COPM to track personalized progress (68). The same goes for dementia studies which included functional scales like DAD and GAS (48, 52, 65), but showed similar gaps in tracking emotional and caregiver-centered outcomes. This variability in outcome measures limits comparability.

Most included studies were conducted in high-income countries with established public health systems. Evidence from low- and middle-income settings was scarce, limiting the generalizability of findings across diverse health-system contexts. Moreover, although several studies reported outcomes with clear economic relevance, such as reduced hospitalizations, delayed institutionalization, or improved return-to-work, formal economic evaluations and analyses of financing mechanisms were largely absent. As a result, the sustainability and scalability of socio-sanitary rehabilitation services remain insufficiently addressed in the current literature. Additionally many studies featured short follow-up periods, particularly in progressive conditions like dementia or MS. Another barrier to scaling integrated models into everyday practice was the lack of formal economic evaluations, leaving important questions about cost-effectiveness unanswered.

Patient and caregiver involvement in the design of services was also limited. Most interventions were professionally led, with little use of co-design approaches that could improve acceptability and adherence. Barriers linked to geography, socioeconomic status, or digital literacy were rarely explored, despite their relevance to service accessibility.

These findings highlight the need for future research to focus not only on clinical outcomes, but also on long-term impact, user experience, and system-level feasibility.

Publication bias may be present, as studies reporting positive outcomes are more likely to be published. Negative or neutral outcomes may also be underrepresented in the included literature. This may be partly explained by the limited duration of the follow-up in many studies and by the lack of standardized service models and outcome measures across socio-sanitary interventions. Moreover, heterogeneity in service design and evaluation frameworks may reduce the sensitivity of studies to detect delayed or less favorable effects, particularly in progressive neurological conditions.

In several studies, critical feedback primarily addressed challenges in implementation rather than questioning the conceptual value of the services, indicating that outcomes may be influenced by contextual and organizational factors.

The geographical concentration of evidence in high-income countries further limits external validity.

## Conclusion

This review reveals that while integrated sociosanitary services for CNS conditions show promise, implementation remains partial and inconsistent, and effectiveness appears to depend more on personalization, continuity, and coordination within end-to-end care pathways than on diagnosis alone. Additionally at a primordial level this review points out the lack of evidence in the scope of socio-sanitary services for equally important and life-altering diseases such as multiple sclerosis, Parkinson’s and spinal cord injury.

The move toward integrated care demands addressing key gaps such as limited long-term follow-up, patient/caregiver education, personalization and persistent equity issues. These factors, in turn, are limited by the absence of standardized service frameworks and robust economic evaluations.

Moreover implementation itself regardless of these gaps is hindered by the lack of evidence on cost-effectiveness, scalability, and sustainability across different health-system contexts limits policy translation. This underscores the need for robust economic evaluations followed by sustainable financing strategies in order to move beyond experimentation and toward long lasting integration that is both cost-effective and inclusive. Future service development should prioritize promising integrated care pathways such as systematic case management, hybrid digital-human service models, community-based services and socio-integration policies.

## Data Availability

The original contributions presented in the study are included in the article/Supplementary material, further inquiries can be directed to the corresponding authors.
