# Supplementary material for: Innovative socio-sanitary rehabilitation models in central nervous system disorders: a systematic review
Source: Front Public Health. 2026 Feb 13;14:1742821. doi: 10.3389/fpubh.2026.1742821 (PMC12947126; doi:10.3389/fpubh.2026.1742821)
Supplement: Supplementary file 1 [file Table_1.docx]

Table S1 **:** Search strings for each data base

| **Database** | **Stringa di ricerca** |
| --- | --- |
| PubMed | ("cerebrovascular disorders"[MeSH Terms] OR "Dementia"[MeSH Terms] OR "Basal Ganglia Diseases"[MeSH Terms] OR "Amnesia, Transient Global"[MeSH Terms] OR "Akinetic Mutism"[MeSH Terms] OR "Thalamic Diseases"[MeSH Terms] OR "Dementia, Vascular"[MeSH Terms] OR "Leukoencephalopathies"[MeSH Terms] OR "Diffuse Cerebral Sclerosis of Schilder"[MeSH Terms] OR "Neuromyelitis Optica"[MeSH Terms] OR "Myelitis, Transverse"[MeSH Terms] OR "Multiple Sclerosis"[MeSH Terms] OR "Spinocerebellar Degenerations"[MeSH Terms] OR "Myelitis"[MeSH Terms] OR "Muscular Atrophy, Spinal"[MeSH Terms] OR "Amyotrophic Lateral Sclerosis"[MeSH Terms] OR "Parkinsonian Disorders"[MeSH Terms] OR "Dyskinesias"[MeSH Terms] OR "Multiple System Atrophy"[MeSH Terms] OR "Supranuclear Palsy, Progressive"[MeSH Terms] OR "Tauopathies"[MeSH Terms] OR "Myotonic Disorders"[MeSH Terms] OR "Paresis"[MeSH Terms] OR "Quadriplegia"[MeSH Terms] OR "Pseudobulbar Palsy"[MeSH Terms] OR "Paraplegia"[MeSH Terms] OR "Hemiplegia"[MeSH Terms] OR "Chorea"[MeSH Terms] OR "Intellectual Disability"[MeSH Terms] OR "Communication Disorders"[MeSH Terms] OR "TDP-43 Proteinopathies"[MeSH Terms]) AND ("Community Resources"[MeSH Terms] OR "Psychosocial Deprivation"[MeSH Terms] OR "social conditions"[MeSH Terms] OR "social environment"[MeSH Terms] OR "social isolation"[MeSH Terms] OR "Social Marginalization"[MeSH Terms] OR "Social Norms"[MeSH Terms] OR "Social Vulnerability"[MeSH Terms] OR "Socialization"[MeSH Terms] OR "Sociodemographic Factors"[MeSH Terms] OR "Socioeconomic Factors"[MeSH Terms] OR "social capital"[MeSH Terms]) AND ("Rehabilitation"[MeSH Terms] OR "Halfway Houses"[MeSH Terms] OR "Early Intervention, Educational"[MeSH Terms] OR "Neurological Rehabilitation"[MeSH Terms] OR "continuity of patient care"[MeSH Terms] OR "custodial care"[MeSH Terms] OR "day care, medical"[MeSH Terms] OR "foster home care"[MeSH Terms] OR "night care"[MeSH Terms] OR "nursing care"[MeSH Terms] OR "holistic health"[MeSH Terms] OR "Household Work"[MeSH Terms]) NOT "Hospital care" |
| Scopus | (TITLE-ABS-KEY("cerebrovascular disorders") OR TITLE-ABS-KEY("Dementia") OR TITLE-ABS-KEY("Basal Ganglia Diseases") OR TITLE-ABS-KEY("Amnesia, Transient Global") OR TITLE-ABS-KEY("Akinetic Mutism") OR TITLE-ABS-KEY("Thalamic Diseases") OR TITLE-ABS-KEY("Dementia, Vascular") OR TITLE-ABS-KEY("Leukoencephalopathies") OR TITLE-ABS-KEY("Diffuse Cerebral Sclerosis of Schilder") OR TITLE-ABS-KEY("Neuromyelitis Optica") OR TITLE-ABS-KEY("Myelitis, Transverse") OR TITLE-ABS-KEY("Multiple Sclerosis") OR TITLE-ABS-KEY("Spinocerebellar Degenerations") OR TITLE-ABS-KEY("Myelitis") OR TITLE-ABS-KEY("Muscular Atrophy, Spinal") OR TITLE-ABS-KEY("Amyotrophic Lateral Sclerosis") OR TITLE-ABS-KEY("Parkinsonian Disorders") OR TITLE-ABS-KEY("Dyskinesias") OR TITLE-ABS-KEY("Multiple System Atrophy") OR TITLE-ABS-KEY("Supranuclear Palsy, Progressive") OR TITLE-ABS-KEY("Tauopathies") OR TITLE-ABS-KEY("Myotonic Disorders") OR TITLE-ABS-KEY("Paresis") OR TITLE-ABS-KEY("Quadriplegia") OR TITLE-ABS-KEY("Pseudobulbar Palsy") OR TITLE-ABS-KEY("Paraplegia") OR TITLE-ABS-KEY("Hemiplegia") OR TITLE-ABS-KEY("Chorea") OR TITLE-ABS-KEY("Intellectual Disability") OR TITLE-ABS-KEY("Communication Disorders") OR TITLE-ABS-KEY("TDP-43 Proteinopathies")) AND (TITLE-ABS-KEY("Community Resources") OR TITLE-ABS-KEY("Psychosocial Deprivation") OR TITLE-ABS-KEY("social conditions") OR TITLE-ABS-KEY("social environment") OR TITLE-ABS-KEY("social isolation") OR TITLE-ABS-KEY("Social Marginalization") OR TITLE-ABS-KEY("Social Norms") OR TITLE-ABS-KEY("Social Vulnerability") OR TITLE-ABS-KEY("Socialization") OR TITLE-ABS-KEY("Sociodemographic Factors") OR TITLE-ABS-KEY("Socioeconomic Factors") OR TITLE-ABS-KEY("social capital")) AND (TITLE-ABS-KEY("Rehabilitation") OR TITLE-ABS-KEY("Halfway Houses") OR TITLE-ABS-KEY("Early Intervention, Educational") OR TITLE-ABS-KEY("Neurological Rehabilitation") OR TITLE-ABS-KEY("continuity of patient care") OR TITLE-ABS-KEY("custodial care") OR TITLE-ABS-KEY("day care, medical") OR TITLE-ABS-KEY("foster home care") OR TITLE-ABS-KEY("night care") OR TITLE-ABS-KEY("nursing care") OR TITLE-ABS-KEY("holistic health") OR TITLE-ABS-KEY("Household Work")) AND NOT TITLE-ABS-KEY("Hospital care") AND PUBYEAR > 2012 AND PUBYEAR < 2026 |
| Web of Science | (("cerebrovascular disorders" OR "Dementia" OR "Thalamic Diseases" OR "Leukoencephalopathies" OR "Diffuse Cerebral Sclerosis of Schilder" OR "Neuromyelitis Optica" OR "Myelitis, Transverse" OR "Multiple Sclerosis" OR "Spinocerebellar Degenerations" OR "Myelitis" OR "Muscular Atrophy, Spinal" OR "Amyotrophic Lateral Sclerosis" OR "Parkinsonian Disorders" OR "Dyskinesias" OR "Multiple System Atrophy" OR "Supranuclear Palsy, Progressive" OR "Tauopathies" OR "Myotonic Disorders" OR "Paresis" OR "Quadriplegia" OR "Pseudobulbar Palsy" OR "Paraplegia" OR "Intellectual Disability" OR "Communication Disorders") AND ("Community Resources" OR "Psychosocial Deprivation" OR "social conditions" OR "social environment" OR "social isolation" OR "Social Marginalization" OR "Social Norms" OR "Social Vulnerability" OR "Socialization" OR "Sociodemographic Factors" OR "Socioeconomic Factors" OR "social capital") AND ("Rehabilitation" OR "Halfway Houses" OR "Early Intervention, Educational" OR "Neurological Rehabilitation" OR "continuity of patient care" OR "custodial care" OR "day care, medical" OR "foster home care" OR "night care" OR "nursing care" OR "holistic health" OR "Household Work") NOT "Hospital care") (All Fields) AND (2024 OR 2023 OR 2022 OR 2021 OR 2020 OR 2019 OR 2018 OR 2017 OR 2016 OR 2015 OR 2014 OR 2013) |

Table S2 & S3 : Data extraction table

| **Main Author** | **Publication year** | **Title** | **Nationality** | **Type of study** | **Type of health system studied** | **Disease/disability studied** | **Participants** | **Age group** | **Gender** | **Other demographic characteristics** | **Type of service** | **Level of care studied** |
| --- | --- | --- | --- | --- | --- | --- | --- | --- | --- | --- | --- | --- |
|  |  |  |  |  |  |  | PARKINSON |  |  |  |  |  |
| Joanna Cholewa [11] | 2016 | Continuation of full time employment as an inhibiting factor in Parkinson’s disease symptoms | Poland | Quasi-experimental study (non-randomized intervention study) | mixed | Parkinson’s Disease (ICD-10 code: G20) | 70 | 60 years | 22 women    48 men | N/A | Physiotherapy | Community-based, outpatient setting |
| Swink et al. [12] | 2020 | Health-related quality of life changes after the merging yoga and occupational therapy for Parkinson’s disease program: A mixed-methods study | USA (Colorado) | Mixed-methods convergent design (Quantitative pre-post repeated measures + Qualitative focus groups) | Private (Community-based program in a private athletic club, outside the public health system) | Parkinson’s Disease (ICD-10 code: G20) | 18 participants recruited  17 completed quantitative assessments  16 participated in focus groups | 64–82 years (mean 71.7 years) | 10 men  8 women | - 100% White - Mostly retired - Majority held college-level education | Yoga (adaptive, group-based)    Occupational therapy (fall risk management, individual action plans) | Community-based, outpatient setting |
|  |  |  |  |  |  |  | Spinal cord injury |  |  |  |  |  |
| Kern et al.[13] | 2019 | Understanding the Changing Health Care Needs of Individuals Aging With Spinal Cord Injury | USA | Qualitative study (phenomenological design using semi-structured interviews) | Public health system | T91.3 (Sequelae of injury of spinal cord) | 49 interviewees  41 individuals with SCI    8 caregivers | Mean age = 73.4 years (range 61–89) | Not fully specified for SCI participants    Among caregivers: 5 women, 3 men | - - Mean time since injury: 10.8 years   - Caregivers were all non-paid family caregivers | Health and rehabilitation service utilization, including:   - Outpatient rehabilitation - Home health services - Care coordination and insurance navigation - Assistive device provision - Participation in follow-up rehab clinics | Community-based |
|  |  |  |  |  |  |  | **STROKE** |  |  |  |  |  |
| Gallacher et al.[14] | 2018 | A conceptual model of treatment burden and patient capacity in stroke | UK (Scotland) | Qualitative study (phenomenological approach using semi-structured interviews) | Public health system | I63 (Cerebral infarction)    I61 (Intracerebral hemorrhage)    I64 (Stroke, not specified as hemorrhage or infarction) | 29 individuals with stroke were interviewed | 68 years | 16 men    13 women | N/A | Healthcare and rehabilitation services after stroke, specifically focusing on:   - Acute hospital care - Outpatient rehabilitation - Primary care follow-up - Community-based rehabilitation and home adaptations | Mixed   - Hospital - Ambulatory/outpatient - Home (domiciliary) - Community setting |
| Cobley et al.[15] | 2013 | A qualitative study exploring patients’ and carers’ experiences of Early Supported Discharge services after stroke | Uk(Nottingham) | Qualitative study (semi-structured interviews with thematic analysis) | Public health system | I63 (Cerebral infarction), I64 (Stroke, not specified), or I61 (Intracerebral hemorrhage) – general stroke classification | 42 total: 27 stroke patients and 15 carers | Patients mean age = 69.85 years    Carers mean age = 72.79 years | Among carers: 87% were women    Patient gender distribution not specified | Carers were exclusively spouses of patients | Early Supported Discharge (ESD) services after stroke — a model that provides coordinated, intensive, multidisciplinary rehabilitation at home, replacing continued inpatient care for patients with mild to moderate stroke | Community-based settings in the patients’ homes, following early discharge from hospital |
| Glickman et al.[16] | 2018 | Clients with stroke and non-stroke and their guardians’ views on community reintegration status after in-patient rehabilitation | United States and Malawi | Quantitative observational study (cross-sectional and comparative with two consecutive yearly cohorts) | Public health system | Primarily stroke (I63, I64) and non-stroke neurological conditions (e.g., G37-G83 range: spinal cord dysfunction, Guillain-Barré Syndrome) | 36 clients    32 guardians | Clients: 27 to 91 years old    Mean age = 55 years | Clients: 22 males, 14 females    Guardians: Gender not fully detailed | Guardians were mostly close family members (spouses, children, or parents) | Inpatient rehabilitation | Ambulatory |
| Ashaie et al.[17] | 2022 | Complexity of Participation Post-Stroke: Longitudinal Assessment of Community Participation, Positive Affect, Social Support and Functional Independence | United States | Quantitative longitudinal study using retrospective analysis of an observational dataset | Mixed | Not specified directly in ICD-10 terms; participants had stroke (primarily ischemic, including aphasia subgroup) | 544 stroke survivors (229 with aphasia, 315 without aphasia) | Mean age: 67.78 years | 283 female participants  261 male participants | Mean years of education: 12.51    Race/ethnicity:   - 76.29% White - 17.10% Black - 4.41% Hispanic - 2.21% Other | Comprehensive stroke rehabilitation care delivered during inpatient hospitalization, followed by naturalistic community reintegration | Hospital |
| Sinclair et al.[18] | 2014 | Developing stroke-specific vocational rehabilitation: a soft systems analysis of current service provision | United Kingdom (Nottingham, England) | Qualitative study using soft systems methodology (SSM) and mixed methods (interviews, observation, focus groups, documentary analysis) | Public health system | Not specified directly, but the study concerns stroke (ICD-10: I60–I69) | Not specified | Working-age stroke survivors (exact ages not provided) | Not reported | N/A | Stroke-specific vocational rehabilitation (SSVR) services aimed at supporting return to work (RTW) | Mixed |
| Askew et al.[19] | 2020 | *Differential Effects of Time to Initiation of Therapy on Disability and Quality of Life in Patients With Mild and Moderate to Severe Ischemic Stroke* | USA | Prospective longitudinal cohort study | Comprehensive stroke care center in a large metropolitan city | Not explicitly listed, but the study includes ischemic stroke and transient ischemic attack (TIA) | Analyzed: 553 participants | Mean age: 66.6 years | 51.9% male    48.1% female | 64.4% White, 31.7% Black, 4.0% Other    88.8% had ischemic stroke; 11.2% had TIA    Median NIHSS score: 3 [IQR: 1–6]    77.2% had hypertension, 30.6% diabetes, 18.1% prior ischemic stroke    98.4% could ambulate before stroke (with or without assistance) | Acute therapy services (physical therapy, occupational therapy, speech-language therapy) initiated during in-hospital acute care | Hospital setting (inpatient acute care) |
| Mattioli et al.[20] | 2014 | Early Aphasia Rehabilitation Is Associated With Functional Reactivation of the Left Inferior Frontal Gyrus: A Pilot Study | Italy | Pilot randomized controlled trial (with neuroimaging) | Public health care setting | Not specified, but applies to I63 (Ischemic stroke) with R47.0 (Aphasia) | 12 stroke patients  10 healthy controls | Adults under 80 years old; exact mean age not reported | Rehab group: 2 women, 4 men    Non-rehab group: 3 women, 3 men | Right-handed first-ever left hemisphere (MCA territory) stroke    Free of prior neurological or psychiatric conditions | Early, intensive aphasia language therapy | Hospital |
| Martin et al.[21] | 2023 | Early Opportunities to Explore Occupational Identity Change: Qualitative Study of Return-To-Work Experiences After Stroke | New Zealand | Qualitative study using reflexive thematic analysis within a realist research framework | Public health system | Not explicitly provided, but applies to I60–I69 (Cerebrovascular diseases / Stroke) | 31 stroke survivors | Mean 57 years (range 25–76) | 15 male (48%)    16 female (52%) | Ethnicity: 84% NZ European, 6% Māori, 3% Pasifika, 6% Other    61% were working (part-time or full-time) at the time of interview | Vocational rehabilitation and return-to-work support following stroke, with a focus on early conversations, identity exploration, and tailored support pathways | Mixed settings |
| Lin et al.[22] | 2019 | Effect of Social Support and Health Education on Depression Scale Scores of Chronic Stroke Patients | Taiwan | Randomized controlled interventional study | Public healthcare | Not explicitly stated; applicable codes:    I63 (Cerebral infarction)    I64 (Stroke, not specified)    F32.9 (Depressive episode, unspecified – related to post-stroke depression) | 62 stroke patients (31 intervention group, 31 control group) | Mean age = 63.1 years | 44 males (71%)    18 females (29%) | Education: 37.1% college level, others with varying education    53.2% had caregivers    46.8% reported low leisure satisfaction | Routine rehabilitation + Social support and health education | Outpatient/ambulatory |
| Liu-Ambrose et al.[23] | 2015 | Exercise Training and Recreational Activities to Promote Executive Functions in Chronic Stroke: A Proof-of-Concept Study | Canada | Randomized controlled proof-of-concept study (ancillary to a larger multi-site RCT) | Public | Not explicitly stated; applicable:    I63 (Cerebral infarction)    I69 (Sequelae of cerebrovascular disease) | Randomized: 28    Completed analysis: 24 (INT group: 11; D-INT group: 13) | Mean age: 65.2 years | 60% male (15 out of 25 participants reported)    INT group: 36% male    D-INT group: 78.6% male | MoCA score mean: 23.0 (suggesting mild cognitive impairment)    Participants lived in the community and were able to walk >10 meters    Participants had completed formal rehabilitation | Exercise Training and Recreational Activities | Community setting |
| O’Callaghan et al.[24] | 2024 | Experiences and preferences of people with stroke and caregivers, around supports provided at the transition from hospital to home: a qualitative descriptive study | Ireland | Qualitative descriptive study using semi-structured interviews and reflexive thematic analysis | Public health care system | Not explicitly listed, but applicable codes:    I63 (Cerebral infarction)    I64 (Stroke, unspecified) | 16 participants total    9 people with stroke (PWS)    7 caregivers (CGs) | PWS: Aged 47 to 79 | PWS: 6 males, 3 females    CGs: 6 females, 1 male | N/A | Post-stroke transitional support services, including discharge planning, community rehabilitation, early supported discharge (ESD), emotional support, and care coordination | Mixed |
| May et al.[25] | 2023 | Experiences of Adults with Stroke Attending a Peer-Led Peer-Support Group | Canada | Qualitative descriptive study using semi-structured interviews and constructivist grounded theory | Community-based and peer-led | Not explicitly stated; applicable codes include:    I63 (Cerebral infarction)    I69 (Sequelae of cerebrovascular disease) | 11 adults with stroke | Range: 41 to 83 years    Average age bracket: 65–69 years | Majority female (exact numbers not stated) | Diverse ethnic backgrounds: Asian, Hispanic, Caucasian, Southeast Asian | Community-organized and volunteer-run support group including social gatherings, games, shared meals, informal support, and mutual aid | Community-based setting (e.g., public community center or non-profit housing common area) |
| Koositamongkol et al.[26] | 2013 | Factors influencing functional recovery in patients with acute ischemic stroke | Thailand | Prospective observational study | Public | Not explicitly stated, but applicable code is:    I63 (Cerebral infarction / Acute ischemic stroke) | 141 | Mean age: 61.98 years    30–50 years: 20.5%    51–70 years: 51.8%    71–90 years: 27.7% | 57.4% male  42.6% female | N/A | Acute stroke care services, including thrombolytic therapy (rt-PA), early mobilization, multidisciplinary support, and stroke unit management | Hospital |
| Lindley et al. (The ATTEND Collaborative Group)[27] | 2017 | Family-led rehabilitation after stroke in India (ATTEND): a randomised controlled trial | India | Randomised controlled trial (RCT) — prospective, open-label with blinded endpoint (PROBE design) | Public | Not explicitly reported, but applies to:    I63 (Cerebral infarction)    I61 (Intracerebral hemorrhage) | 1,250 patients (623 intervention, 627 control) | Range: 18–95 years    Mean: ~58 years | 67% male    33% female | Broad socioeconomic diversity, with 15% earning <5000 INR/month    Caregivers included spouses (41%), sons/daughters (51%), and others | Family-led stroke rehabilitation program, including:    In-hospital caregiver training (1 hour/day for ~3 days)    Up to 6 home visits and 2 months of telephone support    Training in task-specific ADLs, communication, mobility, and caregiver empowerment | Mixed:    Initiated in hospital setting    Continued via home-based (domiciliary) visits |
| Tramonti et al.[28] | 2014 | Functional status and quality of life of stroke survivors undergoing rehabilitation programmes in a hospital setting | Italy | Quantitative longitudinal observational study | Public | Not explicitly stated, but applicable:    I63 (Cerebral infarction)    I61 (Intracerebral hemorrhage) | 29 | Mean age = 63 years | 17 men (59%)  12 women (41%) | N/A | Multidisciplinary hospital-based inpatient neurorehabilitation program | Hospital |
| Fama et al.[29] | 2016 | Group Therapy as a Social Context for Aphasia Recovery: A Pilot, Observational Study in an Acute Rehabilitation Hospital | United States | Pilot observational within-subject study | Public | Not explicitly reported, but applicable:    I69.3 (Sequelae of cerebral infarction with aphasia)    I69.8 (Other sequelae of cerebrovascular disease) | 10 | - 1. 87 years | 6 females    4 males | All were native English speakers with no history of mental illness or major sensory deficits    Most had suspected comorbid verbal apraxia | Speech-language therapy, comparing group therapy sessions vs. individual sessions in patients with acute, severe non-fluent aphasia | Hospital |
| Manning et al.[30] | 2024 | How do speech and language therapists enact aphasia psychosocial support in Ireland? A cross-sectional online survey informed by normalization process theory | Ireland | Cross-sectional online survey of clinicians (quantitative descriptive study) | Public | Not explicitly stated, but relevant codes include:    I69.3 – Sequelae of cerebral infarction with aphasia    The survey focused on aphasia of any aetiology | 54 | Not applicable | Not reported | 54% of respondents had ≥10 years of SLT experience    65% had worked ≥4 years with aphasia patients    Respondents worked across various settings: acute/subacute hospitals (39%), community care (35%), inpatient rehab/stroke units (35%), early supported discharge (17%) | Psychosocial support for people with aphasia, as delivered or facilitated by SLTs (via counseling, supportive listening, social reintegration, referrals) | Services spanned hospital, community, outpatient, early supported discharge, and long-term care settings |
| Balasooriya-Smeekens et al.[31] | 2020 | How primary care can help survivors of transient ischaemic attack and stroke return to work: focus groups with stakeholders from a UK community | United Kingdom | Qualitative study using focus groups and framework analysis | Public | Not specified; applicable:    I63 (Cerebral infarction)    G45 (Transient ischaemic attack) | 18 | 18–65 years | Not fully detailed | Diverse occupational backgrounds and return-to-work statuses (not working, part-time, full-time, voluntary work) | Primary care and community-based vocational rehabilitation services — especially how primary care interfaces with return-to-work support after TIA/stroke | Community-based primary care and neurorehabilitation services |
| Moore et al.[32] | 2024 | I’ve still got a job to go back to”: the importance of early vocational rehabilitation after stroke | Australia | Qualitative study using semi-structured interviews (embedded in a randomized controlled trial) | Public | I63 (Cerebral infarction)    I69.3 (Sequelae of stroke with aphasia or other deficits) | 16 | 23–71 years | 9 female (56%)  7 male (44%) | All participants were working at the time of their stroke    6 participants (38%) had post-stroke aphasia    Diverse employment types and return-to-work outcomes    Mean time post-stroke at recruitment: 55 days (range 4 days to 3 months) | Early vocational rehabilitation (VR) delivered by occupational therapists | Mixed – initiated during clinical rehabilitation and delivered via home or outpatient follow-up |
| Bērziņa et al.[33] | 2016 | Living in Latvia after stroke: the association between functional, social and personal factors and the level of self-perceived disability—a cross-sectional study | Latvia | Cross-sectional study with retrospective data collection | Public | I60–I67 | 255 stroke survivors (from an initial cohort of 600) | Median age: 64 years (range: 22–92) | 52% male    48% female | 74.9% lived in cities    22% lived alone    19.7% were working at the time of the survey    Preferred language: 73.7% Latvian, 26.3% Russian    Time since discharge from rehabilitation: median 20 months    38% had higher education | In-patient post-acute neurorehabilitation services | Hospital-based in-patient rehabilitation |
| White et al.[34] | 2016 | Predictors of health-related quality of life in community-dwelling stroke survivors: a cohort study | Australia | Prospective longitudinal cohort study | Public | Not explicitly stated; applicable codes:    I63 (Cerebral infarction)    I64 (Stroke, unspecified) | 134 | Range: 37–96 years  Mean: 75 years | 55% female, 45% male | 47% married, 37% widowed    61% did not live alone    12% had history of depression    22% had baseline depression; 47% had baseline anxiety | Community-based stroke follow-up — no active intervention; observational study on HRQoL evolution | Mixed |
| Delhey et al.[35] | 2024 | Neighborhood Resources and Health Outcomes Among Stroke Survivors in a Population-Based Cohort | United States | Longitudinal population-based cohort study (observational, mixed methods: Cox regression & linear models) | Not applicable — the study did not assess a healthcare service but analyzed neighborhood environmental effects | Not explicitly stated; appropriate codes:    I63 (Cerebral infarction)    I61 (Intracerebral hemorrhage) | 1786 participants included in mortality and recurrence analyses    1284 participants included in 3-month outcome analyses | Median age: 64 years | 55% male  45% female | 62% Mexican American, 38% non-Hispanic White    Varied socioeconomic status, education, and comorbidity profiles | Not a service — study investigated the impact of neighborhood resource density (e.g., stores, community centers, eateries) on post-stroke outcomes | N/A |
| Ng et al.[36] | 2013 | Long-term Efficacy of Occupational Lifestyle Redesign Programme for Strokes | Hong Kong (China) | Retrospective matched cohort study | Public | Not explicitly stated; appropriate code:    I63 (Cerebral infarction)    I69.3 (Sequelae of stroke with residual deficits) | 50 | 22–80 years | 26 male (52%)  24 female (48%) | N/A | Occupational Lifestyle Redesign Programme (OLSR) — added to conventional outpatient OT | Ambulatory (outpatient clinic) |
| Egan et al.[37] | 2014 | Participation and Well-Being Poststroke: Evidence of Reciprocal Effects | Canada | Prospective longitudinal cohort study | Not applicable — this study did not assess a service delivery model but tracked outcomes post-discharge into the community | Not explicitly stated; applicable codes:    I63 (Cerebral infarction)    I64 (Stroke, unspecified) | 67 | 33–88 years (mean: 64.8 ± 13.3) | 58% male (n = 39)  42% female (n = 28) | Most lived with spouse (68.7%) or alone (20.9%) | N/A | Community |
| Tielemans et al.[38] | 2016 | Process evaluation of the Restore4Stroke Self-Management intervention ‘Plan Ahead!’: a stroke-specific self-management intervention | Netherlands | Prospective, mixed-method process evaluation study | Public | Not explicitly mentioned; applicable codes:    I63 (Cerebral infarction)    I64 (Stroke, unspecified) | 58 stroke patients, 28 partners, 19 therapists | Patients: mean age 55.5 years (range: 36–68)    Partners: mean age 57.2 years (range: 45–70) | Patients: 45% male    Partners: 50% male | Patients: 23% employed; 72% cohabiting with partner    Partners: 50% employed; all cohabiting with patient | Stroke-specific self-management intervention (“Plan Ahead!”) — part of the Restore4Stroke program | Ambulatory (outpatient clinic) |
| Joana Matos et al.[39] | 2024 | Professional reintegration of stroke survivors and their mental health, quality of life and community integration | Portugal | Cross-sectional observational study | Not applicable — no structured reintegration service was studied; data was collected from stroke survivors treated in public Stroke Units | Not explicitly stated; applicable codes:    I63 (Cerebral infarction)    I64 (Stroke, unspecified) | 553 | 18–70+ years; stratified into <50, 50–59, ≥60 | 63.1% male (n = 349)    36.9% female (n = 204) | 70.4% married/cohabiting    29.4% in white-collar jobs; 70.6% in blue-collar occupations | No formal service was implemented. The study assessed naturalistic professional reintegration, without structured vocational support | Community-based outcome |
| Takeru Umemura et al.[40] | 2023 | Rate of return to work in patients with stroke under the health and employment support program of Rosai hospitals in Japan | Japan | Retrospective cohort study | Public | I60–I69 (Cerebrovascular diseases including cerebral infarction, cerebral hemorrhage, subarachnoid hemorrhage) | 483 | 20–80 years (median age 50) | 377 males, 106 females | Occupational category: 204 white-collar, 279 blue-collar workers | Health and Employment Support (HES) Program for return to work | Mixed (initially in hospital, later with coordination in workplace and community) |
| Birgitta Langhammer et al.[41] | 2018 | Return to work after specialized rehabilitation—An explorative longitudinal study in a cohort of severely disabled persons with stroke in seven countries | Multinational (Norway, China, USA, Russia, Palestine, Israel, Sweden) | Prospective descriptive longitudinal multicenter study | Public | I63 (Cerebral infarction) / I64 (Stroke, not specified) | 230 | 49 - 64 years | Predominantly male | N/A | Specialized inpatient neurorehabilitation (multidisciplinary) | Primarily hospital-based for the rehabilitation phase, followed by ambulatory and some home-based physical therapy depending on country |
| Emma Westerlind et al.[42] | 2020 | Return to work predicts perceived participation and autonomy by individuals with stroke | Sweden | Retrospective cohort study | Public | I60 (Subarachnoid hemorrhage), I61 (Intracerebral hemorrhage), I63 (Cerebral infarction) | 109 | Adults aged 24–63 years (median age: 53) | 65% male  35% female | N/A | Return to work (RTW) evaluated as an outcome, not a formal service — no specific vocational program was studied | Not applicable — RTW was assessed post-hospitalization, in the community context |
| Sophie Lehnerer et al.[43] | 2019 | Social work support and unmet social needs in life after stroke: a cross-sectional exploratory study | Germany | Cross-sectional exploratory study | Public | I63 (Cerebral infarction – stroke) | 57 patients + 24 caregivers | Mean age: 70 years (±10) | 58% male    42% female | Median years of education: 14 (IQR 12–17)    Median net household income: €2200/month    Time since stroke: 2–3 years    42% of patients had an mRS score ≥3 (moderate to severe disability) | Social work support in long-term stroke care | Primarily hospital and rehabilitation settings; limited post-discharge/community-based care |
| Neil Heron et al.[44] | 2017 | Stroke Prevention Rehabilitation Intervention Trial of Exercise (SPRITE) – a randomised feasibility study | United Kingdom | Randomised feasibility study (pilot RCT) | Public | I63 (Cerebral infarction – minor stroke), G45 (Transient Ischemic Attack) | 15 | Mean age: 69 years | 10 male    5 female | Majority were retired    Most participants were married    Predominantly from less disadvantaged areas    Only one participant had university-level education    Most were ex-smokers and low alcohol consumers | Home-based cardiac rehabilitation adapted for stroke/TIA patients (“The Healthy Brain Rehabilitation Manual”), with or without pedometer | Home (domiciliary) |
| Charlotte Wassenius et al.[45] | 2023 | The centrality of work in everyday life after stroke: A qualitative study of long-term stroke survivors | Sweden | Qualitative study (semi-structured interviews, thematic analysis) | Public | I63 | 9 | 45–60 years at time of stroke  64–77 years at time of interview | 5 women    4 men | N/A | Long-term vocational rehabilitation and occupational engagement | Community (with hospital-originated support) |
| Veronica Ntsiea et al.[46] | 2014 | The effect of a workplace intervention programme on return to work after stroke: A randomised controlled trial | South African | Randomised Controlled Trial (RCT) | Public | I63 | 80 stroke survivors (40 intervention, 40 control) | 18–60 years (mean age: ~45 years) | 51% male (n=41)  49% female (n=39) | Majority were breadwinners (63%)    Occupational profiles: 55% blue collar, 45% white collar    Educational level varied: from Grade 7 to degree holders | Workplace-based vocational rehabilitation intervention program | Community + Workplace |
| Moon KT, Jang W, Park HY, Jung M, Kim JB. [47] | 2022 | The Effects of Occupation-Based Community Rehabilitation for Improving Activities of Daily Living and Health-Related Quality of Life of People with Disabilities after Stroke Living at Home: A Single Subject Design | South Korea | Single-subject experimental study (A-B-A design) | Public | I61, I63 | 3 | 37–59 years | 2 males, 1 female | N/A | Occupation-based community rehabilitation (CBR) | Domiciliary |
| Ying-Tzu Tseng[48] | 2024 | The effects of rehabilitation potential on activities of daily living in patients with stroke in Taiwan: a prospective longitudinal study | Taiwan | Prospective longitudinal study | Public | I61, I63 | 101 | Adults ≥20 years | Not explicitly stated | 67.3% married  64.4% high school education or higher  43.9% had sufficient income  25.7% had prior stroke | Inpatient rehabilitation for post-stroke recovery | Hospital setting (inpatient acute care) |
| Clarke DJ, Powers K, Trusson D, Craven K, Phillips J, Holmes J, et al. [49] | 2023 | The RETurn to work After stroKE (RETAKE) trial: Findings from a mixed-methods process evaluation of the Early Stroke Specialist Vocational Rehabilitation (ESSVR) intervention | United Kingdom | Mixed-methods process evaluation (as part of a randomized controlled trial) | Public | I63 | 181 participants received the intervention in the trial    qualitative data collected from  58 participants  22 occupational therapists  10 employers | Adults of working age (primarily 18–65) | Not specified | N/A | Early Stroke Specialist Vocational Rehabilitation (ESSVR) | Community-based and home visits; follow-up after hospital discharge |
| Katie E. Powers et al.[50] | 2023 | Exploring the Association between Individual-Level Attributes and Fidelity to a Vocational Rehabilitation Intervention within a Randomised Controlled Trial | United Kingdom (England and Wales) | Randomised controlled trial + Quantitative observational analysis | Public | Stroke(Cerebrovascular Accident) | 39 | N/A | N/A | Mean OT experience:  17.3 years as OT  9.3 years in stroke rehab  3.5 years in VR | Early Stroke Specialist Vocational Rehabilitation (ESSVR) | Community based intervention for stroke survivors. |
| Rosbergen ICM et al.[51] | 2017 | Qualitative investigation of the perceptions and experiences of nursing and allied health professionals involved in the implementation of an enriched environment in an Australian acute stroke unit | Australia | Primary qualitative study | Public | Stroke (acute phase) ICD-10 code: I63 (Cerebral infarction), I64 (Stroke, not specified as hemorrhage or infarction) | Participants (staff): 10 participants: 7 nurses, 3 allied health professionals | Age range: under 40 to over 40 years | Majority female (exact numbers not stated) | Experience in stroke unit: <2 years to >5 years  All but 2 had attended preparatory education session | Environmental enrichment intervention embedded in an acute stroke unit to increase patient activity and engagement through structured communal activities, family involvement, and staff education. | Hospital based, inpatient setting |
|  |  |  |  |  |  |  | **DEMENTIA** |  |  |  |  |  |
| Catherine Quinn et al. [52] | 2016 | A pilot randomized controlled trial of a self -management group intervention for people with early-stage dementia (The Smart study) | North Wales, UK | Single-blind pilot Randomised controll trial | Public | An ICD-10 diagnosis of Alzheimer's disease, vascular dementia or mixed Alzheimer's disease and vascular dementia | TOTAL 24 participants Intervention 13 participants Treatment as usual(TAU) 11 participant | Intervention: age range 52-88 (mean age 75.2)  TAU: age range 62-88 (mean age 76.1) | Male 18 Female 6 | Mostly UK nationality, level of education mostly collage/university, 17 patients have other co-morbid conditions | Psychosocial and educational group based intervention | Community-based, outpatient setting |
| Clement Pigmouguet et al. [53] | 2016 | Benefits of occupational Therapy in Dementia Patients: Findings from a Real-World Observational Study | France | Observational study | Public | An ICD-10 diagnosis of Alzheimer's disease, vascular dementia or mixed Alzheimer's disease and vascular dementia | Total number of participants 421 | Age range 65-85 (mean age 82.2) | Female 302 patients    Male 119 patients | N/A | Home-based occupational therapy (OT) intervention | Community-based, home-delivered support |
| A. Marijke van Haefen-van Dijk et al. [54] | 2015 | Community day care with carer support versus usual nursing home-based day care: effects on needs, behavior, mood, and quality of life of people with dementia | Netherlands | Prospective(non-randomized intervention trial) | Public | An ICD-10 diagnosis of Alzheimer's disease, vascular dementia or mixed Alzheimer's disease and vascular dementia | Total number of participants 138 dyads    -70 participants in the community Day Care (CO)    -68 participants in the Nursing Home-based Day care(NH) | Mean age for the Community day care participants 79,3    Nursing Home based day care mean age is 81,3 | Co group 61% female    NH group 56% female | N/A | Community based service based on the MCSP model (Meeting Centres support Programe)    Nursing home based day care delivered within nursing home | CO- community based service    NH- nstitution based service |
| Mayumi Sakamoto et al.[55] | 2013 | Comparing the effects of different individualized music interventions for elderly individuals with severe dementia | Japan | Randomized controlled trial (RCT) with three directions: interactive music, passive music, and no-music control group. | Public: delivered in publicly accessible group homes and a special dementia hospital in Japan; services operate within the national long-term care insurance system | Severe Alzheimer's disease | Total participants: 39    Control group:13  Passive music group: 13    Interactive music group:13 | Control group mean age: 81    Passive group mean age: 81,1    Interactive group mean age: 81,2 | 32 women  7 men | N/A | Psychosocial intervention: Individualized music therapy | Long term care facility based (institutional community) |
| Dorota Szczesniak et al. [56] | 2018 | Does the community-based combined Meeting Center Support Programme (MCSP) make pathway to day-care activities easier for people living with dementia? A comaprison in three European contries | Europe | Exploratory qualitative implementation study (cross-country design) | Public | Dementia | Not specified | Not specified | Not specified | Demographic data not included: study focuses on system-level implementation and expert-based analysis rather than individual participant outcomes | Community based psychosocial support (Meeting Centre Support Programme) | Post-diagnostic, community outpatient care |
| Shizuko Omote et al.[57] | 2023 | Experiences with Support at workplace for people with Young Onset Dementia: A qualitative evaluation of being open about dementia | Japan | Observational, cross-sectional study | Private sector(workplace-led support systems) | Young onset dementia (YOD) | 10 workplace representatives | Age range of the workplace representatives: 40s-60s | 7 male  3 female | Each participants represented a workplace employing someone with YOD  Industries included healthcare, manufacturing, eductaion, transport and staffing.  No direct data on employees with YOD | A workplace-based support system | Low-intensity, socially integrated, community-based support, situated withing the work environment |
| Annelies van Rijn et al.[58] | 2019 | Linking DemenTalent to meeting centres for people with dementia and their caregivers: a process analysis into facilitators and barriers in 12 Dutch meeting centres | Netherlands | Qualitative implementation study | Public | Dementia-mild to moderate stages | 22 stakeholders | Age ranges:   - 20-29: 1 person - 30-39:1 person - 40-49: 4 persons - 50-59: 9 persons | Female 17, Male 5 | - 1. The demographic data is for the stakeholders, not of people with dementia.   2. Roles of the stakeholders are: project leaders, meeting centre maagers, municipality representatives, volunteer's workplace staff, external coaches and a person with dementia who was also a project ambassador. | A community based volunteering program | Non-clinical, community based psychosocial care, inagrated in the ambulatory post-diagnostic pathway and delivered outside medical institutions. |
| Johannes Oesterholm et al. [59] | 2024 | Occupational therapists' experiences of working with people with dementia: a qualitative study | Sweden | Cross-sectional qualitative study | Public health and social care system. Participants worked in muncipal elder care, primary care and hospital/geraitric wards under Sweden's publicy funded system | Dementia: including mild to advanced stages | 10 occupational therapists | Average age: 49 years    Age range: 22-63 years | All partcipants were female | - - Experience as an occupational therapist 2 to 29 years(avarage 16 years)   - Eductaion:all held a bachelor's degree in occupational therapy   - Most had >5 years of experience working specifically with people with dementia | Occupational therapy interventions for people with dementia in public healthcare and eldercare systems | Community-based and institutional care including: primary care, home-based care, residential elder care settings |
| Justin Chew et al. [60] | 2015 | Outcomes of a multimodal cognitive and physical rehabilitation program for persons with mild dementia and their caregivers: a goal-oriented approach | Singapore | Prospective observational cohort study | Public healthcare setting | An ICD-10 diagnosis of Alzheimer's disease, vascular dementia or mixed Alzheimer's disease and vascular dementia | 55 participants | Mean age: 79.2 years | Gender:   - 46% female - 54% male | N/A | Multimodal Rehabilitation Program, | Ambulatory rehabilitation, delivered in an outpatient hospital setting |
| Georgina Charlesworth et al.[61] | 2016 | Peer support and reminiscence therapy for people with dementia and their family carers: a factorial pragmatic randomised trial | United Kingdom (London, Berkshire, Norfolk and Northamptonshire) | Randomized controlled trial (RCT) | Publicly funded services | Dementia, diagnosed using DSM-IV criteria | 291 dyads(patients with dementia+family carer) | Mean age for carers: 67 years  Mean age for persons with dementia 80 years | Gender of carers:   - 67% female - 33% male     Gender of persons with dementia:   - 53% female - 47% male | Caring duration for carers an avarage of 4+ years | Two psychosocial interventions:   1. Carer supporter programme 2. Joint group therapy for people with dementia and their carers | Non-clinical, community based psychosocial care, inagrated in the ambulatory post-diagnostic pathway and delivered outside medical institutions. |
| Helen Tam-Tham et al. [62] | 2016 | Provision of dementia-related services in Canada: a comparative study | Canada | Qualitative comparative study | Public healthcare system | An ICD-10 diagnosis of Alzheimer's disease, vascular dementia or mixed Alzheimer's disease and vascular dementia | 34 healthcare providers | N/A | N/A | Only professional roles were described:   - 12 physicians - 13 specialists - 9 case managers | The service includes:   1. Future planning, eductaional and social support 2. Home care and respite services 3. Day programes 4. In-home support services 5. Safety and behavioral management 6. Long-term care transition support | Community based AND ambulatory care. |
| Carina Wattmo et al. [63] | 2014 | Solitary living in Alzheimer's disease over 3 years: association between cognitive and functional impairment and community based services | Sweden | Prospective, a 3-year longitudinal observational cohort study | Public health and social care system | Alzheimer's disease | 1021 individuals | - - **Solitary living group:** mean age at diagnosis 77.1 years   - **Cohabitating group**: mean age at diagnosis 74,2 years | - - **Soliatry living group:**   85% female  15% male   - - **Cohabiting group:**   53% female  47% male | 355 partcipants (35%) were living alone, mostly older women with higher functional impairment | Community-based dementia services including:   1. Home-help services: Non-medical, practical support (e.g., meal prep, hygiene) 2. Nursing home placement: Admission to skilled residential care | Community and residential care |
| Rose-Marie Droes et al.[64] | 2019 | Utilization, effect and benefit of the individualized Meeting Centres Support Program for people with dementia and caregivers | Netherlands | Prospective, comparative study | Public service | Dementia(mild to moderate dmentia), most participants had Alzheimer's disease | 136 persons with dementia   - 65 in the iMCSP group - 71 in the regular MCSP     128 family caregivers | Persons with dementia: mean age 80 years    Caregivers: mean age 69 years | Persons with dementia  **MCSP**   - female 59% - male 41%     **iMCSP**   - female 66% - male 44%     Caregivers  **MCSP**   - 74% female - 26% male     **iMCSP**   - 71% female - 29% male | Most of the carers were spouses or children | Psychosocial support model integrated into community care settings | Ambulatory community care |
| Netta Van't Leven et al.[65] | 2018 | Working mechanisms of dyadic, psychosocial, activating interventions for people with dementia and informal caregivers: a qualitative study | Netherlands | A qualitative study , based on semi structured interviews | Public | Dementia | - Total dyads interviewed: 34 - People with dementia: 27 - Caregivers:34 | People with dementia:   - mean age: 78 years - age range:62-93     Cargivers   - age range: 53-75 years | Gender of people with dementia:  65% male  35% female | Most of the caregivers were either partners and adult children | Dyadic psychosocial interventions aimed at:   - Enhancing activity participation - Reducing caregiver burden - Increasing self-efficacy and adaptation capacity | Home-based, community psychosocial interventions combined with ambulatory care |
| Samira Sangi et al.[66] | 2020 | The design and effectiveness of educational package based on increased activity and cognitive, emotional and neuro-muscular activities of daily living in the elderly with mild cognitive impairment | Iran | Quasi-experimental study | Public sector care | Mild Cognitive impairment(MCI)-considered a pre-dementia condition | 30 elderly individuals Randomly assigned:   - 15 to the experimental group - 15 to the control group | Mean age for the experimental group: 75.73 years    Mean age for the control group: 75.6 years | 50% women  50% men | Length of institutionalization for the experiemental group: 5.6 years    Length of institutionalization for the control group: 5.2 years | Multimodal cognitive rehabilitation program | The service was delivered at a nursing home |
| Janne Rosvik et al.[67] | 2021 | We must have a new VIPS meeting soon! Barriers and facilitators for implementing the VIPS practice model in primary health care | Norway | Qualitative analysis, using semi-structured interviews. | Primarily public health services (as per Norway’s tax-funded system), but some private non-profit/for-profit nursing homes were also included (approx. 9% of services). | Dementia | Participants in the study STAFF: total number 17 | Age range:30-65 years | 16 women  1 man | - - **Roles of participants:** Managers, head nurses, project leaders across 10 Norwegian municipalities.   - **Professional background:** All but one were registered nurses; one had administrative training.   - **Settings:** Both public and private nursing homes and domestic care services. | Implementation of the VIPS practice model for person-centred dementia care in domestic nursing and long-term care settings. | - - Community-based domestic care   - Institutional long-term care (nursing homes)   - Day-care centres |
|  |  |  |  |  |  |  | **MULTIPLE SCLEROSIS** |  |  |  |  |  |
| Matthew Plow et al.[68] | 2014 | A formative evaluation of costumized pamphlets to promote physical activity and symptom self-management in women with multiple sclerosis | United states | Randomized controlled pilot study | The intervention is part of a publicly funded health research system | Relapsing-remitting multiple sclerosis(RRMS) | 30 participants | Mean age for the immediate intervention group: 47 years    For the delayed group: 48 years | 100% women | - - The patients had a diagnosis of Relapsing-remitting multiple sclerosis(RRMS)      - - Employment(full/part time): In the immediate group 57%, in the delayed group 50% | Costumized print-based intervention aimed to increasing physical activity and managing sumptoms in women with MS | Community based intervetion, delivered in participants' home with some initial in-person sessions at a clinical or research facility |
| Glattacker M. et al.[69] | 2018 | Rehabilitation use in multiple sclerosis: Do illness reppresentation matter? | Germany | Observational, cross-sectional study | Public healthcare system | Multiple Sclerosis | 590 participants | Mean age: 42 years | Predominantly female | All participants are of a working age | Use of rehabilitation services in Multiple Sclerosis (physical therapy, occupational therapy, other multidisciplinary interventions) | Hospital based outpatient rehabilitation centres56 |
| Niall Russell et al.[70] | 2023 | Experiences of people with multiple sclerosis participating in a social cognitive behavior change physical activity intervention | Ireland | Qualitative study, semi-structured interviews post-intervention | Public | Multiple Sclerosis, relapsing-remitting or mild MS | Total number of participants: 17 | Mean age: 42.88 years | 14 women  3 men | - - **Duration of the MS symptoms:** Mean=7.38 years   - **Expanded Disability status scale:** Mean=3.26   - All participants were ambulatory and able to walk unaided | Group-based physical activity intervention delivered by a public university (University of Limerick) | Community based, non-clinical |
| Andreas Falck Lahelle et al. [71] | 2020 | Group dynamics in a group-based, individualized physiotherapy intervention for people with multiple clerosis: A qualitative study | Norway | Qualitative study  Using:   - 13 video observations of physiotherapy sessions (14 hr 38 min total) - 13 semi-structured interviews with physiotherapists (12 hr 37 min total) - Analyzed using systematic text condensation under an enactive theoretical framework | Public system | Multiple Sclerosis | 40 participants | Age range: 24-77 years    Mean age: 52.2 years | Female 13 (32%), Male 27(68%) | MS type distribution:   - RRMS: 33 (83%) - SPMS: 5 (12%) - PPMS: 2 (5%)   Disease duration (mean ± SD): 10.2 ± 7.9 years (range: 0.5–33) | GroupCoreDIST: A group-based physiotherapy intervention tailored to individual needs in MS patients.   - Combines standardized core stability exercises with individualized difficulty levels, delivered in small group sessions by trained physiotherapists. | Community-based settings within municipal physiotherapy clinics in Norway  Delivered under primary care via publicly funded PTs (with a combination of salary or government reimbursement |
| Evan Mansson Lexell et al.[72] | 2014 | Self-perceived performance and satisfaction with performance of daily activities in persons with multiple sclerosis following interdisciplinary rehabilitation | Sweden | Retrospective pre–post design | Delivered at a public university hospital | Multiple Sclerosis, types included   - RRMS - SPMS - PPMS | 43 participants | Mean age for men: 48 years  Mean age for women:53 years | 27 women  16 men | **Years since MS onset:**   - Men: 15 years - Women: 18 years   **Type of MS:**   - RRMS: 7 - SPMS: 32 - PPMS: 4   **Living situation:**   - Majority lived with someone (spouse or partner) - Some lived alone or with part-time assistance   **Employment:**   - Most were on partial or full disability pension - Very few actively working full-time   **Social support:**   - Varied from no support to assistance several times/week | Inpatient, interdisciplinary rehabilitation program tailored for people with MS   - Individualized, goal-oriented care - Multidisciplinary team working with shared goals (true interdisciplinary model) | Hospital-based inpatient rehabilitation at Skåne University Hospital |
| Saba Yaseen Hyarat et al.[73] | 2019 | Health related Quality of Life among patients with Multiple Sclerosis: The Role of psychosocial adjustment to illness | Jordan | Cross-sectional, descriptive-correlational study | Study conducted in public hospital clinics in Jordan | Multiple Sclerosis | Total participants: 160 | Mean age: 31.2 years Age range 19-45 | Female 126(78,8%) Male 34(21.3%) | - - Most common treatment:   Interferon (91%)   - - Most participants experienced multiple relapses and were regularly hospitalized, indicating moderate disease burden   - Years since MS diagnosis: Mean = 6.7 (range 1–16)   - Hospitalization in past 4 months: 77.5% had been hospitalized   - Relapses in previous year: Mean = 2.2 (SD = 1.2); range = 0–5 relapses   - Employment status:   Employed (government, private, or self): 72.5%  Unemployed: 27.5% | Observational, correlational study measuring psychosocial adjustment and its impact on health-related quality of life (HRQoL) in people with MS. | Outpatient clinics of two public hospitals in Jordan    Data collected in community ambulatory care settings    No specific intervention was implemented, only assessments |
| I. Milivojevic et. Al[74] | 2013 | Utilization of physical rehabilitation among people with multiple sclerosis | Croatia | Observational cross-sectional study | Croatian public health system | Multiple Sclerosis ICD-10 code: G35 | Toatl participants: 63 | Mean age: 38 years (range 21-58) | Female 41(65%), Male 22(35%) | - - Disease duration: Mean = 5.4 years (range: 1–16)   - Hospitalization in prior 2 years: 41.3% received rehab | Investigates the utilization patterns of physical rehabilitation services by people with MS.    Rehabilitation types examined:   - Inpatient rehabilitation - Outpatient rehabilitation   Home-based rehabilitation | Mixed settings:   1. Inpatient rehabilitation centers 2. Outpatient hospital-based clinics 3. Home-based programs |

| **Main Author** | **Detailed description of the service/study** | **Service/study methodology** | **Primary outcomes** | **Secondary outcomes** | **Outcome evaluation tool** | **Follow-up** | **Strength/Significance of the Impact** | **Synthesis of the experiences, perceptions, and satisfaction of users regarding the services received** | **Synthesis of the experiences and perceptions of caregivers and family members** |
| --- | --- | --- | --- | --- | --- | --- | --- | --- | --- |
|  |  |  |  | PARKINSON |  |  |  |  |  |
| Joanna Cholewa[11] | - - **Participants:** Idiopathic Parkinson’s disease (Hoehn and Yahr stage II), no prior physiotherapy.   - **Format:** Gym-based sessions, 2×/week, 45 minutes, for 20 weeks.   - **Focus:** Motor symptoms — bradykinesia, rigidity, postural instability, tremor.   - **Techniques:** Cueing strategies (acoustic, visual, sensory), mental rehearsal, reflex triggering, postural training.   - **Exercises:**     - Positional transitions (supine, side-lying, prone, sitting).     - Gait training (step length, widened base, directional change, rhythmic walking).     - Tremor management through intentional movement.   - **Home component:** Daily exercises with illustrated instructions for reinforcement and adherence. | In a gymnasium setting twice per week for 45 minutes over a 20-week period | 1. Motor symptom severity   - Assessed using the Unified Parkinson’s Disease Rating Scale (UPDRS):   - Part I: Cognitive and emotional functioning  - Part II: Activities of daily living  - Part III: Motor examination  Total score: Combined sum of Parts I, II, and III    2. Quality of life   - Assessed using the Parkinson’s Disease Questionnaire (PDQ-39) - Provides a Summary Index reflecting overall quality of life across eight domains       Results    Group A (Working full-time)  After 20 weeks of physiotherapy:   - UPDRS Part I: Improvement of 10.38% (p = 0.001) - UPDRS Part II: Improvement of 37.47% (p = 0.001) - UPDRS Part III: Improvement of 38.95% (p = 0.001) - UPDRS total (I+II+III): Improvement of 36.70% (p = 0.002) - PDQ-39 (Quality of life): Improvement of 42.46% (p = 0.001)     Group B (Non-working)  After 20 weeks:   - UPDRS Part I: No statistically significant improvement (p = 0.430) - UPDRS Part II: Minor, non-significant improvement (p = 0.370) - UPDRS Part III: Improvement of 25.21% (p = 0.001) - UPDRS total (I+II+III): Improvement of 18.28% (p = 0.001) - PDQ-39: Improvement of 24.77% (p = 0.001) | Full-time was associated with greater improvements in both motor function and quality of life following physiotherapy.   - In the non-working group, only UPDRS Part III (motor symptoms) showed significant improvement, while Parts I and II (cognition/emotion and daily activities) showed minimal or no statistically significant gains. - In contrast, all UPDRS parts improved significantly in the working group, suggesting a broader impact of rehabilitation among employed individuals. | Motor symptoms severity, assessed using the Unified Parkinson’s Disease Rating Scale (UPDRS) — specifically:   - Part I: Intellectual and cognitive status - Part II: Activities of daily living - Part III: Motor examination - Total score (sum of Parts I–III)     Quality of life, evaluated using the Parkinson’s Disease Questionnaire (PDQ-39), which includes eight domains:   - Mobility - Activities of daily living - Emotional well-being - Stigma - Social support - Cognition - Communication - Bodily discomfort | N/A | Group A (Working full-time)   - UPDRS Part I: Significant improvement (p = 0.001) - UPDRS Part II: Significant improvement (p = 0.001) - UPDRS Part III: Significant improvement (p = 0.001) - UPDRS total (I + II + III): Significant improvement (p = 0.002)      - PDQ-39 (Quality of life): Significant improvement (p = 0.001)     Group B (Non-working)   - UPDRS Part I: Not significant (p = 0.430) - UPDRS Part II: Not significant (p = 0.370) - UPDRS Part III: Significant improvement (p = 0.001) - UPDRS total (I + II + III): Significant improvement (p = 0.001)      - PDQ-39: Significant improvement (p = 0.001)     Statistical analysis included independent t-tests, one-way ANOVA, and Bonferroni post hoc tests. | N/A | N/A |
| Swink et al. [12] | - - **Participants:** 18 individuals with Parkinson’s disease.   - **Setting:** Weekly 90-minute sessions over 8 weeks at a community athletic club.   - **Activities:** Adaptive yoga + structured occupational therapy (education, fall risk, goal planning).   - **Evaluation:** PDQ-8 at baseline, mid-, and post-intervention; focus groups for qualitative feedback. | Group sessions held in-person at a community location (athletic club)  Fall prevention content integrated into yoga practice | No statistically significant differences in PDQ-8 scores across baseline, mid-intervention, and post-intervention assessments (F(2,32) = 1.60, p = 0.22, partial η² = 0.09).  However, qualitative findings from the focus groups indicated that participants perceived improvements in all eight HRQoL domains covered by the PDQ-8, suggesting subjective benefits not fully captured by the quantitative measures. | Qualitative themes highlighting perceived improvements in emotional well-being, social participation, physical function (such as flexibility and balance), self-efficacy in managing Parkinson’s disease symptoms, and reduced fear of falling. Participants also reported feeling more empowered, motivated, and connected to others living with Parkinson’s disease through the group format of the sessions | PDQ-8 (quantitative HRQoL scale) | 1-month | N/A | - - Sessions were experienced as supportive, empowering, and tailored.   - Participants reported feeling emotionally uplifted, physically stronger, and more capable of managing daily challenges.   - The combination of yoga and occupational therapy was appreciated for addressing both physical and functional needs.   - Individualized action plans, fall prevention education, and peer interaction were seen as key strengths.   - Benefits extended beyond physical health, improving mental well-being and fostering social connectedness. | N/A |
|  |  |  |  | Spinal cord injury |  |  |  |  |  |
| Kern et al.[13] | - - **Design:** Semi-structured interviews with individuals aging with spinal cord injury (SCI) and their caregivers.   - **Focus:** Health care needs, service utilization, and access barriers.   - **Services discussed:** Home health care, outpatient rehabilitation (PT/OT), assistive devices, caregiver support, insurance issues.   - **Interview method:** Mostly in-person; three conducted by telephone.   - **Content guide:** Topics included health status, aging-related changes, service use, energy and activity levels, functional changes, social participation, mentorship, and caregiver needs. | Most interviews were conducted in person, except for three that were done by telephone due to distance. | The study found four major themes regarding the needs of patients with SCI, each with subthemes:   - Health literacy   - Wide variability among participants in understanding their medical conditions, medications, and rehabilitation needs.   - Differences in problem-solving capabilities related to managing health.      - Health services   - Barriers to accessing needed health services, especially due to insurance limitations (e.g., having to choose between home health services and outpatient therapy due to insurance reimbursement restrictions).   - Perceived inadequacy of healthcare professionals’ knowledge about long-term SCI management.   - Disconnect between patient needs and healthcare provider goals.      - Functional changes with age   - Reduced energy, more frequent falls, worsening bowel/bladder management, and increased dependency.   - Loss of independence in activities of daily living and instrumental activities.   - Mixed attitudes toward future planning, with some participants being proactive and others feeling pessimistic.      - Environment   - Both built environment (home accessibility modifications, transportation) and human environment (social support, caregiver relationships) significantly influenced participation, independence, and perceived quality of life. | Impact of health literacy on service use and independence  Participants with higher health literacy and better problem-solving skills were better able to access services, advocate for themselves, and maintain higher levels of participation.    Distinction between aging effects and SCI-related decline  Many participants struggled to differentiate whether their functional declines (e.g., falls, fatigue, skin breakdown) were due to "normal" aging or to SCI-specific progression, suggesting a need for better patient education during follow-up care. | N/A | N/A | N/A | - - Experiences with health services among individuals aging with SCI were mixed, with many reporting dissatisfaction linked to insurance barriers, financial strain, and limited specialist expertise.   - Some prioritized basic needs like medications over therapy due to reimbursement issues, creating care gaps.   - Many felt that providers overlooked their rehabilitation goals and did not adapt care to changing needs.   - Support was often perceived to drop sharply after acute rehabilitation, leaving patients and caregivers to manage fragmented services independently.   - Those with strong problem-solving skills or social support reported better experiences.   - Overall, health systems were seen as poorly prepared for the complex, evolving needs of people aging with SCI, leading many to rely heavily on self-advocacy and external support. | - - Caregivers of individuals aging with SCI reported that services were poorly coordinated and often limited by insurance coverage.   - Physical and emotional strain was common, especially due to reduced professional support after initial rehabilitation.   - Caregivers frequently assumed multiple roles without formal training or respite, leading to exhaustion and anxiety.   - Poor communication with health professionals and inadequate long-term planning added to caregiver stress.   - Financial pressures, including costly home modifications, further increased the burden.   - Strong family ties and community networks helped partially buffer the strain.   - Overall, caregivers viewed the healthcare system as unprepared for the long-term needs of patients and families. |
|  |  |  |  | **STROKE** |  |  |  |  |  |
| Gallacher et al.[14] | - - **Design:** Semi-structured interviews with 29 stroke survivors (conducted 2014–2016, at home).   - **Focus:** Impact of health and social care service configuration, quality, and coordination on treatment burden and patient capacity.   - **Services discussed:** Acute hospital care, outpatient rehabilitation (PT, OT, SLT, dietetics), primary care follow-up, community stroke teams, social care (home adaptations, mobility aids, domiciliary care).   - **Analysis method:** Framework analysis (based on Normalization Process Theory) for first 15 interviews; thematic analysis for the remaining 14.   - **Procedures to enhance rigor:** Iterative refinement of interview guide, triangulation, independent coding, and team discussions. | In person semi-structured interviews | The study resulted in the development of two detailed taxonomies—one for treatment burden and another for patient capacity—as well as a conceptual model that illustrates the dynamic interaction between care workload, service deficiencies, and a patient’s capacity to self-manage.    **Treatment burden taxonomy (based on 29 interviews):**   1. The workload of healthcare: managing appointments, organizing medication, attending therapies, navigating benefits, coordinating between services.      1. Care deficiencies: lack of follow-up (especially for milder cases), poor communication between providers, inadequate information, limited access to transport, and financial strain for home help and adaptations.     These burdens were categorized into four phases of stroke management:   1. Making sense of stroke and planning care 2. Interacting with others (e.g., health professionals) 3. Enacting management strategies (e.g., rehab, appointments) 4. Reflecting on progress and future care     **Patient capacity taxonomy:**  Six key factors influenced participants' ability to cope with treatment:  Personal attributes and skills (e.g., resilience, cognitive function, health literacy)    Physical and cognitive abilities (e.g., aphasia, balance problems)    Support network (family, friends, caregivers)    Financial status (ability to afford care or transport)    Life workload (other health conditions, caregiving responsibilities)    Environment (e.g., housing accessibility, transport availability) | Disparities in care based on stroke severity   - Participants with milder stroke symptoms reported significantly less follow-up, fewer services, and often felt neglected. - Those with more severe impairments (e.g., needing mobility aids, home care) were more likely to receive comprehensive community-based rehabilitation and coordinated services.     Disconnect between patient expectations (e.g., support, follow-up, clarity on medications) and healthcare system design    Burden of fragmented care coordination   - Participants described significant effort spent navigating between services, arranging appointments, and managing conflicting medical advice. - Some were forced to act as their own care coordinators or rely heavily on family, especially for medication management, benefits, and transport.     Negative psychological impact of poor service delivery   - Uncoordinated discharges, lack of home follow-up, or financial barriers led to frustration, anxiety, and helplessness. - Some participants intentionally disengaged from services out of disillusionment or perceived lack of benefit.     Caregiver strain (indirectly identified)   - Although caregivers were not formally analyzed, their presence during interviews revealed informal caregiving burdens, especially in the context of gaps in formal care services. - Participants with limited family support were especially vulnerable to treatment burden.     Role of financial strain in reducing patient capacity   - Participants often had to self-fund home modifications or private therapy, especially when NHS waiting lists were long or services were deemed insufficient. - Those with fewer financial resources faced barriers to participation, such as inability to travel or hire help.     Importance of psychosocial supports   - Participants who had access to peer stroke groups, charities, or community volunteers often coped better, even when formal care was lacking. - Those who lacked such support reported increased isolation and reduced motivation for rehabilitation. | N/A | N/A | The credibility of findings were supported using qualitative validation methods, including:   - Triangulation of data sources and analysts - Data saturation checks - Independent coding of transcripts by multiple researchers - Thematic consistency confirmed through iterative analysis | - - Acute hospital care, especially on stroke wards, was generally viewed positively due to competent and supportive staff.   - Experiences after discharge varied, with some reporting comprehensive care while others faced fragmented support, long waits, and financial barriers.   - Many with mild disabilities felt abandoned, receiving little to no follow-up or rehabilitation.   - Dissatisfaction with primary care follow-up was common, particularly when communication with specialists was poor or home visits were difficult.   - Access to community rehabilitation was often limited, poorly timed, and required patients to navigate complex systems alone.   - Challenges adapting to home life included difficulties with housing modifications, assistive devices, and emotional support.   - Participants with well-coordinated home therapy or strong social networks reported better experiences.   - Overall, satisfaction was highly variable, with systemic fragmentation cited as a major barrier to long-term recovery. | - - Caregivers often took on unofficial care coordination roles, managing services, follow-ups, prescriptions, home modifications, and benefits access.   - The transition from hospital to home was frequently abrupt and poorly supported, increasing caregiver burden.   - Many experienced emotional, physical, and financial strain, along with frustration, anxiety, and isolation.   - Satisfaction was higher when community stroke teams provided consistent home-based rehabilitation, though such support was rare. |
| Cobley et al.[15] | - - **Eligibility criteria for ESD:** Barthel Index ≥14/20, ability to transfer with minimal assistance, stable medical condition, rehabilitation goals identified within 14 days post-stroke.   - **Intervention:** Early Supported Discharge (ESD) with coordinated input from physiotherapists, occupational therapists, speech and language therapists, and rehabilitation nurses.   - **Rehabilitation delivery:** Up to four therapy sessions per day, seven days a week, for up to six weeks; intensity comparable to inpatient care.   - **Access visits:** Conducted before discharge to arrange necessary equipment and home adaptations.   - **Focus:** Home-based rehabilitation targeting mobility, transfers, outdoor walking, and reintegration into daily activities. | Hospital (initial stroke care and eligibility assessment)    Home (domiciliary) and community (for the ESD intervention and follow-up rehabilitation)    Ambulatory (for transition to outpatient rehab services post-ESD, though these were sometimes poorly coordinated) | **Among Patients Receiving ESD:**   - Highly positive perceptions of receiving rehabilitation at home, with participants appreciating the familiarity, privacy, and relevance of practicing functional tasks in their own environment.      - Most patients valued the intensity and frequency of therapy (up to four visits/day, 7 days/week), which promoted functional recovery and gave a sense of security during a major life transition.      - Satisfaction with equipment provision was common      - Speed of initial ESD team response was praised      - The end of ESD (at six weeks) was described as abrupt, and transitions to further community services were often poorly coordinated or delayed.     **Among Carers:**   - Being that carers had felt clear lack of structured information and education on stroke, long-term care and secondary prevention, the ESD therapy sessions were appreciated for the temporary respite they gave them. - Nevertheless some felt the visits were too short to meaningfully reduce their load. - Carers often felt excluded from care planning, despite being the primary source of long-term support at home. | Impact of ESD on carer burden and role strain   - Carers reported experiencing significant physical and emotional exhaustion, often feeling isolated, underprepared, and unsupported in managing complex care at home. - Many described a loss of personal identity, with some stating they now felt more like a caregiver than a spouse. - Even when ESD was present, carers were not consistently given respite, training, or emotional support.     Lack of adequate carer education and training   - Most carers (12 of 15) reported receiving no structured instruction on how to assist with mobility, personal care, or psychological issues like depression. - The emotional burden of managing mood changes, cognitive issues, and uncertainty was a common theme.     Deficiencies in information provision   - Both patients and carers frequently described the information they received as inadequate, untimely, or overly generic. - Topics such as stroke pathology, prognosis, secondary prevention, and social/financial resources were often not clearly communicated.     Disjointed transition to ongoing rehabilitation services   - Several participants reported a “cliff edge” effect at the conclusion of the six-week ESD period, with long gaps before being connected to other community rehabilitation services. - This resulted in a loss of therapeutic momentum, feelings of abandonment, and functional setbacks.     Differences in expectations between patients and carers   - Patients often focused on physical recovery and regaining independence, while carers were more concerned about coping, logistics, and emotional overload. - This mismatch highlighted the need for dual-focused care planning that addresses both patient and caregiver trajectories.     The home as a therapeutic environment   - Many patients perceived their own home as more conducive to rehabilitation than hospitals, promoting confidence, relevance, and autonomy. - However, the home setting also amplified caregiver responsibilities, especially in cases where professional support was insufficient. | N/A | N/A | N/A | - - Patients were highly satisfied with Early Supported Discharge (ESD), valuing the comfort, privacy, and relevance of home-based rehabilitation.   - Practicing daily tasks in familiar surroundings and receiving individualized attention were seen as major benefits.   - The high intensity of therapy, with up to four sessions daily during the six-week period, was praised.   - Quick initiation of services, often within 24 hours of discharge, boosted motivation and feelings of security.   - Some concerns emerged about poor transition planning from ESD to standard community services, leading to care gaps and reduced continuity. | - - Caregivers had mixed experiences with Early Supported Discharge (ESD), appreciating patient-centered benefits but feeling overlooked themselves.   - Some found home-based therapy reassuring and helpful for patient reintegration.   - Many caregivers reported emotional and physical strain, feeling excluded from care planning and underprepared for caregiving demands.   - Needs for information, emotional support, and respite were often unmet.   - The transition out of ESD was problematic for some, with feelings of abandonment once structured support ended. |
| Glickman et al.[16] | - - **Participants:** 36 clients with stroke or non-stroke diagnoses discharged from Kachere Rehabilitation Centre; 32 guardians also interviewed when available.   - **Sampling:** Consecutive and availability-based.   - **Data collection instruments:**     - WHO Disability Assessment Schedule (WHO DAS 2.0) — measured perceived disability and community reintegration.     - Home Observation Data (HOD) Form — assessed environmental home characteristics (water source, toilets, flooring, access). | Initial rehabilitation took place at Kachere Rehabilitation Centre (inpatient hospital setting).    Post-discharge experiences were evaluated at the clients' homes in suburban Blantyre, in English or Chichewa, according to preference. Clients and guardians were interviewed in separate rooms to avoid bias. | In Year 2, 50.0% of clients perceived moderate to severe levels of disability related to community reintegration (WHO DAS 2.0 scores between 30 and 60).  In Year 1, this percentage was 60.7%.    The main areas of difficulty reported in both years included:   - Taking care of household responsibilities - Participating in day-to-day work or school routines - Joining in community activities - Walking one kilometer     For clients with stroke diagnoses:  Year 1 mean DAS score: 31.2  Year 2 mean DAS score: 28.5    Difference was not statistically significant (p = 0.32)    For clients with non-stroke diagnoses:  Year 1 mean DAS score: 32.1  Year 2 mean DAS score: 29.7    Difference was statistically significant (p = 0.02)    Comparison between clients and guardians (Year 2):  Clients’ mean DAS score: 30.8  Guardians’ mean DAS score: 31.4    Difference was not statistically significant (p = 0.921) | Environmental barriers significantly impacted reintegration. Reported obstacles included:   - Narrow doorways and steps without railings - Pit latrines and water sources located outside the home - Rugged, hilly terrain and lack of transport - Crowded living spaces and small, inaccessible bathrooms     These barriers were confirmed by the Home Observation Data (HOD).    Guardians' perspectives closely aligned with clients’ views.    In Year 2, there was no significant difference between client and guardian WHO DAS 2.0 scores (p = 0.921), indicating strong agreement in perceived reintegration challenges.    The study noted limited or no community rehabilitation services available after discharge from Kachere Rehabilitation Centre, contributing to continued disability and dependence.    Clients using wheelchairs or mobility devices faced greater challenges due to physical environmental limitations and lack of adaptive equipment.    Many clients reported being unable to return to work, often due to a combination of disability, employer inflexibility, and inaccessible work environments.    Guardians were essential to post-discharge care but were unsupported in their need for education and support for guardians, especially in patient-centered models. | Data were collected using two instruments:   - WHO Disability Assessment Schedule (WHO DAS 2.0) – measured perceived disability related to community reintegration. - Home Observation Data (HOD) Form – recorded environmental characteristics of the home (e.g., water sources, toilets, flooring, access features).     Data analysis involved descriptive statistics and non-parametric tests:   - Wilcoxon Signed-Rank Test was used to compare client scores between Year 1 and Year 2. - Mann-Whitney U Test was used to compare client and guardian scores in Year 2. | N/A | p < 0.05 | - - Patients faced persistent challenges with community reintegration after discharge from Kachere Rehabilitation Centre.   - Moderate to severe disabilities were common, especially in managing household tasks, returning to work or school, community participation, and mobility.   - Environmental barriers such as narrow doorways, uneven terrain, lack of assistive devices, and poor transportation access were frequently reported.   - Home conditions, including outdoor toilets and distant water sources, further limited independence.   - The absence of structured post-discharge rehabilitation services led to frustration and difficulties resuming occupational and social roles.   - Guardians provided assistance but could not fully bridge service gaps, resulting in generally low or mixed satisfaction. | - - Guardians played a central role in both inpatient rehabilitation and community reintegration after discharge from Kachere Rehabilitation Centre.   - Their responsibilities extended beyond basic care to supporting daily activities and long-term adjustment.   - Many lived at the hospital alongside the patient during treatment.   - Despite their importance, guardians received little formal support or training.   - By Year 2, guardian perceptions of patient disability closely aligned with patient self-reports, reflecting shared understanding of reintegration challenges. |
| Ashaie et al.[17] | - - **Data collection:** Four time points — admission, discharge, 3 months (T1), and 12 months (T2) post-discharge.   - **Measures used:**     - Community participation (PAR-PRO)     - Positive affect (4 CES-D items)     - Social support (11-item Duke–UNC Functional Social Support Questionnaire)     - Functional independence (3 FIM subscales)   - **Analysis:** Cross-lagged panel network (CLPN) model with regularization and bootstrapping for stability assessment.   - **Additional exploration:** Separate network models for participants with and without aphasia. | Initial rehabilitation took place at hospitals across the U.S    Post-discharge experiences were evaluated at the clients' homes. | Community participation at 3 months post-discharge—especially in religious/spiritual activities—was a strong predictor of motor functional independence and social support at 12 months.   - Religious/spiritual participation → Motor function (β = 0.45) - Socializing outside the home → Social support (β = 0.25)     Positive affect at 3 months was a significant predictor of social support at 12 months.   - Happiness → Social support (β = 0.30)     The strongest overall cross-lagged edge was:   - Social cognition (FIM-Social Cognition) → Motor function (FIM-Motor) (β = 0.56)     In the aphasia group, the strongest predictor was:   - Happiness → Motor function (β = –1.67)     This was not observed in the no-aphasia group, where the strongest predictor was:   - FIM-Social Cognition → FIM-Motor (β = 0.89)     The participation network differed substantially between individuals with and without aphasia (only 44% shared edges), indicating that language impairment alters recovery dynamics. | Participation in religious/spiritual activities had a high “out-expected influence” (out-EI), meaning it influenced several other recovery domains, especially motor function.  Feeling happy at 3 months post-discharge was a significant predictor of later social support, indicating that emotional state may enhance help-seeking behaviors and relational engagement.  This supports the idea that mental health directly contributes to social recovery.    Differences in recovery pathways between aphasia and non-aphasia groups  In the aphasia group, positive affect played a stronger role in predicting outcomes, suggesting that emotional well-being may be especially critical when communication is impaired.    Functional abilities in social cognition (e.g., interpreting social cues, managing relationships) strongly predicted later motor functional independence, suggesting that cognitive-linguistic function is closely tied to physical recovery.    Community participation is not only an outcome but also a predictor | - - PAR-PRO: Home and Community Participation Questionnaire     - Used to assess community participation across four domains:       - Socializing inside the home       - Socializing outside the home       - Recreation and leisure       - Religious/spiritual activities     - Rated on a 5-point Likert scale (higher scores = more frequent participation)   - CES-D (Center for Epidemiologic Studies – Depression Scale) – positive affect items only     - Four positively worded items used to assess positive emotions, such as:       - "I felt hopeful about the future"       - "I was happy"       - "I enjoyed life"       - Rated on a 5-point Likert scale (higher scores = more frequent positive affect)   - Duke–UNC Functional Social Support Questionnaire (11-item version)     - Measured perceived social support     - Includes items like “I get help around the house”     - Rated on a 5-point Likert scale (higher scores = more support)   - Functional Independence Measure (FIM) – 3 subscales     - Motor     - Social cognition     - Communication     - Rated on a 7-point Likert scale (higher scores = greater independence) | Outcomes were assessed at 3 months (T1) and 12 months (T2) after discharge from inpatient rehabilitation | While p-values were not reported for each edge, the use of β coefficients in a regularized CLPN model and bootstrapped validation provides a robust representation of effect strength and direction of relationships between variables across time (from 3 to 12 months post-discharge).    Examples of statistically strong associations:  FIM-Social Cognition → FIM-Motor: β = 0.56 (strongest edge in the overall network)  Religious/Spiritual Activities → FIM-Motor: β = 0.45  Happiness → Social Support: β = 0.30  Socializing Outside the Home → Social Support: β = 0.25    In the aphasia group, the strongest predictive association was:  Happiness → FIM-Motor: β = –1.67 (noted as unusually strong and specific to this subgroup)    Stability and accuracy testing were performed using bootstrapped confidence intervals and correlation stability (CS) coefficients:  CS coefficients for centrality indices (in-EI and out-EI) were low (0.21 and 0.13), indicating limited stability of those specific measures. | N/A | N/A |
| Sinclair et al.[18] | - - **Design:** Two-phase study on service provision and gaps.   - **Phase 1:** Focus group with six NHS/social care providers to map existing services; analysis of formal service data, websites, and brochures.   - **Phase 2:** Qualitative exploration of service gaps using semi-structured interviews, observations, documentary analysis, and a large stakeholder engagement event.   - **Participants:** 49 stakeholders (service users, providers, commissioners, voluntary sector).   - **Analysis:** Interviews analyzed using SSM “CATWOE” framework; thematic analysis conducted with NVivo; multiple coders used for validation. | Qualitative investigation into gaps in service provision using a mixed-methods approach, including formal and informal semi-structured interviews, observations, documentary analysis, and a large stakeholder engagement event. Interviews were recorded, transcribed, and analyzed using the SSM “CATWOE” framework to explore stakeholders' worldviews. | There was no sanctioned or structured pathway for stroke survivors seeking to return to work; existing VR support was fragmented, inequitable, and poorly coordinated across sectors.    Communication between services was largely informal and inconsistent, relying on individual clinician initiative rather than formal protocols.    Mild stroke survivors—particularly those with hidden cognitive impairments—were often underserved and described as “falling through the net.”    The timing of VR interventions was highly complex: while early intervention was viewed as ideal, many survivors were not emotionally or functionally ready to discuss returning to work immediately after discharge. A more flexible and responsive service model was recommended.    Service providers lacked stroke-specific VR training, and many non-health stakeholders lacked understanding of stroke-related disabilities, especially cognitive or “invisible” impairments.    Vocational rehabilitation was not widely perceived as a core responsibility of the health sector. Many commissioners felt VR should be funded or provided by non-health sectors, such as the Department for Work and Pensions. | Mild stroke survivors were routinely overlooked    Poor inter-sector communication and unclear service roles   - Communication between health, social care, and employment sectors was fragmented. - Referrals were mostly unidirectional, with little ability to re-enter health services once discharged. - Service providers and GPs often lacked awareness of each other’s roles or available VR services.     NHS staff lacked formal training in vocational rehabilitation and often used a "common sense" approach.    Fear of legal or ethical implications (e.g., employer disclosure) discouraged service provider involvement with workplaces to manage stroke-specific needs    VR not seen as “core business” by commissioners   - Commissioners lacked awareness of the evidence base supporting VR and requested clearer financial justifications (e.g., cost-savings, tax contributions).     While early intervention prevents job loss, some survivors were not psychologically or physically ready soon after stroke.    A “dip-in, dip-out” model was suggested to accommodate varying recovery trajectories.    Commissioners preferred generic over condition-specific services   - Stroke-specific VR was seen as harder to justify or fund. Commissioners favored broader vocational services serving various disabilities to improve scalability and cost-effectiveness. | N/A | N/A | N/A | - - Stroke survivors felt abandoned after hospital discharge, with little structured support for returning to work.   - Many described emotional distress, including depression and, in one case, a suicide attempt linked to work-related hopelessness.   - Hidden cognitive impairments made work reintegration difficult, often overlooked by healthcare services.   - Participants highlighted the absence of clear vocational pathways or employment-focused rehabilitation.   - Some returned to work prematurely due to financial or emotional pressures, leading to feelings of overwhelm.   - Survivors faced poor coordination between healthcare and employment services, often needing to advocate for themselves. | N/A |
| Askew et al.[19] | - - **Design:** Prospective longitudinal cohort study at a large urban comprehensive stroke center (USA).   - **Outcome measures:**     - Disability: Modified Rankin Scale (mRS) and Barthel Index (BI) at 30 days via telephone interview.     - HRQoL: Four Neuro-QoL domains (executive function, cognitive concerns, upper extremity dexterity, lower extremity mobility).   - **Analysis:** Linear and logistic regression models, stratified by stroke severity (NIHSS ≤5 vs. NIHSS >5).   - **Focus:** Impact of timing of therapy consults and treatments on 30-day functional and quality-of-life outcomes. | In-hospital setting:  Clinical and demographic data, including stroke severity (NIHSS), comorbidities, and timing of therapy consults and treatments, were collected during the acute hospitalization from the hospital’s electronic health records and clinical documentation.    Telephone setting (follow-up):  At 30 days post-discharge, outcomes were assessed via standardized telephone interviews, including:  Modified Rankin Scale (mRS)  Barthel Index (BI)  Neuro-QoL patient-reported outcome measures (executive function, cognitive concerns, upper/lower extremity function) | **For patients with mild strokes (NIHSS ≤5):**   - Longer time to therapy consult was associated with:   - Worse Barthel Index scores (OR = 0.818, *p* = 0.008)   - Lower executive function (β = –0.865, *p* = 0.001)   - Lower general cognitive function (β = –0.609, *p* = 0.009)   **For patients with moderate-to-severe strokes (NIHSS >5):**   - Longer time to therapy treatment was associated with:   - Greater disability (mRS ≥2) (OR = 1.151, *p* = 0.039)   - Lower lower extremity mobility (β = –0.591, *p* = 0.046)   **General findings across the full sample:**   - Each additional day of delay in initiating therapy consults was linked to worse disability and HRQoL outcomes at 30 days. - A 7-day delay in therapy consults for mild strokes led to a drop of ~4 points in cognitive function and ~6 points in executive function T-scores (about ½ SD, considered clinically meaningful). | Differential effect of therapy type based on stroke severity   - The study found that therapy consults had a greater impact on outcomes in mild stroke patients, while therapy treatments (i.e., actual rehabilitative sessions) had a stronger influence in patients with moderate-to-severe stroke. - This suggests a need to tailor early rehabilitation strategy based on clinical severity.   PROs (Neuro-QoL) were more sensitive in identifying cognitive and physical deficits in mild stroke cases compared to clinician-rated tools like the mRS.    No patients received therapy within 24 hours of admission   - Unlike the AVERT trial, this real-world cohort began therapy with a median delay of 2 days, suggesting that early intervention windows are often missed, despite guideline recommendations.   Delays of just 7 days in initiating therapy were associated with clinically important declines (≥½ standard deviation) in mobility and cognition scores, reinforcing that even short delays can significantly affect recovery.    Predictors of delayed therapy included stroke type and length of stay   - Patients with TIA, prior stroke, or longer ICU/non-ICU stays experienced longer delays in receiving therapy consults and treatments. - This suggests that system-level or triage-related delays may disproportionately affect certain clinical subgroups.     Therapist prioritization may be influenced by perceived discharge timing   - The authors suggest that therapists might prioritize patients expected to be discharged soon, aiming to meet quality benchmarks, potentially delaying care for those staying longer. | - - Modified Rankin Scale (mRS)   Used to assess overall functional outcome at 30 days     - - Barthel Index (BI)   Used to assess physical disability and daily function     - - Neuro-QoL (Quality of Life in Neurological Disorders) Patient-Reported Outcome Measures   Four short forms were used to assess health-related quality of life across domains:   - - Executive Function (EF)   - General Cognitive Concerns (GCC)   - Upper Extremity Dexterity (UED)   - Lower Extremity Mobility (LEM)   - Each scored with a mean of 50 and SD of 10 (U.S. general population norm); higher scores reflect more of the domain being measured (e.g., greater mobility or greater cognitive concern) | Outcomes were assessed at 30 days post-discharge | **For mild stroke (NIHSS ≤ 5):**  Longer time to therapy consult predicted:   - Worse Barthel Index scores → OR = 0.818, p = 0.008 - Lower Executive Function T-scores → β = –0.865, p = 0.001 - Lower General Cognitive Concerns T-scores → β = –0.609, p = 0.009     **For moderate-to-severe stroke (NIHSS > 5):**  Longer time to therapy treatment predicted:   - Higher odds of moderate/severe disability (mRS ≥ 2) → OR = 1.151, p = 0.039 - Lower Lower Extremity Mobility T-scores → β = –0.591, p = 0.046 | N/A | N/A |
| Mattioli et al.[20] | - - **Design:** Randomized study with 12 adult patients (first-ever left MCA ischemic stroke); 10 healthy controls.   - **Groups:** Rehabilitation group (n=6, daily language therapy) vs. non-rehabilitation group (n=6, standard care).   - **Intervention:** Language therapy 1 hour/day, 5 days/week, for 2 weeks starting ~2.2 days post-stroke.   - **Assessments:** Neuropsychological testing (Aachen Aphasia Test) and fMRI at baseline (T1), two weeks (T2), and six months (T3).   - **Analysis:** fMRI activation patterns in language areas (inferior frontal gyrus, superior temporal gyrus); ROI and time-by-treatment interaction analyses.   - **Blinding:** Speech therapist conducting assessments was blinded to group assignment. | Early, intensive aphasia language therapy, started on average 2.2 days post-stroke, administered 1 hour/day, 5 days/week for 2 weeks    Conducted in inpatient acute setting with assessments using:   - Neuropsychological testing (Aachen Aphasia Test) - Functional MRI (fMRI) during an auditory comprehension task     Evaluations took place at three time points:   - T1 = ~2 days post-stroke - T2 = ~16 days post-stroke - T3 = ~190 days (6 months) post-stroke | **At baseline (T1)** : no significant differences were observed between groups in language test performance or brain activation.    **At T2 (≈16 days post-stroke)**:  Rehabilitated patients showed significantly greater improvement in:   - Naming (mean score: 110/120 vs. 80/120; p = 0.01) - Written language (mean score: 84.5/90 vs. 48.7/90; p = 0.02) - fMRI showed increased activation in the left inferior frontal gyrus (Broca area) in the Rehab group only. - A significant treatment × time interaction was found in the left IFG at T2 (F = 10.2; p = 0.009).     **At T3 (≈6 months post-stroke)**:   - The Rehab group continued to outperform the NonRehab group: - Naming (113/120 vs. 98/120; p = 0.004) - Written language (85.5/90 vs. 71/90; p = 0.03) - Both groups showed increased left hemisphere activation over time, but Rehab patients maintained higher activation in LH language areas, especially the left IFG.     **Correlation analysis**:   - In the Rehab group, increased activation in the left IFG at T2 strongly correlated with improvement in naming (r = 0.957; p < 0.003). - In the NonRehab group, improvement in naming was correlated instead with right IFG activation (r = 0.821; p < 0.015), suggesting different neural mechanisms of recovery. | Different neural recovery pathways in treated vs. untreated patients   - Treated (Rehab) patients showed early and predominant activation of the left inferior frontal gyrus (IFG), especially Broca’s area. - Untreated (NRehab) patients showed a recovery pattern involving right hemisphere language-related areas, particularly the right IFG, which is often associated with spontaneous, less efficient recovery.      - Sustained left hemisphere (especially the IFG) recruitment in the Rehab group for at the 6-month follow-up, suggesting durable neuroplastic effects of early rehabilitation.     The study suggests that right IFG activation in NRehab patients may reflect a less optimal or maladaptive recovery route, supporting the hypothesis that left hemisphere recruitment is a better prognostic marker for functional recovery.    Although the main improvements were seen in naming and written language, fMRI showed broader activation changes across temporal and parietal regions, indicating that rehabilitation might affect brain regions not directly linked to measured behavioral tasks.    The strong correlation between naming improvement and fMRI activation in the left IFG reinforces the concept that targeted language therapy drives task-relevant cortical reorganization in acute stroke.    Therapy may accelerate the timeline of recovery-related cortical reorganization  Compared to untreated patients, those receiving early therapy showed faster and more left-lateralized cortical changes, suggesting that therapy can shift the temporal dynamics of neuroplastic recovery. | Aachen Aphasia Test (AAT)  A standardized neuropsychological battery used to assess language function    Administered at three time points:   - T1 (~2 days post-stroke) - T2 (~16 days post-stroke) - T3 (~6 months post-stroke)     Subtests included:   - Naming - Repetition - Reading and writing - Oral and written comprehension - Spontaneous speech - Token test     Scores were based on the number of correct responses, with additional semiquantitative scores for spontaneous speech dimensions    Functional MRI (fMRI) with auditory comprehension task to assess brain activation patterns related to language processing. Participants completed an event-related auditory comprehension task during scanning. Functional changes were analyzed using:   - Whole-brain activation maps - Region-of-interest (ROI) analysis - Longitudinal comparisons (T1 vs T2 vs T3) - Correlations with AAT performance (e.g., naming) | Short-term: at 2 weeks (T2)    Long-term: at 6 months (T3) | Behavioral outcomes (Aachen Aphasia Test):  At T2 (16 days post-stroke):   - Naming: Rehab group scored significantly higher than NRehab (110/120 vs. 80/120; p = 0.01) - Written language: Rehab group also significantly better (84.5/90 vs. 48.7/90; p = 0.02)     At T3 (6 months post-stroke):   - Naming: Rehab group still outperformed NRehab (113/120 vs. 98/120; p = 0.004) - Written language: Continued difference (85.5/90 vs. 71/90; p = 0.03)     Neuroimaging outcomes (fMRI):   - Left inferior frontal gyrus (IFG) showed a treatment × time interaction at T2: - ANOVA result: F = 10.2; p = 0.009 - Post hoc test: Significantly greater activation in the Rehab group (p < 0.05)     Correlation analyses:  Rehab group:   - Change in left IFG activation (T2–T1) strongly correlated with naming improvement - Correlation coefficient: r = 0.957; p < 0.003     NRehab group:   - Naming improvement correlated with right IFG activation - Correlation coefficient: r = 0.821; p < 0.015 | At the second follow-up (T2), the authors report that rehabilitated patients and their relatives appeared satisfied with the improvements in language functions. However, no structured interviews, questionnaires, or direct reports of satisfaction were included in the study design. | N/A |
| Martin et al.[21] | - - **Participants:** 31 adults post-stroke who had attempted or considered returning to work.   - **Data collection:** Semi-structured interviews (40–90 minutes), conducted by an occupational therapist with qualitative research training.   - **Focus:** Experiences of stroke, work, recovery, and identity.   - **Analysis:** Inductive reflexive thematic analysis (Braun and Clarke's method), with iterative coding and team refinement.   - **Analytical framework:** Realist principles to explore how, why, and in what contexts return-to-work experiences occurred. | Interviews were conducted via online (n=17), telephone (n=1), in participants' homes (n=5), or at the researcher’s workplace (n=8)    Data were gathered through semi-structured interviews | The study identified four key themes that reflect how stroke survivors made sense of their return-to-work journeys and changing occupational identities:   1. “It’s not just work”: Stroke disrupts occupational identity   Even those who physically returned to work struggled with confidence, purpose, and perceived value.   1. “How will I get back?”: Pathways to work were inconsistent and unclear 2. “Fighting against the current”: Systems and services weren’t built for stroke survivors especially those with invisible impairments (e.g., fatigue, cognitive difficulties).   They had to self-advocate, often repeatedly, to access services or be taken seriously.   1. “Let me do me”: Identity reconstruction through supported autonomy   The ability to experiment with new or adapted roles helped many participants rebuild confidence and find new meaning in work.  Supportive professionals who listened, adjusted expectations, and created space for self-reflection were critical to recovery.  Early opportunities to explore identity change, rather than just focusing on job tasks or timelines, were highly valued. | Early framing and introduction of return-to-work influenced recovery trajectory  Invisible impairments created unique barriers  Participants with fatigue, cognitive dysfunction, or communication difficulties often felt misunderstood or judged as “lazy” or “fully recovered.”    Health system structure favored physical recovery over vocational identity    Self-advocacy was a survival strategy—but often exhausting  This burden was especially high for those without strong family, financial, or institutional support.    Vocational rehabilitation was inconsistently accessed and unevenly impactful  Less than half of the participants had received formal vocational rehabilitation.    Participants emphasized that early identity-focused VR (not just task-based assessments) would have better supported long-term success.    Peer and community networks (volunteering, advocacy, or peer mentoring) helped rebuild occupational confidence | N/A | N/A | N/A | - - Return-to-work experiences were often confusing and frustrating, with little coordinated support.   - Services were seen as overly focused on physical recovery, neglecting occupational identity and long-term goals.   - Early discussions about work were frequently absent or felt disempowering to participants.   - Quality of vocational rehabilitation varied widely; empathetic, flexible support was highly valued.   - Many faced isolation and had to self-advocate, particularly when dealing with invisible impairments like fatigue or cognitive issues.   - Satisfaction depended heavily on feeling recognized and supported as whole individuals rather than just patients. | N/A |
| Lin et al.[22] | - - **Design:** Randomized controlled study with 62 stroke patients (intervention group n=31; control group n=31).   - **Intervention:** Routine rehabilitation (50 min/session, 2×/week, 8 weeks) + additional 10-minute social support and health education sessions (intervention group only).   - **Support content:** Emotional encouragement, home rehabilitation advice, leisure activity promotion, anxiety coping strategies.   - **Outcomes measured:** Depression (CES-D10) at weeks 1, 2, 4, 8, and 12 (follow-up).   - **Analysis:** Mann–Whitney U, Wilcoxon signed-rank, McNemar tests, and GEE models (SPSS).   - **Retention:** No participant dropout. | In-person at a rehabilitation department where therapy was conducted twice per week (50 minutes per session) for 8 weeks. In addition, the intervention group received an extra 10-minute session of social support and health education, also twice per week | In the intervention group, depression scores significantly decreased over time:   - Week 4: 6.03 ± 4.61 - Week 8: 5.29 ± 4.33 - Week 12 (follow-up): 6.00 ± 5.05     All changes from baseline were statistically significant (p < 0.05)    In the control group, depression scores showed no statistically significant change from baseline at any time point.    After 8 weeks of intervention, the difference in depression scores between the intervention and control groups was statistically significant (p = 0.02 at week 8).    Generalized estimating equation (GEE) analysis confirmed the effect:   - Depression scores in the intervention group were 1.24 points lower than in the control group (p < 0.001) - Week 8 scores were significantly lower than week 2 scores (p = 0.03) | - - Depression scores were negatively correlated with leisure satisfaction and presence of caregivers   - Depression scores were positively correlated with stroke duration and economic difficulty   - Pain was associated with higher depression scores   - Baseline functional status did not predict depression improvement | Center for Epidemiologic Studies Depression Scale – Short Form (CES-D10):  A 10-item version used to assess depressive symptoms in chronic stroke patients.    Total score range: 0 to 30 (higher scores indicate more severe depression).    Administered at 5 time points:   - Week 1 (baseline) - Week 2 - Week 4 - Week 8 - Week 12 (follow-up)     Cutoff thresholds of 8–10 were used to identify depressive tendency based on prior Taiwanese validation studies. | Depression scores were followed and reassessed at:    Baseline (week 1)    2nd week    4th week    8th week    Follow-up at 12th week | Within-group changes (intervention group):    Week 1 vs Week 4:   - Depression score significantly decreased (p = .027)     Week 1 vs Week 8:   - Significant improvement (p = .002)     Week 1 vs Week 12 (follow-up):   - Significant difference maintained (p = .021)     Between-group differences (intervention vs control):   - At Week 8, the difference between groups was statistically significant (p = .02)     At other time points (Weeks 2, 4, and 12), differences were not statistically significant    Generalized Estimating Equations (GEE) model results:  The intervention group had a 1.24-point lower depression score compared to the control group (p < .001)    Week 8 vs Week 2 within the intervention group showed a significant reduction (p = .03) | N/A | N/A |
| Liu-Ambrose et al.[23] | - - **Design:** Randomized study with 28 chronic stroke survivors (intervention group vs. delayed-intervention control group).   - **Eligibility:** Age >55, ≥1 year post-stroke, community-dwelling, able to walk >10 meters, completed formal rehab.   - **Intervention:** 6-month program — twice-weekly FAME-based exercise sessions + once-weekly recreational activities (social, cognitive engagement).   - **Control group:** Usual care for first 6 months, then received the same intervention.   - **Delivery:** In-person, small group sessions at community facilities.   - **Assessments:** Cognitive tests (Trail Making, Stroop) and physical performance measures every 3 months over 15 months; assessors blinded. | Six-month structured community-based program combining:    2x/week exercise training (resistance, aerobic, and balance; based on FAME protocol)    1x/week recreational and leisure activities (planning, strategy, social interaction)    Assessments conducted in person in community facilitiesevery 3 months over 15 months | The intervention group (INT) showed a statistically significant improvement in executive function compared to the delayed-intervention group (D-INT) at 6 months:   - TMT B–A score:   - INT group showed greater improvement in completion time compared to D-INT   - Effect size = 0.60 (moderate effect), p = 0.04      - Stroop Interference score:   - Also improved in the INT group compared to D-INT   - Effect size = 0.65, p = 0.03     Cognitive gains were maintained at 12-month follow-up, suggesting sustained benefit beyond the active intervention phase.    The delayed-intervention group showed similar improvements after they began the program, confirming the replicability of the effect. | The study demonstrated that a structured community-based program combining exercise and recreation was feasible and well-tolerated by chronic stroke survivors.    Adherence rates were high, and no adverse events were reported, supporting the safety and acceptability of delivering this type of intervention outside clinical settings.    Social and motivational benefits from recreational sessions which are indirectly beneficial to cognitive and psychological recovery.    The group format may have supported motivation and accountability.    Executive function benefits were independent of baseline mobility status  Sustained executive function gains beyond active intervention  The D-INT group, once they received the same intervention, also demonstrated similar improvements in executive function, reinforcing the intervention’s reliability across time and participants.  The study highlighted the value of integrating physical and cognitive-social activities in accessible, non-clinical environments to promote neurocognitive health in chronic stroke populations. | TMT B–A was used as the primary measure of cognitive flexibility (an executive function domain).  Measures the ability to alternate between sequences (numbers and letters) under timed conditions.    Lower scores (faster time) indicate better performance.    Stroop Color-Word Interference Test was used to assess inhibitory control, another core aspect of executive function.  Participants had to inhibit automatic reading responses to correctly identify font colors.  The interference score (reaction time difference between congruent and incongruent tasks) was analyzed.  Lower scores indicate better inhibitory control.    Montreal Cognitive Assessment (MoCA) was used at baseline only to assess general cognitive status. | Participants were followed for six months during the intervention, plus an additional six-month follow-up period, totaling 15 months of observation. | Trail Making Test (TMT B–A):   - Effect size: 0.60 - p-value: 0.04 - Interpretation: Significant improvement in cognitive flexibility (moderate effect)     Stroop Interference Score:   - Effect size: 0.65 - p-value: 0.03 - Interpretation: Significant improvement in inhibitory control (moderate effect) | N/A | N/A |
| O’Callaghan et al.[24] | - - **Aim:** Explore experiences and preferences of stroke survivors and caregivers regarding support during hospital-to-home transition.   - **Participants:** Adults >18 years, stroke within past 6 months, discharged home (directly, via inpatient rehab, or ESD).   - **Data collection:** Semi-structured interviews (1–6 months post-discharge) conducted at home, by phone, or via teleconference.   - **Interview focus:** Care transition experiences, support service usefulness and timing, unmet needs, improvement suggestions.   - **Analysis:** Reflexive thematic analysis (Braun and Clarke); inductive coding by first author, team discussions to refine themes. | Interviews were conducted:    At participants' homes,    Via telephone, or    Via telecommunication platform (e.g., Microsoft Teams)    Conducted by a trained researcher using a semi-structured interview guide | Four key themes were identified:   1. “Stepping into the unknown” – the uncertainty of leaving hospital   They reported limited involvement in discharge planning and a lack of clarity around available supports.   1. “We need support to support them” – the weight of responsibility for caregivers 2. “You’re just left on your own” – fragmented and insufficient community supports 3. “Support that works for us” – what people value in post-discharge care   Participants emphasized the importance of clear communication, continuity, and person-centered support tailored to their unique needs and readiness.  Preferred services included timely follow-up, in-home rehabilitation, emotional reassurance, and a single point of contact to help navigate the system. | Discharge readiness and patient empowerment were lacking    Services were more effective when they were timely, individualized, and responsive to changing needs, rather than standardized or bureaucratic. | N/A | N/A | N/A | - - The transition from hospital to home was often experienced as uncertain and emotionally difficult.   - Many felt unprepared for discharge, with limited involvement in planning and unclear post-hospital information.   - While some benefited from ESD or organized follow-up, most described fragmented care, long wait times, and poor communication between services.   - A common frustration was not knowing who to contact for rehabilitation or emotional support.   - The lack of a single, consistent point of contact intensified feelings of being left alone.   - Positive experiences occurred when services were timely, continuous, and tailored to individual needs. | - - Caregivers experienced the transition from hospital to home as highly stressful, often assuming major responsibilities without adequate support.   - Many felt excluded from discharge planning despite their central caregiving role.   - At home, caregivers had to act as coordinators, advocates, and therapists without formal training.   - Anxiety, frustration, and exhaustion were common, especially when facing unclear service pathways and rehabilitation delays.   - Emotional support for caregivers was largely absent, leaving many feeling overwhelmed and isolated.   - Caregivers stressed that their ability to support survivors depended heavily on service quality and timeliness.   - They called for better involvement in planning, clearer information, and a central contact person for guidance. |
| May et al.[25] | - - **Aim:** Explore experiences of stroke survivors attending a peer-led, community-based support group (BLAST).   - **Participants:** 11 active BLAST members; stroke survivors.   - **Data collection:** Semi-structured individual interviews (30–60 minutes) conducted virtually (Zoom or telephone).   - **Intervention setting:** Weekly peer-support meetings focused on social, recreational, and mutual support activities.   - **Interview guide:** Developed collaboratively with individuals with lived stroke experience to ensure accessibility. | Interviews were conducted virtually, either by Zoom or phone, due to COVID-19 restrictions    Duration: 30 to 60 minutes    Interviewers followed a semi-structured interview guide | The researchers identified four key themes based on participant interviews:   - “They understand me”: Shared understanding fosters connection   Shared stroke experiences enabled deep empathy, particularly important for those with aphasia or hidden disabilities.     - “They’re my people”: Belonging and identity reconstruction   Participants described the group as “like a family,” offering consistent companionship, routine, and a valued social role.     - “We help each other”: Mutual support and reciprocal empowerment   Helping others was seen as empowering and gave participants a sense of purpose and usefulness.     - “The group saved me”: Emotional survival and long-term wellbeing | Peer support filled long-term service gaps of the formal rehabilitation services which often ended abruptly, leaving them without support for emotional, social, or identity-related recovery.  Peer-led structure enhanced accessibility and relevance  Members felt empowered by the shared leadership and lack of hierarchical dynamics, contributing to a stronger sense of ownership and participation.    Aphasia inclusion was especially valuable  Participants with expressive aphasia described the group as one of the only places where they felt truly understood and heard.  Communication was naturally adapted within the group, creating a safe space for expression even without clinical speech support.    Social routine and structure were protective for mental health  For some, this routine was described as life-saving, especially for those who had struggled with depression or isolation. | N/A | N/A | N/A | - - The BLAST peer-led support group was described as a crucial lifeline after formal rehabilitation ended.   - Participants found emotional safety, belonging, and deep empathy within the group, especially important for those with aphasia or invisible disabilities.   - The non-clinical, flexible structure fostered mutual support, meaningful relationships, and identity rebuilding.   - Many highlighted that the group helped combat isolation and gave them renewed purpose.   - Family members often lacked full understanding, making peer support even more vital.   - Helping others within the group was experienced as empowering and healing.   - Although not offering formal therapy, the group was seen as essential for emotional well-being and life satisfaction. | N/A |
| Koositamongkol et al.[26] | - - **Aim:** Identify factors influencing functional recovery after acute ischemic stroke.   - **Participants:** 141 adults with first-ever ischemic stroke (confirmed by CT/MRI).   - **Data collection:** Hospital-based; assessments at day 3, day 7, and discharge.   - **Measures:** NIHSS-T, Modified Barthel Index–Thai (BI-T), Charlson Comorbidity Index (CCI-T), CES-D-T for depressive symptoms, and a 24-point stroke care quality score.   - **Analysis:** Patients stratified by stroke severity; univariate and multivariate logistic regression to identify predictors of recovery. | In-patient setting | - 1. Neurological Recovery:   rt-PA (thrombolysis) was the strongest and most consistent predictor of neurological recovery:   - - Day 3: OR = 7.085, p < .001   - Day 7: OR = 4.714, p = .004   - Discharge: OR = 4.714, p = .004     Stroke severity at admission also predicted recovery:   - - Mild/moderate stroke more likely to recover than severe strokes   - p < .05 across all time points      - 1. ADL Functional Recovery:   rt-PA was also significantly associated with higher ADL recovery:   - - Day 3: OR = 4.086, p = .033   - Discharge: OR = 5.408, p = .002     Higher quality acute stroke care services were positively associated with greater ADL improvement  Patients who received “good” service scores (≥20/24 on the acute care service scale) had better outcomes not only in ADL function but also in terms of reduced depressive symptoms.     - 1. Depression at Discharge (Psychological Recovery):   Although stroke severity was not directly correlated with depression, the quality of services received was inversely associated with depression, highlighting the emotional impact of care experience.  Quality of acute stroke care services was significantly associated with lower rates of depressive symptoms:   - - OR = 1.312, p = .049     16.3% of patients were diagnosed with significant depressive symptoms (CES-D ≥ 23) and 36.2% had moderate depressive scores (CES-D-T ≥ 16).    No significant association was found between depression and comorbidities or demographic characteristics | Only 14.2% of patients received rt-PA, even though it was the strongest predictor of neurological and functional recovery.  This finding suggests a gap between clinical effectiveness and real-world application, possibly due to delayed presentation or systemic barriers.    Most improvements occurred within the first week of hospitalization, emphasizing the importance of early interventions and acute-phase intensity. | NIHSS-T (National Institutes of Health Stroke Scale – Thai version):  Used to assess neurological function  Recovery defined as a reduction of ≥4 points    BI-T (Modified Barthel Index – Thai version):  Used to assess ADL function (Activities of Daily Living)  Recovery calculated using a potential improvement formula    CES-D-T (Center for Epidemiologic Studies Depression Scale – Thai version):  Used to assess depressive symptoms at discharge  Cutoffs: ≥16 = depressive symptoms; ≥23 = significant depression    CCI-T (Charlson Comorbidity Index – Thai version):  Used to score co-morbidities    Measurement of Acute Stroke Care Services Received (custom form):  Scored 0–24; categorized into poor, fair, or good service levels | N/A | Neurological function recovery:  rt-PA (thrombolysis) was a strong predictor at:   - Day 3: OR = 7.085, p = .000* - Day 7: OR = 4.714, p = .004 - Day of discharge: OR = 4.714, p = .004     ADL recovery:  rt-PA was significant at:   - Day 3: OR = 4.086, p = *.033 - Day of discharge: OR = 5.408, p = .002     Psychological function (depression):  Acute stroke care service score predicted reduced depressive symptoms: OR = 1.312, p = .049*    Multivariate binary logistic regression confirmed these associations. | N/A | N/A |
| Lindley et al. (The ATTEND Collaborative Group)[27] | - - **Design:** Multicenter randomized controlled trial (14 hospitals, India); PROBE design.   - **Participants:** Adults with stroke (ischemic or hemorrhagic) and a willing caregiver.   - **Intervention:** Family-led rehabilitation program (hospital training + up to 6 home visits + 2 follow-up calls over 2 months), guided by a standardized manual.   - **Control:** Usual care.   - **Outcome measures:**     - Primary: Death or dependency at 6 months (mRS 3–6).     - Secondary: Barthel Index (ADL), Nottingham EADL, WHOQOL-BREF, EQ-5D, HADS, Caregiver Burden Scale, mortality, readmissions, serious adverse events.   - **Data collection:** Home visits, telephone follow-ups, medical record reviews.   - **Analysis:** Intention-to-treat; blinded outcome assessors. | In-hospital caregiver training:   - Delivered over approximately 3 days, with 1 hour of training per day - Conducted before the patient was discharged from the hospital - Provided by trained stroke coordinators using a manualised program     Focused on:   - Task-specific training (e.g., feeding, walking, dressing, transfers) - Communication strategies for aphasia - Home safety, positioning, and use of assistive devices - Psychosocial support for both patient and caregiver - Goal-setting and tracking progress     Post-discharge home visits:   - Up to 6 home visits by the stroke coordinator over the first 2 months - Focused on reinforcing skills, addressing barriers, and updating rehabilitation plans - Delivered at the patient’s home to adapt training to the living environment     Telephone support:   - Up to 2 follow-up calls during the same 2-month period - Used to provide additional guidance, motivation, and answer caregiver questions | Primary Outcome (Death or dependency at 6 months):  No significant difference between groups:  Intervention: 47%  Control: 47%    Odds Ratio (OR): 0.98    95% CI: 0.78–1.23    p = 0.87    Secondary Outcomes:  No significant differences in    Subgroup analysis:  A significant interaction by sex was noted (p = 0.037):  Men in the intervention group showed better outcomes than men in the control group  This effect was not seen in women | Gender-specific differences in response to the intervention  A significant interaction by sex was found in subgroup analyses (p = 0.037), with male patients in the intervention group experiencing better outcomes (lower death or dependency) than males in the control group.    Female patients, however, did not benefit from the intervention, suggesting the need to consider gender dynamics and caregiving roles in family-led models.    The intervention was well accepted by participants, had no safety concerns, and was delivered consistently across 14 hospitals, confirming that such a model is scalable and safe in low-resource settings.    Despite assuming a more active rehabilitation role, caregivers in the intervention group did not report higher burden (measured by the Caregiver Burden Scale) than those in the control group.  This suggests that the structured training and support may have buffered stress, even with increased responsibility.    The intervention had no significant impact on anxiety, depression, or quality of life in either patients or caregivers, based on HADS, WHOQOL-BREF, or EQ-5D scores. This indicates that while the intervention supported functional recovery tasks, emotional and psychological support needs remained unaddressed.    Participants came from diverse income and educational backgrounds, and the intervention was delivered effectively across urban and rural hospitals, suggesting that family-led rehabilitation is practically implementable across social strata in India. | Modified Rankin Scale (mRS) — primary outcome (death or dependency defined as mRS 3–6)    Barthel Index — ADL performance    Nottingham Extended ADL Scale (EADL)    WHOQOL-BREF — quality of life    EQ-5D — health status    Hospital Anxiety and Depression Scale (HADS) — for both patients and caregivers    Caregiver Burden Scale | 3 and 6 months post-discharge | Primary outcome (death or dependency at 6 months):  47% in both intervention and control groups  OR = 0.98; 95% CI: 0.78–1.23; p = 0.87 → not significant    Mortality: 12% (intervention) vs 14% (control), p = 0.27  Subgroup analysis:    Significant interaction by sex (p = 0.037): men in intervention group had better outcomes, not seen in women | N/A | N/A |
| Tramonti et al.[28] | - - **Design:** Longitudinal observational study at Azienda Ospedaliero-Universitaria Pisana (Pisa, Italy).   - **Participants:** 29 stroke survivors (ischemic or hemorrhagic); excluded if global aphasia or MMSE <24.   - **Intervention:** Standard individualized neurorehabilitation (physical, psychological, social interventions).   - **Data collection:** At admission (T1) and discharge (T2) during inpatient rehabilitation.   - **Measures:** Barthel Index, SF-36, SEIQoL-DW, HADS, COPE questionnaire, MSPSS.   - **Analysis:** Paired t-tests (pre-post changes) and Pearson correlations (relationships between psychosocial variables and outcomes).   - **Follow-up:** None post-discharge; study limited to in-hospital effects. | In-patient setting with direct assessment at admission (T1) and at discharge (T2) | - - Functional status (Barthel Index):     - Patients showed a significant improvement in functional independence from admission to discharge.     - p < 0.01 — confirming effectiveness of the inpatient rehabilitation program.      - - Health-related QoL (SF-36):     - Overall, no significant change was observed in the SF-36 global score.     - However, specific subscales (Physical Functioning, Vitality, Role-Emotional) showed a positive correlation with functional recovery at discharge (p < 0.01), suggesting partial alignment between perceived and objective recovery.      - - Individualized QoL (SEIQoL-DW):     - No significant improvement was found between T1 and T2.     - Suggests that subjective quality of life may not improve in the short term, despite functional gains.      - - Emotional distress (HADS):     - Depression and anxiety scores did not significantly change from T1 to T2.     - However, depression scores were negatively correlated with perceived QoL at discharge (p < 0.01), especially in the Vitality and Social Functioning domains.      - - Coping strategies (COPE):     - Adaptive coping styles such as Acceptance and Positive Reinterpretation and Growth were positively associated with better psychological and functional outcomes at discharge (p < 0.05–0.01).     - Maladaptive strategies (e.g., Behavioral Disengagement) were associated with worse outcomes.      - - Perceived social support (MSPSS):     - High perceived support from family and friends was significantly associated with better emotional well-being and functional outcomes (p < 0.01).     - Perceived support did not significantly change over time but played a protective role. | N/A | Barthel Index – functional status    SF-36 (Short Form-36 Health Survey) – health-related quality of life    SEIQoL-DW (Schedule for the Evaluation of Individual Quality of Life – Direct Weighting) – patient-centered QoL    HADS (Hospital Anxiety and Depression Scale) – psychological distress    COPE Questionnaire – coping strategies    MSPSS (Multidimensional Scale of Perceived Social Support) – perceived social support | N/A | Significant improvement in functional status (Barthel Index):  p < 0.01 from T1 to T2  No significant overall improvement in individualized or most health-related QoL scores    Strong correlations at T2 between:  Functional status and SF-36 subscales (e.g., Physical Functioning, Vitality): p < 0.01  Depression and lower QoL: p < 0.01  Adaptive coping (e.g., Acceptance, Positive Reinterpretation) and better mental health outcomes: p < 0.05–0.01  Family and friend support associated with emotional and functional recovery: p < 0.01 | N/A | N/A |
| Fama et al.[29] | - - **Design:** Pilot observational study in an acute inpatient rehabilitation hospital (USA).   - **Participants:** 10 adults with severe non-fluent aphasia, 2–11 weeks post-left hemisphere stroke.   - **Data collection:** Video-recorded 60-minute group and individual speech-language therapy sessions (same SLP, blinded to study purpose).   - **Therapy structure:**     - Group: Social interaction focus (Augmentative Communication Group activities).     - Individual: Impairment-focused (comprehension, speech, reading/writing).   - **Analysis:** Sessions transcribed and coded (communication initiations, interaction targets, expression modes); Wilcoxon signed-rank tests used for comparisons. | In-patient observation in a real-world hospital setting    Participants were observed during one group session and one individual session on the same day | Total number of communicative initiations :   - Significantly higher in group therapy - Mean: 12.7 (group) vs 8.5 (individual) - Wilcoxon Z = 2.045; p = 0.041     Modes of initiation (e.g., vocalization, facial expression, gesture) :   - Participants produced significantly more vocalizations during group therapy (p = 0.026) - More facial expressions were observed in group settings (p = 0.031) - No significant differences in use of gestures or communication boards     Purposes of initiation (e.g., social closeness, task-based) :   - Initiations aimed at social closeness (e.g., greetings, shared humor) were significantly more common in group therapy (p = 0.007) - Participants displayed a greater variety of initiation purposes in group sessions (p = 0.027)     Targets of initiation (e.g., therapist, peer) :   - In group therapy, participants directed initiations toward peers as well as therapists, whereas individual sessions were directed only at the therapist - Variety of initiation targets was significantly greater in group therapy (p = 0.007)     Yes/No response accuracy and automatic speech:   - No significant differences between group and individual settings: - Yes/No accuracy: p = 0.31 - Automatic speech accuracy (e.g., counting): p = 0.44     Lexical diversity (number of different, real words used) :   - Participants used fewer different, real words in group therapy than in individual therapy (p = 0.033), likely reflecting the different structure and focus of the sessions | Group therapy promoted social-pragmatic communication  Participants were significantly more likely to initiate communication for the purpose of social closeness (e.g., humor, greeting, acknowledgment) in group settings.  This suggests that group therapy may be particularly effective for fostering real-world, socially meaningful interactions, even in individuals with severe aphasia.    Group therapy encouraged broader engagement, whereas in individual therapy, communication was exclusively directed toward the therapist.  This demonstrates the peer interaction potential of group settings and their role in rebuilding communicative confidence and spontaneity.    Group therapy supported nonverbal and multimodal expression  These modes may be underutilized in individual therapy but are essential for communicative participation in the real world.    Lexical output was greater in individual therapy  This highlights the different therapeutic focus: individual sessions supported more language output, while group sessions enhanced functional and social engagement.    Task structure influenced language behaviors  Individual sessions were more impairment-focused, supporting structured output but offering fewer naturalistic cues for interaction.    Severe aphasia patients can meaningfully participate in group therapy | N/A | N/A | Participants initiated more communication during group vs. individual therapy  Mean: 12.7 (group) vs. 8.5 (individual)    Wilcoxon Z = 2.045, p = 0.041    Significantly more:  Vocalizations (p = 0.026)  Facial expressions (p = 0.031)  Initiations for social closeness (p = 0.007)  Variety in initiation targets and types (p = 0.007, p = 0.027) | N/A | N/A |
| Manning et al.[30] | - - **Design:** Cross-sectional online survey of speech and language therapists (SLTs) in Ireland.   - **Aim:** Explore delivery of psychosocial support to people with aphasia, guided by Normalization Process Theory (NPT).   - **Survey content:** 40 items across five domains (demographics, practices, collaboration, barriers, role perceptions).   - **Participants:** 54 SLTs working in Irish HSE settings with aphasia patients.   - **Distribution:** Professional mailing lists and social media; voluntary and anonymous participation.   - **Analysis:** Descriptive statistics (frequencies and percentages only). | Online survey distributed via professional mailing lists and social media | 98% of SLTs agreed it is part of their role to deliver psychosocial support. However, many felt underprepared or lacked training in formal psychosocial approaches.    Common practices included:   - Supportive listening (100%) - Collaborative goal-setting (85%) - Working with families (81%) - Encouraging social participation (76%) - Coordinating with other professionals (74%)     Less common practices (due to scope-of-practice concerns or lack of training) included:   - Referring to psychologists or counselors (52%) - Direct use of formal approaches like CBT, SFBT, or MI (<5%)     Key barriers to implementation included:   - Limited time and staffing (67%) - Lack of access to specialist mental health services - Inconsistent service models across settings (acute vs. community)     Variation by setting:   - SLTs in acute or subacute settings reported less time and fewer psychosocial interventions than those in community or rehabilitation settings - SLTs often felt isolated in delivering this care - Many reported limited collaboration with mental health professionals, despite recognizing the need | Role legitimacy is influenced by systemic and contextual factors  While SLTs endorsed psychosocial work as legitimate, the degree to which they could enact it depended on their workload, setting (acute vs. community), and team structures.    Interdisciplinary collaboration was inconsistent and often insufficient  Service inequities emerged across different settings  SLTs in community-based services reported greater capacity and flexibility to address psychosocial needs, while those in acute and subacute hospitals faced time pressure, high caseloads, and restricted scopes of practice.    Informal support strategies dominate current practice | N/A | N/A | N/A | N/A | N/A |
| Balasooriya-Smeekens et al.[31] | - - **Design:** Qualitative study using focus groups (Cambridgeshire, UK).   - **Aim:** Explore how primary care can better support stroke and TIA survivors returning to work.   - **Participants:** 18 individuals (stroke/TIA survivors, caregivers, GPs, occupational therapists, commissioners, employer representative).   - **Data collection:** Four focus groups at GP practices/universities; semi-structured topic guide used.   - **Analysis:** Framework analysis with systematic coding, thematic charting, and team discussions. | Via four in-person focus groups, held in GP practices and university settings in Cambridgeshire | The study identified four major themes from the focus group discussions:   1. Primary care is not currently seen as a key player in return-to-work support   GPs are perceived as reactive rather than proactive, and often lack time or training to address work reintegration.  Participants emphasized a need for a designated person or point of contact in primary care to coordinate vocational support.     1. Fragmentation and poor communication across services      1. The psychosocial complexity of returning to work   Survivors highlighted low confidence, fatigue, cognitive issues, and fear of failure as major barriers.     1. The need for proactive, tailored, and integrated support | GPs and other professionals acknowledged a lack of training and guidance on how to support return to work, especially for managing hidden impairments (e.g., cognitive fatigue).    Caregivers are essential but unsupported actors in return to work  Work participation is a major determinant of post-stroke identity and wellbeing | N/A | N/A | N/A | - - Stroke and TIA survivors felt their return-to-work (RTW) needs were largely unmet by healthcare services.   - There was frustration over the lack of structured vocational support and poor involvement of primary care.   - No single professional coordinated RTW efforts, leading to fragmented care and repeated retellings of their history.   - Psychological barriers like low confidence, anxiety, and cognitive fatigue were often overlooked in follow-up care.   - Survivors wanted proactive, personalized, and continuous support rather than isolated advice.   - Practical help was valued but inconsistently available, especially for those discharged without structured rehabilitation.   - Early conversations about RTW were rare unless initiated by the patient.   - Overall, the system was seen as reactive, poorly coordinated, and ill-equipped to manage work reintegration challenges. | - - Caregivers felt underprepared and unsupported in helping stroke and TIA survivors return to work.   - Many assumed informal coordinator roles, managing resources, employer communication, and service navigation without professional guidance.   - Frustration was common due to the absence of structured pathways and designated contacts for vocational recovery.   - Caregivers experienced emotional burden, balancing these responsibilities alongside daily caregiving tasks.   - Vocational and psychological recovery needs were often neglected, forcing caregivers to address critical service gaps.   - There was a strong call for clearer communication, better information, and formal caregiver inclusion in return-to-work planning.   - Services were widely perceived as fragmented, insufficient, and disconnected from the realities of employment resumption after stroke or TIA. |
| Moore et al.[32] | - - **Design:** Qualitative study embedded in an RCT of early vocational rehabilitation (Victoria, Australia).   - **Aim:** Explore stroke survivors' experiences with vocational rehabilitation (VR) or usual care.   - **Participants:** 16 employed adults post-stroke (9 women, 7 men), from both intervention and control groups.   - **Data collection:** Semi-structured interviews (30–75 minutes) via telephone or videoconference (July 2020–January 2021).   - **Analysis:** Framework analysis with coding based on predefined VR and recovery categories; cross-case comparisons made; thematic saturation achieved. | The early VR trial consisted of  Weekly 1-hour sessions (up to 12 weeks)  Tailored support including:   - Workplace liaison (contacting employers) - Fatigue management plans - Executive functioning strategies (e.g., planning, memory aids) - Support with modified duties or new roles - Collaborative return-to-work planning     The interviews of the qualitative component consisted of interviews via telephone and videoconferencing (participant homes) | The analysis revealed four key themes:     1. “I’ve still got a job to go back to” – The value of early intervention   Participants in the VR group appreciated having early, structured RTW support, especially while still identifying as a worker.  Early engagement was critical for maintaining employment identity and motivation.  Those who did not receive VR often described uncertainty or fear about work, and some had already lost their job by the time support was offered.     1. “It’s not one size fits all” – The need for tailored, flexible support   VR recipients highlighted the value of individualized strategies: workplace liaison, fatigue management, memory aids, and emotional support.  Participants emphasized the importance of therapists understanding their job demands, and tailoring support accordingly.  In contrast, those in the control group often found the generic information they received irrelevant or unhelpful.     1. “I didn’t know that was even a thing” – Limited awareness of vocational services      1. “It’s not just about work” – The psychosocial impact of returning to work   Those who returned to work successfully, especially with support, reported improved mental health and motivation.  Those who struggled to return described increased emotional distress, loss of purpose, and uncertainty about their future. | Vocational rehabilitation supported emotional recovery  VR participants described feeling more confident, less isolated, and emotionally supported through the structured sessions.  The therapist’s presence as a consistent point of contact contributed to mental well-being, even when work outcomes were uncertain.    Participants wanted workplace coordination and advocacy  Participants appreciated help explaining stroke-related challenges to employers and navigating modified duties or phased returns.    Therapist knowledge of job demands was critical  Many were unaware that returning to work could be supported by a healthcare provider, relying instead on trial-and-error or employer goodwill.    Success or failure in returning to work had ripple effects on self-worth, financial stability, and family dynamics. | N/A | N/A | N/A | - - Stroke survivors who received early vocational rehabilitation (VR) reported high satisfaction, valuing the timely, personalized, and supportive approach.   - VR helped maintain a sense of worker identity and provided concrete strategies like fatigue management and employer liaison.   - Participants felt supported, listened to, and emotionally bolstered, aiding their work reintegration.   - In contrast, those in the usual care group described services as minimal, fragmented, or absent regarding vocational needs.   - Many usual care participants were unaware such support existed and felt isolated and anxious about returning to work.   - The VR intervention was seen as essential and meaningful, while standard care revealed major gaps for working-age survivors. | N/A |
| Bērziņa et al.[33] | - - **Design:** Retrospective-prospective study of stroke survivors post-inpatient neurorehabilitation at NRC “Vaivari.”   - **Participants:** 255 stroke survivors discharged between 2008–2012; contacted by phone and surveyed via post or electronically.   - **Data collection:**     - Retrospective: Functional Independence Measure (FIM) scores at discharge.     - Prospective: WHODAS 2.0 (36-item) + sociodemographic questionnaire.   - **Timing:** Median 20 months post-discharge (IQR: 11–30 months).   - **Analysis:** Descriptive statistics and multiple linear regression to identify discharge-time predictors of long-term self-reported disability. | Participants received **specialized inpatient neurorehabilitation** at NRC Vaivari. The level of independence at discharge was assessed using the **Functional Independence Measure (FIM)** across domains like self-care, locomotion, communication, etc.    **Retrospective data collection** from clinical records (FIM)  Participants were contacted **by phone**, and surveys were **mailed or emailed** for home completion | Overall self-perceived disability (WHODAS 2.0 total score) was moderate, with significant variability across participants.    Key predictors of higher self-perceived disability included:  Lower functional independence at discharge (especially in self-care and locomotion)  Working participants reported better WHODAS scores across nearly all domains, especially in life activities and social participation (p < 0.01).  Unemployment or retirement status post-stroke  Associated with worse scores in life activities, mobility, and social participation (p < 0.01)  Older age  Associated with higher disability, especially in mobility and life activities domains (p < 0.05)  Living alone  Linked to lower participation scores (p = 0.04), indicating the protective role of household support.  Language preference (Russian vs. Latvian)  Russian speakers reported more disability in participation (p < 0.01), suggesting possible cultural, linguistic, or service access disparities that warrant targeted follow-up.    The regression model explained:  40.8% of the variance in total WHODAS score  23–43.5% of variance across specific WHODAS domains    This suggests that non-clinical factors (e.g., social context, personal background) are highly relevant in shaping the lived experience of disability after stroke. | Sociodemographic factors significantly influenced perceived disability    Beyond functional status at discharge, several personal and environmental factors were found to significantly affect long-term disability perception.      Living alone was associated with reduced participation in society (p = 0.04)  Language preference (Latvian vs. Russian) linked to participation restrictions    Russian-speaking participants reported significantly greater limitations in social participation (p < 0.01)    Age influenced mobility and life activities    Older participants had significantly worse outcomes in mobility and life activities, regardless of their discharge FIM scores (p < 0.05), emphasizing age-related vulnerability in post-stroke recovery.    Functional independence domains had differing predictive value   - Among the FIM subdomains, self-care and locomotion were the strongest predictors of total and domain-specific WHODAS outcomes. - In contrast, communication and social cognition FIM scores had weaker associations with long-term perceived disability. | WHODAS 2.0 – WHO Disability Assessment Schedule, 36-item version : self-perceived disability across six domains + work/school activities  Cognition  Mobility  Self-care  Getting along with people  Life activities (including work/school)  Participation in society    FIM (Functional Independence Measure) – recorded at discharge from inpatient rehabilitation, grouped by functional domains    Sociodemographic variables and work status | N/A | Multiple regression models explained 23%–43.5% of variance in WHODAS 2.0 domains    Total WHODAS score explained: 40.8%    Significant predictors:  Self-care and locomotion dependence at discharge strongly associated with worse self-perceived disability (p < 0.01)    Work status: those working reported better functioning in almost all domains (p < 0.01)    Age: older participants had higher disability scores (p < 0.05)    Preferred language (Latvian vs. Russian): Russian-speakers reported more participation restrictions (p < 0.01)    Living alone associated with lower participation (p = 0.04) | N/A | N/A |
| White et al.[34] | - - **Aim:** Identify predictors of health-related quality of life (HRQoL) over 12 months post-stroke.   - **Participants:** 134 stroke survivors (ischemic or hemorrhagic) recruited from two public tertiary hospitals.   - **Data collection:** Face-to-face interviews at baseline, 3, 6, 9, and 12 months; conducted at participants' homes or preferred locations.   - **Measures:** HRQoL (AQoL), disability (mRS), emotional distress (HADS), social support (MSPSS), community participation (AAP).   - **Analysis:** Linear mixed models to examine associations between HRQoL and age, disability, emotional distress, social support, community participation, and baseline HRQoL.   - **Design:** Observational; no intervention; naturalistic recovery focus. | Face-to-face interviews conducted by the primary researcher at baseline, 3, 6, 9, and 12 months | The mean AQoL score at baseline was 0.547, substantially lower than the general population average (~0.75), indicating impaired quality of life in stroke survivors.    The final multivariable linear mixed-effects model showed that higher HRQoL over 12 months was significantly associated with:   - Younger age (p = .006) - Lower disability (mRS) (p = .003) - Greater community participation (AAP) (p < .001) - Higher baseline HRQoL (p = .032) - No history of depression (p = .031)   A past history of depression was associated with persistently lower HRQoL throughout follow-up (p = .031), even when current depression scores (HADS) were not statistically significant.    Perceived social support (MSPSS) was included in univariate analysis but was not retained in the final model.    Current anxiety and depression levels (HADS) were not statistically significant predictors in the final model.  This suggests that while emotional distress is common, its long-term impact on QoL may be mediated by other factors like disability and lifestyle.    Time since stroke (from 3 to 12 months) did not independently influence HRQoL, indicating that improvement over time was not automatic and depended more on personal and functional factors. | Functional disability influenced HRQoL more than age or emotional status  Higher Modified Rankin Scale (mRS) scores—indicating greater disability—were strongly and independently associated with poorer HRQoL (p = .003), confirming the functional basis of quality-of-life limitations. | Assessment of Quality of Life (AQoL) — primary outcome measure for HRQoL    Modified Rankin Scale (mRS) — disability    Hospital Anxiety and Depression Scale (HADS) — anxiety and depression    Multidimensional Scale of Perceived Social Support (MSPSS) — social support    Adelaide Activities Profile (AAP) — community participation and lifestyle | 3, 6, 9, and 12 months post-stroke | Higher HRQoL was significantly associated with:  Younger age (p = .006)  Lower disability (mRS) (p = .003)  Greater community participation (AAP) (p < .001)  No prior history of depression (p = .031)  Higher baseline HRQoL (p = .032)  Time since stroke and current anxiety were not significantly associated with HRQoL over 12 months  Baseline AQoL mean score: 0.547  Population norm in same age group: ~0.75 → Stroke survivors scored substantially lower | N/A | N/A |
| Delhey et al.[35] | - - **Aim:** Examine associations between neighborhood resource density and stroke outcomes (mortality, recurrence, physical function, cognition, QoL, depression).   - **Participants:** 1786 stroke survivors for mortality/recurrence; subset of 1284 for 3-month outcomes.   - **Data source:** Brain Attack Surveillance in Corpus Christi (BASIC) project (2009–2019).   - **Exposure:** Neighborhood resource density (low, medium, high) assessed via NaNDA and geocoded addresses.   - **Outcomes:** Functional independence (ADL/IADL), cognition (3MSE), QoL (SS-QoL), depression (PHQ-8); mortality and recurrence over median 3.8 years.   - **Analysis:** Cox proportional hazards models (mortality, recurrence) and multivariable linear regression (functional, cognitive, psychosocial outcomes), adjusted for demographic and clinical factors. | Not a service — study investigated the impact of neighborhood resource density (e.g., stores, community centers, eateries) on post-stroke outcomes | - 1. All-cause mortality and stroke recurrence:      - No significant association between neighborhood resource density and mortality or recurrence.      - Cox proportional hazard models showed hazard ratios ≈ 1.0 across low, medium, and high resource density groups.      - p > 0.80 for all comparisons.      - 1. Functional status (ADL/IADL):      - Among participants with moderate to severe stroke, those living in resource-rich neighborhoods had better functional outcomes.      - Mean difference = –0.156 (lower scores = better function)      - p = 0.0176      - 1. Cognitive function (3MSE):      - Higher neighborhood resource density was associated with significantly better cognitive scores across the full sample.      - Mean difference = +0.838 points      - p = 0.0277      - 95% CI: 0.092 to 1.584      - 1. Quality of life (SS-QoL):      - Among moderate to severe stroke survivors, higher resource density predicted better quality of life.      - Mean difference = +0.194 (on a 1–5 scale)      - p = 0.0214      - 1. Depressive symptoms (PHQ-8):      - No significant relationship was found between resource density and depression scores.      - p > 0.05 for all comparisons | Neighborhood resource density was more impactful for moderate–severe stroke survivors  Among mild stroke survivors, resource density had no measurable impact on outcomes, suggesting that more vulnerable populations benefit most from supportive environments.    Functional, cognitive, and quality-of-life outcomes were positively associated with resource-rich neighborhoods, indicating that community infrastructure (e.g., stores, recreational centers, libraries) may promote greater activity, cognitive stimulation, and engagement, especially in the chronic recovery phase.    A small but statistically significant improvement in 3MSE scores was observed in participants from high-resource areas. This suggests that environmental enrichment may support cognitive maintenance or recovery post-stroke (p = 0.0277), even when clinical care access is not the focus.    Depression outcomes were unaffected by neighborhood resources    No effect on long-term survival or recurrence    Ethnicity and sex did not modify the effects of neighborhood resources | Activities of Daily Living/Instrumental ADL (custom summary score, range 1–4)    Modified Mini-Mental State Exam (3MSE) — cognition (range: 0–100)    Abbreviated Stroke-Specific Quality of Life scale (SS-QoL) (range: 1–5)    Patient Health Questionnaire-8 (PHQ-8) — depressive symptoms (range: 0–24) | Mortality and recurrence: median follow-up ~3.8 years    Functional, cognitive, depression, and QoL outcomes: assessed at 3 months post-stroke | Greater neighborhood resource density was associated with:  Better cognition: 3MSE mean difference = +0.838, p = 0.0277 (95% CI: 0.092–1.584)  Better functioning (ADL/IADL) among moderate–severe stroke survivors: mean difference = –0.156, p = 0.0176  Better QoL (SS-QoL) among moderate–severe stroke survivors: mean difference = +0.194, p = 0.0214    No association found with:  Depression (PHQ-8)  All-cause mortality or stroke recurrence (HR ≈ 1.0, p > 0.80)  Effect sizes were minimal (Hedges’ g < 0.2)  No significant interactions by sex or ethnicity; moderate interaction by stroke severity | N/A | N/A |
| Ng et al.[36] | - - **Aim:** Evaluate long-term effects of the Occupational Lifestyle Redesign Programme (OLSR) on motivation, community integration, and quality of life post-stroke.   - **Participants:** 50 stroke survivors (25 OLSR + standard OT; 25 matched controls with conventional OT only).   - **Intervention:** 8-session group-based OLSR program (goal-setting, self-efficacy, physical/emotional health, social engagement).   - **Follow-up:** Mean 6.2 years post-stroke; outcomes assessed via telephone interviews.   - **Measures:** SA-SIP30, GAMM, CIQ, WHO-5.   - **Analysis:** ANOVA and regression models for group comparisons and predictors of well-being. | 8-session Occupational Lifestyle Redesign Programme (OLSR)    Focused on goal setting, self-efficacy, productivity, emotional well-being, and community re-engagement    Based on Bandura’s self-efficacy theory and Stanford’s Chronic Disease Self-Management model    Included action planning, peer support, and voluntary service engagement | - 1. Physical Functioning and Disability (SA-SIP30):   The OLSR group showed significantly better scores on:   - - Mobility subscale: F = 4.47, p = .04   - Household Management subscale: F = 6.42, p = .015   - Indicates long-term benefit of OLSR in preserving daily functional independence.      - 1. Motivation (GAMM):      - OLSR participants had higher activity motivation scores than controls (mean 41.3 vs. 35.4)      - Result showed a trend toward significance: p = .06      - Suggests positive influence of OLSR on maintaining internal drive for daily activity and engagement.      - 1. Community Integration (CIQ):      - Social integration subscore was significantly higher in the OLSR group: F = 4.30, p = .043      - Total CIQ score improved significantly in the OLSR group over time: F = 5.74, p = .040      - 1. Psychological Well-being (WHO-5):      - WHO-5 scores were significantly higher in the OLSR group: F = 5.52, p = .023      - Suggests enhanced long-term emotional health in participants who underwent lifestyle redesign.      - 1. Predictors of Well-being:      - Regression analysis showed GAMM (motivation) was the strongest predictor of psychological well-being WHO-5 scores: F = 13.11, p = .001. This suggests that programs enhancing self-efficacy and intrinsic activity drive are key to long-term emotional recovery. | Participants in the OLSR group demonstrated functional and psychosocial improvements up to 6.2 years post-stroke, indicating that structured behavioral interventions can produce enduring effects even when offered in the chronic phase of stroke recovery.    Community integration improvements were most notable in social participation including re-engagement with peers, group activities, and volunteer work — key contributors to perceived life satisfaction.    Functional improvements were observed in mobility and household management, but not in all areas of the SA-SIP30, suggesting that OLSR had a targeted impact where self-efficacy and goal planning could be practically applied.    Participants who lived with others showed higher community and social involvement  Only 8% of participants lived alone, implying a potential supportive role of cohabiting family in sustaining participation and reinforcing OLSR outcomes. | SA-SIP30 (Stroke-Adapted Sickness Impact Profile-30) — handicap and physical/emotional functioning    GAMM (General Activity Motivation Measure) — activity motivation    CIQ (Community Integration Questionnaire) — community participation    WHO-5 (WHO-5 Well-Being Index) — subjective psychological well-being | Mean follow-up time was 6.2 years post-stroke | SA-SIP30 (Mobility and Household Management subscales):   - Significant improvement in OLSR group - Mobility: F = 4.47, p = .04 - Household Management: F = 6.42, p = .015     GAMM (motivation):   - Higher in OLSR group (mean = 41.3 vs 35.4) - Trend toward significance: p = .06     CIQ (Community Integration):   - Social subscore significantly higher in OLSR group (F = 4.30, p = .043) - Total CIQ improved significantly over time in OLSR group (F = 5.74, p = .040)     WHO-5 (Well-being):   - Significantly higher in OLSR group (F = 5.52, p = .023)     Regression analysis:   - Motivation (GAMM) was the best predictor of well-being (WHO-5) (F = 13.11, p = .001) | N/A | N/A |
| Egan et al.[37] | - - **Aim:** Examine reciprocal relationships between participation and well-being over two years post-stroke.   - **Participants:** 67 community-dwelling stroke survivors (FIM cognitive score ≥3); aged 33–88 years.   - **Data collection:** In-home interviews at 6, 9, 12, 18, and 24 months post-stroke.   - **Measures:** Participation (RNLI), emotional well-being (GWB), physical health (GHQ), baseline impairment (FIM), neighborhood income.   - **Analysis:** Lagged regression models testing bidirectional effects and moderation by individual/contextual factors (age, sex, FIM, neighborhood income). | N/A | Specifically, it tested whether:   - Participation at a given time point predicted well-being at a later time point - Well-being at a given time point predicted later participation     **Participation (measured by the Reintegration to Normal Living Index (RNLI)) → Later Emotional Well-being**  Higher participation scores at an earlier time point (t−1) predicted higher emotional well-being at the following time point (t)  Regression coefficient (B) = 0.156, p < .01    Indicates a significant positive effect: greater engagement in life roles led to improved emotional states    **Emotional Well-being (measured by the General Well-Being Schedule (GWB))→ Later Participation**  Higher emotional well-being at t−1 also predicted higher participation at the next time point  B = 0.141, p < .05    Confirms a reciprocal relationship between emotional state and role re-engagement    **Participation → Later Physical Well-being**  The effect of participation on later physical well-being ( General Health Question - GHQ) showed a trend toward significance  p = .10    Suggests possible but weaker influence    Moderating Factors:   - Neighborhood income significantly moderated the effect of emotional well-being on later participation - Stronger positive effect seen in those from lower-income areas - FIM scores moderated the effect of physical well-being on participation - Greater functional independence strengthened this relationship     Non-significant predictors:  Age, sex, and time since stroke did not significantly influence the associations between participation and well-being | Participation and well-being function in a reciprocal loop. This highlights the importance of addressing both domains simultaneously in post-stroke rehabilitation strategies.    Participants with higher functional independence at baseline showed a stronger relationship between physical health perceptions and real-world engagement, indicating that rehabilitation gains can amplify the benefits of perceived health.    Physical well-being is less predictive than emotional well-being  Unlike emotional well-being, self-rated physical health did not significantly predict later participation (p = .10), suggesting that subjective mood and motivation may play a larger role in post-stroke re-engagement than physical status alone.      The bidirectional associations between participation and well-being held consistently across multiple time points (6, 9, 12, 18, and 24 months), reinforcing the long-term relevance of these psychological and behavioral factors in community-based recovery. | Reintegration to Normal Living Index (RNLI) — participation    General Well-being Schedule (GWB) — emotional well-being    General Self-rating of Health Question (GHQ) — physical well-being    Functional Independence Measure (FIM) — impairment level at discharge | Y5 in-home interviews conducted over 24 months | Participation predicted later emotional well-being:  RNLI at t−1 → GWB at t: B = 0.156, p < .01    Emotional well-being predicted later participation:  GWB at t−1 → RNLI at t: B = 0.141, p < .05    Physical well-being prediction by participation was marginally significant:  RNLI at t−1 → GHQ at t: p = .10    Moderating effects:  Income moderated the effect of emotional well-being on participation    FIM moderated the effect of physical well-being on participation    No significant effects of age, sex, or time since stroke on these associations | N/A | N/A |
| Tielemans et al.[38] | - - **Intervention:** Six weekly 2-hour group sessions + one booster session at week 10; delivered by two trained therapists in hospitals/rehabilitation centers.   - **Content focus:** Proactive coping, goal setting, peer support, emotional self-regulation; use of Proactive Action Planning Tool.   - **Participants:** Groups of 4–6 stroke survivors, with optional partner involvement.   - **Data collection:** Post-session evaluations (patients/partners), therapist session logs (fidelity), therapist focus groups (qualitative feedback).   - **Findings:** High satisfaction overall; Proactive Action Planning Tool fully applied in ~80% of sessions; no long-term follow-up. | In-person group sessions and post-intervention surveys, session logs, and focus groups (therapists)    10-week program: six two-hour sessions plus one booster session in week 10    Included patients and partners (if available)    Led by two trained therapists    Focused on proactive action planning, peer support, coping with emotions, and addressing invisible stroke consequences    Therapists trained in solution-focused coaching | - 1. Participant Satisfaction with the intervention:   Overall satisfaction scores (scale 1–10):   - - Patients: Mean = 7.5 (SD 1.6)   - Partners: Mean = 7.8 (SD 0.7)   - Therapists: Mean = 7.4 (SD 0.7)     Satisfaction was consistently high across all groups.     - 1. Perceived Usefulness:   90% of patients, 100% of partners, and 100% of therapists rated the intervention as “somewhat” to “very” useful.    The program was especially valued for its emphasis on peer support and addressing invisible stroke-related consequences.  Their involvement promoted joint reflection and shared planning, reinforcing dyadic coping strategies.     - 1. Implementation Fidelity — particularly the use of the Proactive Action Planning Tool :   The Proactive Action Planning Tool, central to the program, was fully applied in only 80% of sessions.    In 20% of sessions, its use was inadequate or omitted, often due to time constraints or therapist discomfort with the structure.     - 1. Group Dynamics and Peer Support:   Peer support emerged as the most valued component for all stakeholders.    Participants reported that sharing experiences, emotions, and coping strategies helped normalize their challenges and encouraged hope.     - 1. Therapist Feedback (from focus groups):   Therapists appreciated the structured format but cited challenges with integrating partners, balancing emotional discussions with planning tasks, and limited time to fully complete all intended content. | The program addressed “invisible” consequences of stroke    Participants noted that the intervention uniquely validated and addressed emotional, cognitive, and psychosocial challenges that are often underrecognized in conventional rehab.  Partner integration required careful facilitation  While the inclusion of partners was a strength, therapists identified a need for clearer guidance on how to involve partners without dominating the session or shifting focus away from the patient. | Barthel Index — ADL independence (baseline)    Patient, partner, and therapist evaluation forms — satisfaction and perceived usefulness    Therapist session logs — engagement, goal-setting, homework, action plan use    Focus group discussions — qualitative assessment of feasibility, satisfaction, and implementation barriers | N/A | Satisfaction scores (scale 1–10):   - Patients: mean 7.5 (SD 1.6) - Partners: mean 7.8 (SD 0.7) - Therapists: mean 7.4 (SD 0.7)     Perceived usefulness:   - 90% of patients, 100% of partners, and 100% of therapists found the intervention “somewhat to very useful” - Proactive action plan tool properly applied in only 80% of sessions - 20% of sessions showed inadequate implementation of the tool - Peer support was the most valued component among patients, partners, and therapists | - - Stroke survivors reported high satisfaction with the service, appreciating the structured group setting for emotional reflection and support.   - Peer support was the most valued aspect, helping participants feel reassured and less isolated.   - The intervention helped address "invisible" consequences of stroke, such as fatigue, emotional changes, and uncertainty about the future.   - Although the planning tool was inconsistently used, sessions were still perceived as emotionally supportive and motivational.   - Overall, the service highlighted the importance of integrating emotional validation, peer exchange, and structured planning into post-stroke rehabilitation. | - - Caregivers reported a very positive experience, with an average satisfaction score of 7.8/10 and universal agreement on the intervention's usefulness.   - Joint participation with stroke survivors strengthened communication, mutual understanding, and shared planning.   - The program helped caregivers cope with emotional challenges, understand invisible stroke consequences, and feel less isolated.   - Interaction with other caregivers provided important psychosocial support.   - Therapists noted that while caregiver involvement was generally beneficial, it sometimes risked shifting focus away from the stroke survivor.   - Overall, family participation was seen as enhancing the rehabilitation process for both patients and caregivers. |
| Joana Matos et al.[39] | - - **Participants:** 553 stroke survivors previously employed at stroke onset; discharged from public Stroke Units (2019–2021).   - **Data collection:** Structured telephone interviews (18–24 months post-stroke); in-person proxy assessments when needed.   - **Measures:** HADS (depression/anxiety), CIQ (community integration), SS-QoL, Barthel Index, modified Rankin Scale; sociodemographic and clinical data.   - **Focus:** Naturalistic return to work (RTW) process; 91.5% returned without formal vocational rehabilitation.   - **Analysis:** Multiple linear regression models assessing associations between RTW status and community integration, quality of life, and mental health. | Telephone interviews using structured questionnaires | - 1. Community Integration (CIQ):   Stroke survivors who had returned to work (RTW) showed significantly higher levels of community integration across all CIQ domains:   - - Global CIQ score:   β = 3.59, 95% CI: 3.39–3.79   - - Home integration:   β = 5.48, 95% CI: 4.74–6.23   - - Social integration:   β = 1.10, 95% CI: 0.62–1.58   - - Productive activity:   β = 0.79, 95% CI: 0.45–1.13     - 1. Mental Health (HADS):   RTW was associated with higher depressive symptoms, even though participants were functionally independent:   - - HADS-Depression subscale (HADS-D):   β = 0.63, 95% CI: 0.20–1.46    No significant association was found between RTW and HADS-Anxiety scores.     - 1. Quality of Life (SS-QoL):   No significant difference was observed in overall SS-QoL scores between those who returned to work and those who did not.    However, specific QoL subdomains improved among those who had returned to work:   - - Mood domain:     - β = 1.07, 95% CI: 0.06–2.08   - Personality domain:   β = 0.72, 95% CI: 0.01–1.44    No difference in the work/productivity domain | Return to work (RTW) was achieved without formal support in most cases (91.5%)  RTW was more common among blue-collar than white-collar workers, perhaps due to lower expectations or faster physical recovery requirements.    A key paradox emerged: although RTW was linked to better CIQ scores in all domains (home, social, productive), it also correlated with higher depressive symptom scores (HADS-D), suggesting that occupational reintegration may carry emotional costs such as stress, fatigue, or fear of failure.    RTW had no impact on work-related QoL perception (no improvement of the "work/productivity” subdomain of the Stroke-Specific QoL Scale)  This suggests that while patients were employed again, they may not have felt truly fulfilled or productive in the same way as pre-stroke.    Better mood and personality profiles(better outcomes in SS-QoL subdomains) in RTW participants despite elevated depressive scores.    Functional independence and comorbidities were not barriers to RTW in this cohort | Hospital Anxiety and Depression Scale (HADS) — mental health    Stroke-Specific Quality of Life Scale (SS-QoL) — QoL    Community Integration Questionnaire (CIQ) — community reintegration    Modified Rankin Scale (mRankin) — functional dependence    Barthel Index — ADL autonomy | N/A | Community Integration (CIQ)  RTW associated with better integration across all CIQ domains   - Global CIQ score: β = 3.59, 95%CI: 3.39 to 3.79 - Home integration: β = 5.48, 95%CI: 4.74 to 6.23 - Social integration: β = 1.10, 95%CI: 0.62 to 1.58 - Productive activity: β = 0.79, 95%CI: 0.45 to 1.13     Depression symptoms (HADS-D):  RTW associated with higher depressive symptoms  β = 0.63, 95%CI: 0.20 to 1.46  No significant association with anxiety    QoL (SS-QoL):  No significant difference in global QoL between RTW and non-RTW  Specific subdomains improved:  Mood: β = 1.07, 95%CI: 0.06 to 2.08  Personality: β = 0.72, 95%CI: 0.01 to 1.44    No change in work/productivity or overall score | N/A | N/A |
| Takeru Umemura et al.[40] | - - **Participants:** 483 stroke patients who participated in the HES initiative (2015–2021).   - **Program structure:** Two stages — inpatient phase and follow-up coordination with the workplace.   - **Support model:** Dual-support coordinators (e.g., medical social workers) facilitated hospital-employer communication and workplace accommodations.   - **Data collection:** Retrospective review of quarterly hospital implementation reports.   - **Outcomes measured:** Return-to-work status at 3, 6, and 24 months; neurological deficits, ADL independence, occupational category.   - **Analysis:** Kaplan–Meier survival curves and chi-square tests to identify predictors of employment reintegration. | Acute treatment, stroke rehabilitation (physiotherapy, occupational therapy, speech therapy), and workplace interventions | Return to work (RTW) rate among stroke survivors who participated in the Health and Employment Support (HES) Program :   - 63.8% of participants returned to work during the observation period - The majority of RTW occurred within 6 months of stroke onset     Time-based RTW Distribution:   - 46.2% returned to work by 3 months - 57.3% by 6 months - 63.8% by 24 months     Impact of Stroke Type:  RTW rate was highest in patients with cerebral infarction  Lowest in those with subarachnoid hemorrhage    This difference was statistically significant (P = 0.0003)    Impact of Neurological Symptoms:  Patients without hemiplegia, aphasia, or unilateral spatial neglect were significantly more likely to return to work  Each of these deficits was independently associated with delayed or failed RTW (P = 0.0001)    Impact of Functional Status:  Patients who were independent in Activities of Daily Living (ADLs) at discharge had a significantly higher RTW rate (P = 0.0001)    Statistical Significance:  Kaplan–Meier analysis and log-rank test confirmed significant group differences in RTW trajectories (all P < 0.01) | A significant portion of stroke survivors (over 70% of those who eventually returned to work) did so within the first 6 months after stroke onset.  This suggests that early intervention and coordination during hospitalization may be critical for successful reintegration.    There was a notable occupational breakdown:  204 white-collar workers  279 blue-collar workers    The Health and Employment Support (HES) program was feasibly implemented in 29 hospitals, showing the scalability of this model.    The use of dual-support coordination allowed direct contact with employers, facilitating job modifications, gradual reintegration, and planning around residual impairments. | Activities of Daily Living (ADL) independence status    Neurological findings (e.g., hemiplegia, aphasia, neglect)    Return to Work (RTW) status at various time points (3, 6, and 24 months)    Kaplan–Meier survival curves, Chi-square test, log-rank test | up to 24 months or until RTW achieved | Significant predictors for RTW included stroke type (P = 0.0003), neurological deficits (P = 0.0001), and ADL independence (P = 0.0001)    Kaplan–Meier method showed significant RTW differences based on stroke severity and type (log-rank test, P < 0.01) | N/A | N/A |
| Birgitta Langhammer et al.[41] | - - **Aim:** Explore return-to-work (RTW) rates in stroke survivors after specialized rehabilitation for moderate-to-severe disability.   - **Participants:** 230 stroke survivors recruited during inpatient neurorehabilitation; pre-stroke employed; communicative and consenting.   - **Data collection:** Baseline at discharge (NIHSS, mRS, Barthel/FIM, demographics, LiSat-11); follow-up at 6 and 12 months via structured interviews.   - **Rehabilitation content:** Physical therapy, occupational therapy, ADL training, psychological support; minimal vocational training or employer coordination.   - **Follow-up services:** Physiotherapy and general medical care varied between countries. | Inpatient setting for intervention; data collection via semistructured interviews at 6 and 12 months in the community setting | Return to Work (RTW) rates at 6 and 12 months after discharge from specialized neurorehabilitation :   - At 6 months, 18% of participants had returned to work - At 12 months, this increased slightly to 20% - The majority of participants had not returned to work within the first year post-stroke     Predictors of RTW, including age, gender, education, functional status (mRS), and country of residence :   - Age was inversely associated with RTW at both time points - 6 months: p = 0.009 - 12 months: p = 0.048 - Education level positively predicted RTW at 6 months (p = 0.04) - Functional status at discharge, measured by mRS, was significantly associated with RTW at 6 months (p = 0.001) - Country of rehabilitation influenced RTW likelihood at 12 months (p = 0.03)     Life satisfaction across multiple domains using the Life Satisfaction Questionnaire (LiSat-11), especially the vocational domain :  Participants who returned to work showed higher satisfaction in the vocation domain, but overall satisfaction remained low    There was a negative correlation between mRS score (functional independence) and vocational satisfaction: ρ = −0.31 at 6 months, ρ = −0.29 at 12 months    Financial deterioration experienced by stroke survivors over time :  No formal economic evaluation was conducted, but financial strain was a consistent theme in follow-up interviews, especially among those who did not return to work  This deterioration was present even in publicly funded healthcare systems, indicating that loss of income and employment had a substantial economic impact. | Variability in Outcomes by Country:  This highlights the role of national healthcare structures, social services, and labor market policies in post-stroke reintegration.  For instance, RTW at 12 months varied between countries, with Norway showing relatively higher rates and Palestine, Russia, and Israel showing the lowest.    Lack of Post-Discharge Vocational Support as reported by the follow-up interviews  This gap likely contributed to the low RTW rates and poor economic/life satisfaction outcomes.    While gender was not a significant predictor in the multivariate models, fewer women returned to work in most centers, suggesting a potential gender disparity not fully explored in the statistical analysis. | NIHSS (National Institutes of Health Stroke Scale)    Modified Rankin Scale (mRS)    Barthel Index or FIM    LiSat-11 (Life Satisfaction Questionnaire)    Structured interviews (6- and 12-month follow-up) | 6 months and 12 months post-discharge | RTW at 6 months: 18%, at 12 months: 20%    Significant predictors of RTW:    Age (p = 0.009 at 6 months; p = 0.048 at 12 months)    Education (p = 0.04 at 6 months)    mRS score (p = 0.001 at 6 months)    Country differences (p = 0.03 at 12 months)    RTW negatively correlated with LiSat-11 vocational satisfaction : ρ = −0.31 at 6 months; ρ = −0.29 at 12 months | N/A | N/A |
| Emma Westerlind et al.[42] | - - **Design:** Retrospective cohort study using SALGOT-extended data (Sweden).   - **Participants:** Adults aged 18–63 years with first-ever stroke (2009–2010); follow-up at 5 years post-stroke.   - **Data collection:**     - RTW status from Swedish Social Insurance Agency.     - Perceived participation/autonomy via mailed IPA-E questionnaire (with reminders).     - Functional/medical data (NIHSS, mRS, GCS, Charlson Index) from medical records.   - **Analysis:** Logistic regression adjusting for age, sex, and disability level.   - **Focus:** Predictive relationship between RTW and long-term participation and autonomy. | Via mailed self-report questionnaire (IPA-E) at 5 years post-stroke; RTW status obtained from national registry | Individuals who had returned to work by 5 years post-stroke were significantly more likely to report high levels of perceived participation and autonomy across all domains of the IPA-E questionnaire.    Odds Ratios (OR) and significance values for each domain:   - Autonomy indoors: OR = 6.29, p = 0.003 - Family role: OR = 8.17, p = 0.001 - Autonomy outdoors: OR = 4.91, p = 0.005 - Social life and relationships: OR = 5.53, p = 0.002     The regression models showed moderate explanatory power, with Nagelkerke R² values ranging from 0.16 to 0.35.    ROC curve areas (AUC) for the models ranged from 0.71 to 0.80, indicating acceptable to good predictive accuracy. | RTW Linked to Higher Functional Status (mRS):  Although not the primary focus, the study confirmed that individuals who returned to work generally had lower levels of functional dependency at follow-up, as indicated by lower modified Rankin Scale (mRS) scores.    Sociodemographic Pattern of RTW:  RTW was more frequent in younger participants and in those with less severe strokes, although these were not tested as independent outcome variables in the final models.    Potential Role of Social Context and Identity:  The authors discuss that being employed may help maintain a sense of social role, structure, and identity, which could indirectly support higher self-rated participation and autonomy, though this was not directly measured. | Impact on Participation and Autonomy – English version (IPA-E)    NIH Stroke Scale (NIHSS)    Modified Rankin Scale (mRS)    Glasgow Coma Scale (GCS)    Charlson Comorbidity Index | 5 years post-stroke | Associations between RTW and all IPA-E domains:   - Autonomy indoors: OR = 6.29, p = 0.003 - Family role: OR = 8.17, p = 0.001 - Autonomy outdoors: OR = 4.91, p = 0.005 - Social life and relationships: OR = 5.53, p = 0.002     Nagelkerke R²: 0.16–0.35; AUC: 0.71–0.80 | N/A | N/A |
| Sophie Lehnerer et al.[43] | - - **Design:** Cross-sectional exploratory study (2017–2018) at Charité University Hospital, Berlin.   - **Participants:** First-ever ischemic stroke survivors (ICD-10: I63) assessed 2–3 years post-stroke.   - **Data collection:** Structured outpatient evaluations by neurologists and social workers.   - **Measures:** Nikolaus-score (social situation), EQ-5D (quality of life), mRS (functional status), Stroke Impact Scale (item 8), HPS-k (caregiver burden).   - **Intervention:** Individualized social work recommendations and referrals based on identified unmet needs.   - **Analysis:** Comparison of outcomes between patients with and without unmet social needs. | In-person structured assessments during outpatient follow-up visit | 35% of participants (20 out of 57) had unmet social needs, as indicated by a Nikolaus-Self-Sufficiency Score <17.    Patients with unmet needs had:   - Lower quality of life: EQ-5D index 0.70 vs. 0.89, p = 0.02 - More disability: mRS median score 3 vs. 2, p = 0.018 - Lower participation: Stroke Impact Scale item 8 score 21 vs. 34, p = 0.002     Those with unmet needs were more frequently recommended for social work support (57% vs. 28%, p = 0.035). | Among the 24 caregivers assessed, those supporting patients with unmet social needs reported higher levels of burden, as measured by the HPS-k.  Median HPS-k score was 21 in the unmet needs group vs. 6 in the met needs group (p = 0.053, trend toward significance).    Health System Gaps in the chronic phase, despite initial inpatient interventions.    Participants with unmet social needs were more frequently referred for additional social work support (57%) compared to those without unmet needs (28%), indicating clinical acknowledgment of persistent vulnerability.    The most frequent unmet needs were in the domains of mobility, household help, legal issues, and long-term care services.    There was a trend suggesting that patients with lower income and lower education had more unmet social needs, although not always statistically significant. | Nikolaus-Self-Sufficiency Score  It Measures:   - Mobility - Self-care (hygiene, dressing, eating) - Household activities - Cognitive aspects     EQ-5D-3L    Stroke Impact Scale (Item 8)    Modified Rankin Scale (mRS)    Caregiver burden (HPS-k) | N/A | Yes. Significant differences between those with and without unmet needs:    EQ-5D: 0.70 vs. 0.89 (p = 0.02)    Stroke Impact Scale: 21 vs. 34 (p = 0.002)    mRS: median 3 vs. 2 (p = 0.018)    Recommendation for social work: 57% vs. 28% (p = 0.035) | N/A | N/A |
| Neil Heron et al.[44] | - - **Design:** Randomized feasibility trial (SPRITE study) in Northern Ireland.   - **Participants:** Minor stroke or TIA survivors recruited from a hospital stroke unit.   - **Groups:**     1. Control (usual care)     2. Intervention (Healthy Brain Rehabilitation Manual)     3. Intervention (Manual + pedometer/Fitbit)   - **Intervention:** Six-week home-based program focusing on stroke education, lifestyle changes, goal setting, and mental well-being; GP follow-up calls at weeks 1 and 4.   - **Assessments:** Physical tests and standardized questionnaires at baseline and six weeks post-intervention. | Baseline and final assessment in hospital research facility    Intervention conducted at home    Follow-up support via telephone by GP at 1 and 4 weeks | Feasibility and adherence: The intervention was well accepted. Most participants completed the program and used the manual; some struggled with technology (Fitbit/pedometer).    Physical activity: Increase in average daily step count and improvement in IPAQ scores in intervention groups.    Functional capacity: Improvement in the 2-minute walk test performance in those receiving the intervention.    Mood: Improvements observed in Hospital Anxiety and Depression Scale (HADS) scores (reduction in anxiety and depression symptoms).    Quality of life: Slight improvements in EQ-5D-5L index scores in the intervention groups. | The pedometer and Fitbit were generally well received, but a few participants experienced technical difficulties or required assistance using them, highlighting a potential digital literacy barrier in older stroke patients.    The motivational interviewing approach used in GP follow-up calls appeared to support continued engagement and was positively evaluated in exit interviews.    Participants showed movement across Prochaska's Stages of Change, indicating improved readiness to maintain healthy behavior after stroke/TIA. | EQ-5D-5L    Hospital Anxiety and Depression Scale (HADS)    Modified Rankin Scale    2-minute walk test    International Physical Activity Questionnaire (IPAQ)    Mediterranean Diet Score    Prochaska Stages of Change    Blood pressure, heart rate, BMI, waist circumference | 6 weeks | N/A | - - Users expressed high satisfaction with the home-based rehabilitation program, finding the manual clear, informative, and practical.   - Goal-setting sections and lifestyle guidance were especially appreciated, contributing to a sense of motivation and empowerment.   - The pedometer/Fitbit helped raise awareness of physical activity, though some older participants needed assistance.   - GP-led follow-up calls were seen as encouraging and reinforced commitment to the program.   - Several participants suggested extending the program beyond six weeks to better support long-term secondary prevention. | N/A |
| Charlotte Wassenius et al.[45] | - - **Design:** Qualitative study embedded in the SAHLSIS cohort long-term follow-up.   - **Participants:** 9 stroke survivors (15–18 years post-stroke) who had returned to work; no major cognitive or language impairments.   - **Data collection:** In-depth, semi-structured interviews (in Swedish) at participants' preferred locations.   - **Focus:** Experiences of work, adaptation, identity, and participation over the long term after stroke.   - **Analysis:** Combination of individual narrative exploration and cross-case thematic analysis. | In-person interviews conducted at home, clinic, or significant other’s home    in what did consist the service that is being studied (describe only if mentioned in the article):  Services discussed included flexible return-to-work processes, workplace adjustments (tasks/hours), long-term occupational therapy, and psychosocial support related to work reintegration | Through thematic analysis, three major themes emerged:    **Work as identity and structure:**  Participants viewed work as central to their sense of self, contributing to purpose, pride, sense of belonging and social connectedness. Work provided daily structure and meaning, reinforcing a sense of normalcy.    **The process of regaining function through work:**  Work was perceived as a means of rehabilitation, helping participants to challenge themselves, regain confidence, and test their physical and cognitive limits in a meaningful context.    **Adjusting expectations and negotiating limitations:**  Participants described how they adapted to their new capacities by modifying work hours, tasks, and environments. This was often facilitated by understanding employers and flexible systems, but in some cases also involved loss of career ambitions or forced early retirement. | The presence of supportive employers and flexible working conditions (such as reduced hours or adjusted responsibilities) was essential for maintaining employment. Conversely, lack of workplace flexibility led to early retirement in some cases.    Many participants still experienced hidden disabilities like fatigue and concentration difficulties years after stroke. These residual symptoms influenced job retention and necessitated continued adaptation. | N/A | 15–18 years post-stroke as part of SAHLSIS study | N/A | Participants shared a wide range of experiences with the support services they received in their return-to-work journey. Overall, satisfaction was higher when services were individualized, continuous, and included psychosocial components, while gaps in long-term support were linked to frustration and the feeling of being left to compensate for systemic shortcomings on their own. | N/A |
| Veronica Ntsiea et al.[46] | - - **Intervention:** Tailored vocational rehabilitation including workplace visits, job demand analysis, and employer consultation.   - **Focus:** Align stroke survivors’ functional abilities with job demands and recommend workplace accommodations.   - **Components:** Individualized workability assessments, direct workplace engagement, and customized rehabilitation plans.   - **Follow-up:** In-person assessments at 3 and 6 months post-discharge.   - **Measures:** Mobility, cognitive function, quality of life, and return-to-work status; assessments conducted by blinded assessors. | Conducted through in-person assessments and workplace visits in a community-based outpatient setting | At 6 months, 60% of the intervention group had returned to work compared to only 20% in the control group (p < 0.001; Odds Ratio = 5.2).    Participants in the intervention group had higher functional scores on the Barthel Index and better mobility on the Modified Rivermead Mobility Index.    Cognitive function, measured by the Montreal Cognitive Assessment, was also significantly better in the intervention group at follow-up.    Those who returned to work reported higher quality of life, with better scores on the Stroke-Specific Quality of Life Scale (SSQoL).    The intervention was found to be significantly more effective in facilitating RTW than standard care alone. | The intervention group performed better on the Modified Rivermead Mobility Index, reflecting improved physical mobility post-stroke.  Association between cognitive/mobility scores and RTW:  Regression analysis showed that higher cognitive and functional scores were associated with higher likelihood of returning to work, reinforcing the interdependence of these variables.  These outcomes indicate that beyond facilitating return to work, the workplace intervention program also supported broader functional and psychosocial recovery in stroke survivors. | Barthel Index (BI)  Modified Rivermead Mobility Index (MRMI)  Montreal Cognitive Assessment (MoCA)  Stroke-Specific Quality of Life Scale (SSQoL), and a validated Return to Work Questionnaire | 3 months and 6 months | Difference in return to work at 6 months (60% intervention vs 20% control; p < 0.001, OR = 5.2)    Functional and cognitive scores also showed significant associations with return to work | The individualized approach, including workplace visits and coordination with employers, was described as effective and feasible, and the results suggest a high level of acceptability. | N/A |
| Moon KT, Jang W, Park HY, Jung M, Kim JB. [47] | - - **Design:** Single-subject experimental A-B-A design (South Korea).   - **Participants:** 3 individuals with post-stroke brain lesion-related disabilities (Grades 1–3); living at home; 12–33 months post-stroke.   - **Intervention:** Occupation-based community rehabilitation (CBR) program delivered at home by occupational therapists.   - **Components:** Task-oriented activities based on personal goals, home environment modifications, education on disease and resources, connection to local services. | Community-setting (through direct home visits by occupational therapists) | Modified Barthel Index (MBI) – ADL Independence  Participant 1: 49 → 72  Participant 2: 39 → 57  Participant 3: 52 → 66    EuroQol-5D (EQ-5D) – Health-Related Quality of Life  Participant 1: 0.396 → 0.796  Participant 2: 0.423 → 0.677  Participant 3: 0.466 → 0.701    Assessment of Motor and Process Skills (AMPS)  Motor Skills (logits):  Participant 1: 1.0 → 1.8  Participant 2: 0.2 → 1.8  Participant 3: 1.3 → 2.2    Process Skills (logits):  Participant 1: 1.0 → 1.8  Participant 2: 0.4 → 1.7  Participant 3: 1.2 → 2.0    According to the AMPS guidelines, a change of 0.5 logits or more indicates a statistically and clinically significant improvement. All participants exceeded this threshold in both motor and process domains. | During the A2 (withdrawal) phase, participants maintained or slightly improved their scores on the MBI, EQ-5D, and AMPS, indicating that the benefits of the intervention persisted at least 2 weeks after cessation.    Qualitative observations noted that participants reported greater engagement in personally meaningful activities (e.g., household tasks, personal hygiene routines).    The intervention led to practical modifications in the home that supported greater autonomy, such as reorganization of living spaces and installation of assistive tools.    The structured, individualized nature of the program seemed to foster greater confidence in performing tasks, as evidenced by steady improvements in AMPS process skills. | Modified Barthel Index (MBI)  EuroQol-5D (EQ-5D)  Assessment of Motor and Process Skills (AMPS) | 4 weeks | Modified Barthel Index (MBI) – ADL Independence  Participant 1: 49 → 72  Participant 2: 39 → 57  Participant 3: 52 → 66    EuroQol-5D (EQ-5D) – Health-Related Quality of Life  Participant 1: 0.396 → 0.796  Participant 2: 0.423 → 0.677  Participant 3: 0.466 → 0.701    Assessment of Motor and Process Skills (AMPS)  Motor Skills (logits):  Participant 1: 1.0 → 1.8  Participant 2: 0.2 → 1.8  Participant 3: 1.3 → 2.2    Process Skills (logits):  Participant 1: 1.0 → 1.8  Participant 2: 0.4 → 1.7  Participant 3: 1.2 → 2.0 | The authors report that all participants showed high adherence to the intervention sessions, and none dropped out throughout the 12-session program. | N/A |
| Ying-Tzu Tseng[48] | - - **Design:** Three-wave data collection at inpatient rehab start (baseline), pre-discharge, and one month post-discharge.   - **Data collection:** Structured interviews by trained interviewers; third wave via face-to-face or telephone.   - **Measures:** Demographics, rehabilitation potential (biological, psychological, social factors), and ADLs (Barthel Index).   - **Focus:** Impact of rehabilitation potential components (swallowing, muscle strength, motivation, cognition, social support) on post-discharge ADL outcomes. | Face-to-face interviews in hospital, telephone or face-to-face follow-up 1 month after discharge | Significant predictors of ADL included   - swallowing ability (β = 3.19, p < 0.001) - muscle power (β = 7.34, p < 0.001) - rehab motivation (β = 1.30, p < 0.001) - urinary incontinence (β = –15.49, p < 0.001) - cognitive function (β = –8.59, p < 0.01) | N/A | Barthel Index  Functional Oral Intake Scale  Rehabilitation Motivation Scale  Short Portable Mental Status Questionnaire  BSRS-5  Functional Social Support Scale | 1 month | Significant predictors of ADL included   - swallowing ability (β = 3.19, p < 0.001) - muscle power (β = 7.34, p < 0.001) - rehab motivation (β = 1.30, p < 0.001) - urinary incontinence (β = –15.49, p < 0.001) - cognitive function (β = –8.59, p < 0.01) | N/A | N/A |
| Clarke DJ, Powers K, Trusson D, Craven K, Phillips J, Holmes J, et al.[49] | - - **Design:** Mixed-methods process evaluation embedded in the RETAKE multicenter RCT.   - **Participants:** 58 stroke survivors, 22 occupational therapists (OTs), and 10 employers.   - **Intervention (ESSVR):** Stroke-specific vocational assessment, work preparation, job retention support, workplace liaison, employer education, fatigue management.   - **Delivery:** By trained OTs over 18 months, with additional mentoring.   - **Data collection:** Semi-structured interviews; analyzed via framework analysis.   - **Focus:** Fidelity, adaptations, implementation factors, and perceived impact of ESSVR. | Delivered by trained occupational therapists across 18 months    Highly individualized approach    Interviews conducted mainly via telephone and some face-to-face; intervention delivered in community and home settings | The ESSVR intervention was generally implemented with fidelity across sites, although some variations in delivery occurred due to contextual factors.    Participants reported that the intervention was helpful in addressing work-related concerns, building confidence, managing fatigue, and facilitating communication with employers.    Occupational therapists highlighted the importance of mentoring and specialized training for effective delivery. | Factors influencing the successful implementation of the ESSVR intervention   - Early timing - Therapist experience - Contextual support within stroke services.     Employers identified the intervention as useful in facilitating understanding of stroke-related limitations and promoting workplace accommodations. | N/A | 12 months | N/A | - - Stroke survivors were highly satisfied with the ESSVR intervention, praising its personalized, supportive, and empowering approach.   - Early support reduced anxiety about returning to work and helped rebuild confidence gradually.   - The holistic service addressed both physical and psychological challenges, including fatigue, emotional adjustment, and goal setting.   - Therapist involvement with employers eased the transition back to work and was seen as particularly helpful.   - The intervention was viewed as flexible, responsive, and crucial for promoting autonomy and vocational reintegration after stroke. | N/A |
| Katie E. Powers et al.[50] | - - **Structure of ESSVR intervention:** Four stages — early recovery, graded return to work (RTW), job retention, discharge; tailored to individual needs.   - **OT training and support:** 2-day initial training, 1-day refresher at 6 months, monthly mentoring sessions (~37.8 minutes/month).   - **Duration/frequency:** Case-specific; continued until discharge; no standardized timeline.   - **Mode of contact:** Home visits, employer communication, and liaison with stakeholders. | Delivered by sepcially trained Occupational Therapists (OTs).  Delivered at 16 NHS sites across England and Wales. OTs received structured training and mentoring | Return to Work (RTW) status – assessed 12 months post-randomisation. Defined as returning to any paid/unpaid work ≥2 hours/week. It was **positively associated with:**   - Higher fidelity (OR = 1.06, p = 0.01) - More years of stroke rehab experience (OR = 1.17, p = 0.03)     Fidelity of ESSVR delivery. Measured using a custom checklist from case records. Score: % of components delivered out of all eligible ones (range: 30.8%–100%). It **was positively associated with:**   - Monthly mentoring time (b = 0.29, p = 0.02) | **Mentoring** was the only OT factor significantly linked to fidelity  **No significant relationship** found for theoretical knowledge or education level  Highlights importance of **ongoing mentoring** in delivering complex rehabilitation | N/A | Primary outcome (RTW) assessed 12 months post-randomisation | RTW outcomes were positively associated with:  Higher fidelity (OR = 1.06, p = 0.01)  More years of stroke rehab experience (OR = 1.17, p = 0.03)    Fidelity was positively associated with:  Monthly mentoring time (b = 0.29, p = 0.02)    Mentoring was the only OT factor significantly linked to fidelity    No significant relationship found for theoretical knowledge or education level    Highlights importance of ongoing mentoring in delivering complex rehabilitation | N/A | N/A |
| Rosbergen ICM et al.[51] | - - **Intervention:** Enriched environment program — communal spaces, stimulating resources (books, games, iPads), structured mealtimes, group activities, and patient/family engagement.   - **Staff preparation:** Education sessions on enrichment principles and practices before intervention start.   - **Data collection:** Semi-structured face-to-face interviews with 10 staff members (7 nurses, 3 allied health professionals) conducted 8 weeks post-intervention.   - **Interview process:** Independent interviewer; hospital-based; audio-recorded and transcribed verbatim.   - **Analysis:** Thematic content analysis by three independent researchers. | The service was delivered by nursing staff and allied health professionals. Team champions coordinated implementation. Education sessions were conducted by the research team | Thematic findings (qualitative outcomes):    Theme 1: “The road to recovery has started” — observed increases in activity, psychological well-being, and family empowerment    Theme 2: “It takes a team” — importance of interdisciplinary collaboration, team dynamics, and workload issues    Theme 3: “Keeping it going” — sustainability challenges, routine change difficulty, contextual and environmental factors | Staff observed significant increases in physical, cognitive, and social activity among stroke patients    Intervention empowered patients and families, improved team dynamics, and shifted focus to recovery even in acute care    Structured components like communal meals were more effective than unstructured bedside stimuli    Staff identified barriers to sustainability: staff turnover, lack of space, inconsistent education, workload    Recommendations: embed enrichment into routine protocols, maintain education, use physical space wisely | N/A | Interviews conducted 8 weeks after intervention phase | N/A | Staff described patients as more alert, happier, more engaged    Communal activities seen as increasing normalcy and reducing loneliness    Teamwork enhanced, but staff turnover and weekend staffing posed challenges | Families felt more included, some hesitant at first    Positive reception to information materials and structured involvement |
|  |  |  |  | **DEMENTIA** |  |  |  |  |  |
| Catherine Quinn et al. [52] | 1. It aims to promote self-efficacy, independence, and coping skills through structured group sessions  2. It lasts 8 weeks, 1 session per week, 90 minutes per session  3. The sessions cover topics such as information about dementia, menaging memory difficulties, enjoying activities, coping skills, planning for the future and accessing local resources | 1. Face to face, group-based sessions held at an NHS memory clinic  2. Sessions facilitated by two trained NHS healthcare professionals: One staff nurse and one support work, with previous experience and also additional training specific to this intervention  3. Caregiver were involved in the first and the last sessions | - 1. Small positive effect on self-efficacy (primary outcome) at 3 month and maintained at 6 months.   2. Moderate improvement in capability-related quality of life (ICECAP-O) at 3 months and small effect at 6 months.   3. Reduction in depression symptoms at 6 months with a small effect size.   4. The program had no clear improvement on anxiety; anxiety scores were slightly higher in the intervention group at follow-ups (small effect).   5. Pilot study not powered for statistical significance — the results are based on effect sizes, showing preliminary but encouraging impact. | N/A | - 1. GSES (General Self-efficacy scale)- higher score means greater self-efficacy   2. HADS (Hospital Anxiety and Depression Scale)   3. CORE-OM (Clinical Outcomes in Routine Evaluation-Outcome Measure)   4. EQ-5D-3L (EuroQol Five Dimensions, Three levels)-it measures helath-related quality of life   5. ICECAP-O (ICEpop CAPability measure for older people) | 3 months post-randomization (at the end of the program)    6 months post-randomization (longer-term follow up) | N/A | - 1. Qualitative feedback through interviews at two months post randomization increased their confidence and sense of independence.   2. Allowed them to meet others with the same condition, which was perceived as comforting and supportive.   3. Helped them feel they were contributing to the group, not only receiving help.   4. Encouraged peer learning and reciprocity (sharing strategies and advice). | - 1. Positive behavioral changes in their loved ones (e.g., more communication, more initiative).      1. Enjoyment in discussions at home after the sessions.      2. Changes in their own supportive behavior (e.g., encouraging more autonomy for the person with dementia). |
| Clement Pigmouguet et al. [53] | - 1. Home-based occupational therapy (OT) consisting of 12–15 individualized sessions over 3 months, delivered by trained occupational therapists directly at the patient’s home   2. Focused on maintaining cognitive and functional abilities, adapting the home environment, and teaching compensatory strategies to support daily activities.   3. Included caregiver training and support, helping caregivers develop effective supervision, problem-solving, and coping skills.   4. Part of the French public health system (French Alzheimer Plan), designed as a non-pharmacological, community-based intervention for people with dementia. | Individual, face to face occupational therapy sessions at the patient's home | Significant reduction in neuropsychiatric symptoms, caregiver burden, and informal care time during the intervention.  Functional status stable during intervention but declined after.  Cognitive performance remained stable | N/A | - 1. MMSE(Mini-Mental State Examination) to measure the cognitive performance   2. DAD(Disability Assessment in Dementia) to measure the functional status   3. NPI(Neuropsychiatric Inventory) to measure the neuropsychiatric symptoms   4. EQ-5D VAS to measure the patient's quality of life   5. ZARIT SCALE-to measure the caregiver burden   6. RUD(Resource Utilization in Dementia) | 3 month follow up at the end of the 12-15 occupational therapy sessions  6 month follow up conducted 3 months after the intervention ended | N/A | No qualitative experiences | No qualitative experiences |
| A. Marijke van Haefen-van Dijk et al. [54] | CO day care   1. Delivered in community locations (e.g., cultural centers, community buildings). 2. Offers combined support for people with dementia and their informal carers. 3. Based on the adaptation-coping model, promoting emotional adjustment and self-management. 4. Includes peer support groups, educational sessions, social activities, and individualized care plans. 5. Encourages social integration and collaboration with local welfare organizations.     NH day care   1. Delivered within nursing home facilities. 2. Provides structured daytime activities for people with dementia. 3. Focuses mainly on respite care for caregivers and basic engagement for patients. 4. No formal carer support program or integration with community services. 5. Less personalized and socially integrated than the community model. | CO DAY CARE: delivered within cultural centres, community halls, or public venues, face to face support with structured activities for both patients and carers.    NH DAY CARE: institution based, focused on respite and patient activities only | - 1. Care needs (CANE)   2. Behavior and mood problems(NPI-Q)     No significant overall difference between groups, but important subgroup effects:   - 1. Participants in the CO day care showed reduced neuropsychiatric symptoms   2. Carers in the CO centers reported fewer care needs.   3. Greatest benefit observed in people with dementia living with their caregivers. | Quality of Life(QoL-AD) | - 1. CANE-Camberwell Assessment of need for the elderly   2. NPI-Q-neuropsychiatric Inventory Questionnaire   3. QoL-AD- Quality of Life in ALzheimer's disease Scale | Follow-up assessments at 3 and 6 months after baseline | N/A | No qualitative experiences | No qualitative experiences |
| Mayumi Sakamoto et al.[55] | - 1. Individualized music intervention delivered one on one to elderly individuals with severe Alzheimer's disease, using music personally meaningful to each patients. Conducted one-on-one in group homes and a long-term dementia hospital in Kobe, Japan. Sessions were held in quiet, familiar rooms to reduce external stressors and facilitate emotional engagement. Each participant received 10 sessions, lasting 30 minutes each, once per week over 10 weeks. The music was personally selected based on each participant’s life history and emotional associations.   2. Passive music group: Participants listened to their personalized music without interaction.   3. Interactive music group: Participants engaged in clapping, singing, and dancing, guided by trained facilitators who responded to their emotional cues. Sessions were facilitated by trained music therapists, occupational therapists, and nurses, all of whom received specialized training to implement the intervention and monitor participants' responses. | - 1. Individual face-to-face sessions in group homes and a dementia care hospital;   2. Delivered by trained facilitators as either passive (listening) or interactive (active engagement) music therapy. | - 1. Emotional state (Faces Scale)   2. Stess levels-measured trough autonomic nervous system indices: Heart Rate(HR), High Frequency(HF) component of heart rate variability(HRV)   3. Behavioral and Psyhological Symptoms of Dementia(BPSD)-measured using the BEHAVE-AD Rating Scale including: Paranoia, delusions, agression, activity disturbances, affective symptoms, anxieties and phobias     **SHORT TERM EFFECTS**(immediately after sessions):   - 1. Both passive and interactive music interventions significantly improved emotional state (Faces Scale) and induced parasympathetic dominance (stress reduction), compared to the control group (p < 0.01).   2. The interactive group showed greater emotional improvement and stronger autonomic effects than the passive group (p < 0.01).   **LONG TERM EFFECTS**:   - 1. BEHAVE-AD scores improved significantly in both intervention groups after 10 sessions   2. Passive group: reduced affective disturbance and anxieties/phobias (p < 0.025).   3. Interactive group: significant reductions in five BPSD domains: Paranoid/delusional ideation and Aggression | N/A | - 1. Faces Scale   2. Heart rate(HR)   3. High frequency (HF)   4. Component of heart rate variability(HRV)   5. BEHAVE-AD | Follow-up conducted 3 weeks after the final intervention session to assess sustained effects on BPSD | **SHORT TERM EFFECTS**(immediately after sessions):   1. Both passive and interactive music interventions significantly improved emotional state (Faces Scale) and induced parasympathetic dominance (stress reduction), compared to the control group (p < 0.01). 2. The interactive group showed greater emotional improvement and stronger autonomic effects than the passive group (p < 0.01).   **LONG TERM EFFECTS**:   1. BEHAVE-AD scores improved significantly in both intervention groups after 10 sessions 2. Passive group: reduced affective disturbance and anxieties/phobias (p < 0.025). 3. Interactive group: significant reductions in five BPSD domains: Paranoid/delusional ideation and Aggression | No qualitative experiences | No qualitative experiences |
| Dorota Szczesniak et al. [56] | - 1. Combined support model for both people with dementia and their informal caregivers, offered in the same community setting.   2. Activities for people with dementia include: Emotion-oriented care, Recreational and creative activities, Psychomotor therapy, Reminiscence sessions, Cognitive stimulation exercises.   3. Activities for caregivers include:Psychoeducational meetings (e.g., on dementia symptoms, treatment, ethical/legal aspects), Peer discussion/support groups, Personal consultation hours with staff, Joint family-staff meetings to express needs and discuss support.   4. MCSPs are located in accessible, familiar environments (e.g., community centers), not hospitals or medical buildings.   5. Close coordination with local health services, social care, and voluntary organizations. Activities are adapted to individual needs, preferences, and stages of dementia.   6. Users can self-refer, especially in Italy and Poland, increasing accessibility. | Face to face, group and individual sessions in accessible community settings | - 1. Improvement in the care pathway to day care activities for people with dementia and their caregivers after implementing MCSP      - It is assessed through: Comparison of the pathways before and after MCSP implementation in each country.      - Data collected via: Expert panels, standardized mapping templates, descriptive comparison of referral systems, access points and service integration.   2. Accessibility to day care activities> Measured through self-referall rates, referall sources, presence of waiting lists.   3. Service coordination: measured through: degree of integretion with GPs, municipalities, NGOs.   4. Carer support availability: measured through: Existence and structure of psychoeductaion and peer groups.   5. Public awarness and stigma reduction: measured through: expert impression, observed community engagement.   6. Systemic barriers of facilitators: measured through: expert discussion and national comparison     RESULTS  1. Improved access to day care services in all three countries, especially for patiets with mild to moderate stages.  2. Increased self-rederral rates notably in Italy and Poland.  3. Greater community integration and reduced stigma.  4. Stronger collaboration among local health, social, and volonaty organizations.  5. Enhanced early intervention, addressing care needs earlier in the dementia trajectory, before institutionalization.  6. More structured and and coordinated pathways in regions where dementia services were previosly fragmented or limited. | N/A | N/A | Pre- and post-implementation comparison: follow up at 9 months after MCSP launch(not individual-level longitudinal follow-up) | N/A | N/A | Caregivers experiences reported via expert panels: Highlighted improved access, reduced stigma and emotional support through MCSP |
| Shizuko Omote et al.[57] | - 1. Initiated within the workplace, often when supervisors or coworkers notice behavioral or performance changes (e.g., forgetfulness, errors, confusion).   2. Employers sensitively recommend medical evaluation, often involving the workplace nurse or occupational physician.   3. After diagnosis, the employee is invited to disclose their condition voluntarily (not forced), and a support plan is co-developed with management and often with family involvement.   4. Work tasks are adapted, schedules are adjusted, and the employee is supported in continuing to work in a way that preserves their autonomy and strengths. | It focuses on adaptive, preventive and supportive strategies implemented by non-clinical staff (supervisors, HR managers, company nurse) | To explore and describe how employers support employees with youn-onset dementia in workplace settings. Measured through 10 semi-structered interviews.  Results:  The study provides valuable insight into how workplaces can support employees diagnosed with young-onset dementia (YOD) and highlights the practical, emotional, and structural factors that contribute to successful continued employment.    It promotes dignity, early intervention, and task adaption. Informs future training and policy development for workplace inclusion | N/A | N/A | No follow up period | N/A | N/A | Employers described confusion before diagnosis, sensitivity in recommending medical consultation and post-diagnosis workplace adjustments trough task adaption, family collaboration and social support. Focus was on preserving dinity and ensuring safety while enabling continued emplyment. |
| Annelies van Rijn et al.[58] | - 1. People with dementia are supported to volunteer in community settings that match their skills and interests.   2. Each participant is guided by a project leader from the Meeting Center.   3. The goal is to foster self-worth, autonomy, and visibility in society.   4. The service is individualized and non-medical, designed to complement other forms of dementia care.   5. The service is supported by local governments and embedded in the public care infrastructure​ | Delivered through meeting centres and local community organizations(school, shops, green spaces). | - 1. Feasibility of implementation-measured trough stakeholder interviews on barriers/facilitators across 3 phases: preparation, starting, continuation   2. Stakeholder perceived benefits for participants(empowerment, well-being, inclusion)   3. Organizational factors(financial and human resources, cultural barriers)     Results:   - 1. Demonstrated that DemenTalent is feasible and well-aligned with public care goals in the Netherlands.   2. Helped increase social participation among people with dementia and strengthened community awareness.   3. Empowered people with dementia by allowing them to contribute meaningfully to society.   4. Showed that organizational commitment, public funding, and regional openness to dementia are key to success | N/A | N/A | Interviews were conducted 13 to 32 months after implementation began. Covers three phases: preparation, startup and continuation. | N/A | Only one interview with a project ambassador, who can also be called as a patient. He spoke about the positive emotional and personal impact of being involved in meaningful activities:   - Felt valued and useful - Regained a sense of self-worth and confidence - Participated in public discussions and events to reduce stigma around dementia | Stakeholders reported strong emotional and social benefits of DemenTalent for participants and families. Key facilitators included local leadership, flexible community partners and caregiver support. Barriers included staff capacity, funding insecurity and lack of dementia awarness among external organziations. |
| Johannes Oesterholm et al. [59] | - 1. OTs use both formal and informal tools to assess cognitive function and functional needs, often adapting their approach to minimize patient distress or stigma.   2. OTs help individuals maintain engagement in household, leisure, and social activities, focusing on preserved abilities and avoiding passivity.   3. Therapists rely on input from care staff, especially for advanced dementia cases, while also being critical of unqualified assumptions made by others.   4. OTs must balance self-determination of the person with dementia with caregiver requests, often acting as intermediaries in emotionally and ethically complex situations.   5. A strong focus is placed on “saving face” during assessments—preserving the individual’s confidence and identity while gently confronting limitations. | Provided by licensed occupational therapists in face to face settings | To explore occupational therapists' real-world experiences working with people with dementia. Five main themes emerged:   1. Working with persons who do not recognize their decline 2. Saving face in assessment situations 3. Facilitating continued engagement in daily activities 4. Using information from care staff 5. Balancing the preferences of the person with dementia and significant others   Results:  Highlights the emotional complexity and ethical challenges OTs face when working with people with dementia.  Shows how therapists adapt communication and assessment strategies to promote person-centered, dignity-preserving care.  Emphasizes the importance of enabling rather than replacing the person’s participation in daily life.  Suggests the need for increased awareness and training on navigating family dynamics, system-level constraints, and dementia-specific care. | N/A | N/A | N/A | N/A | N/A | N/A |
| Justin Chew et al. [60] | - 1. Individualized goal setting using Goal Attainment Scaling (GAS) at baseline   2. Group-based rehabilitation, combining physical, cognitive, and social activities Weekly sessions over 8 weeks, supervised by multidisciplinary team   3. Progress tracking and home reinforcement, with feedback to participants and caregivers   4. Emphasis on shared decision-making and empowerment, including caregivers in planning   5. Sessions include:      - 45 minutes of multicomponent physical exercise      - 1 hour of cognitive stimulation (e.g., reminiscence therapy)      - 30 minutes of individualized, person-centered activity   6. Delivered by a multidisciplinary team: nurse coordinator, physiotherapists, occupational therapists, psychologists | Once-weekly group sessions for 8 weeks, each lasting 3 hours | - 1. Goal attainment Scaling(GAS): indivudalized goal-based measure   2. Cognition (CMMSE)   3. Functional performance (IADL)   4. Behavior(NPI-Q)   5. Quality of Life(EQ-5D)   6. Caregiver burden(ZBI)     RESULTS:   - 1. 62% of participants achieved or exceeded personal goals   2. Cognitive, functional, and behavioral scores remained stable, but individual goals (especially socialization, caregiver burden, and mood) were frequently met   3. GAS was strongly associated with improvements in caregiver burden   4. Showed that goal-oriented, individualized rehabilitation can yield meaningful outcomes even when conventional metrics are unchanged   5. Highlights the value of personalized care and multimodal interventions in dementia management   6. Reported feedback indicates positive experiences in empowerment, confidence, and reduced stress | N/A | - 1. GAS-goal attainment Scaling   2. CMMSE-chinese Mini-mental state examination   3. Barthel Index IADL-Instumental acitivities of daily living   4. EQ-5D-EuroQol-5D   5. ZBI-zarit burden Interview | Conducted at 8 weeks(at the end of the program) | N/A | N/A | N/A |
| Georgina Charlesworth et al.[61] | **CSP (Carer Supporter Programme):**   - Delivered one-on-one by trained former carers (volunteers) - Important for emotional support, not formal advice - 12 weekly sessions + 5 months of follow-up (22 hours total) - Meetings took place in homes, cafes, or via telephone     **RYCT (Reminiscence Therapy):**   - 12 weekly group sessions (2 hours each) + monthly sessions for 7 months - Included joint activities, such as storytelling, singing, object handling, and small group discussions. Using lifespan themes and multisesory prompts - 4 sessions included separate carer education groups All groups continues to receive standard community dementia support | The peer support is delivered by former carers to current family caregivers. It is provided face to face or by telephone | - - For carers is valutated the impact on the emotional well being (SF-12)   - For persons with dementia the impact on the quality of life (QoL-AD)     No significant benefit from CSP or RYCT on primary outcomes (SF-12 or QoL-AD) | - - Quality of relationship   - Caregiver burden   - Functional capacity   - Emotional well-being   - Self-related health     Some small but significant effects on carer–person relationship quality (QCPR), especially for CSP (p = 0.05) | - - SF-12- mental component Score mental   - HRQoL QoL-AD-(Quality of life-Alzheimer's disease)   - QCPR(Qulity of caregiver-patient relationship)   - Caregiver burden: NPI-D, COPE, EQ-5D, HADS, PANAS, PAC, PGI HADS   - Emotional well-being EQ-5D   - VAS for the self-rated health | Follow-up at 12 months | - 1. No significant benefit from CSP or RYCT on primary outcomes (SF-12 or QoL-AD)   2. Some small but significant effects on carer–person relationship quality (QCPR), especially for CSP (p = 0.05)   3. Combined intervention (CSP + RYCT) showed no additive benefit   4. Findings contradict popular belief and clinical guidelines, which often endorse reminiscence and peer support   5. Highlights a disconnect between subjective appeal and measurable impact of such interventions      - “The benefits of widely recommended interventions should not be assumed. We found no statistically significant improvements in quality of life or mental health, but the carer–recipient relationship quality did improve modestly. This suggests targeted, relationship-based interventions may merit further investigation.” | N/A | N/A |
| Helen Tam-Tham et al. [62] | Services were grouped into **7 main themes,** including:   - Medical assessment and treatment - Future planning (e.g., advance care planning, power of attorney) - Educational/social support (Alzheimer Society, caregiver education) - Home care and in-home support - Day programs (for respite and cognitive stimulation) - Safety and emergency services (e.g., fall prevention, wandering) - Transition to long-term care and end-of-life care     Timing of service delivery varied significantly by location  Calgary providers more frequently accessed early planning services  Ottawa providers were less likely to refer to day programs at the recommended time  Services were delivered through:Public health programs, Community agencies (e.g., Alzheimer Society), Non-profits and municipal home care systems | - - Healthcare providers (GPs, specialists, case managers) were interviewed using a 13-point vignette simulating disease progression   - They indicated what services they would recommend or access at each stage   - Data reflected real-world clinical decision-making and service availability | - 1. Provision of guideline-concordant dementia services across disease stages. Assessed by matching provider responses to Canadian Consensus Guidelines   2. Regional variability in service delivery.   3. Differences by provider type (GPs, specialists, case managers)     Results:   - 1. Revealed substantial regional variability in timing and access to dementia-related services, despite universal healthcare   2. Showed early future planning and day program referrals were underutilized in Ottawa and Edmonton compared to Calgary   3. Highlights gaps between national care guidelines and practice   4. Suggests need for:      - Better clinical pathways      - Professional education      - System-wide standardization      - Policy-relevant: informs the development of a national dementia strategy and regional resource alignment | N/A | - - Chi-square   - Fisher's exact tests | N/A | N/A | N/A | N/A |
| Carina Wattmo et al. [63] | - 1. Home-help services provide non-clinical daily support (e.g., meal prep, dressing, bathing).   2. Nursing homes offer 24-hour care and are a final step in dementia progression.   3. Services are part of Sweden’s public health system, with access based on functional needs.   4. Usage and hours/week of service recorded every 6 months over 3 years.   5. Predictors of service use and placement (e.g., IADL, PSMS scores) were analyzed. | **Home-help services**   - Provided in the person’s home by public sector caregivers - Include: hygiene, domestic tasks, assistance with daily routines - Recorded in hours/week by dementia nurses during 6-month follow-ups     **Nursing home care**   - 24-hour care facilities (excludes respite or rehab care) - Entry tracked during the 3-year study period | - 1. Cognitive status   2. Funtional ability-IADL   3. Medication burden     RESULTS   - 1. Cognitive ability was not a predictor of service use—suggesting services were not well-aligned with cognitive decline   2. For solitary-living individuals:      - Lower IADL ability and more medications predicted home-help use      - Impaired IADL and faster decline predicted nursing home placement   3. For those living with family:      - Female sex, older age, basic ADL impairment, and high medication use predicted service use      - More hours of home help predicted faster nursing home entry      - Policy relevance: Highlights a mismatch between needs and service provision in cognitively impaired individuals who live alone   4. Advocates for tailoring community-based services to better address cognitive impairment and risks in solitary-living dementia patients | N/A | - 1. MMSE(mini-mental state exam)   2. IADL(instumental activities of daily living scale)   3. PSMS (Physical self maintenance scale)   4. Total number of medications | Every 6 months for 3 years | N/A | N/A | N/A |
| Rose-Marie Droes et al.[64] | - 1. Customized modules to address individual needs in coping, daily function, and emotional adjustment   2. Delivered by trained professionals (psychologists, occupational therapists, coordinators)   3. Services designed to support both persons with dementia and their caregivers together   4. The digital component (dem@entia) supported ongoing education and peer interaction   5. Care was structured, manualized, and integrated into the Dutch public social care framework iMCSP-Expanded version of the standard Meeting Center model, including:      - Dem@entia digital platform (psychoeducation and peer support-provides reliable information on dementia, care strategies and emotional support tools, includes forums, eductaional materials and peer interaction)      - Act-in-ADL program (ADL training for people with dementia and caregivers-aimed at enhancing indipendence in daily tasks like washing, dressing, eating, housekeeping tasks)      - STAR program (emotional coping and stress reduction-help caregivers develop emotional coping strategies and communication skills)   Compared to regular MCSP, which includes general psychosocial and recreational activities | - 1. Delivered by a trained interdisciplinary team in local Meeting Centers   2. iMCSP users choose from three modules based on personal needs and goals   3. Sessions included:      - Group activities      - Online peer education (dem@entia)      - Role-play and communication support (STAR)      - Task-based ADL training (Act-in-ADL)   4. Participants attended 2–3 times/week, for 6 months | - 1. Behavioral symptoms(NPI-Q)   2. Mood(CSDD)   3. Sense of competence of the carers(SSCQ)   4. Quality of life(QoL-AD)     Results:   - 1. People with dementia in the iMCSP group showed significantly fewer behavioral problems at 6 months compared to MCSP(p = 0.046)   2. Caregiver Competence (SSCQ): Carers in the iMCSP group experienced a greater improvement in sense of competence(p = 0.027)   3. Among iMCSP participants:      - STAR program: 91% reported positive effect on understanding emotions.      - Act-in-ADL: 89% reported feeling more competent in supporting everyday activities.      - dem@entia: 84% found the information practical and relevant   4. No statistically significant difference in QoL-AD between groups at follow-up. However, self-rated QoL remained stable in the iMCSP group, while proxy-rated QoL declined slightly in MCSP. | - 1. Utiliaztion(which modules were used, frequency)   2. Subjective benefit(measured via questionnaire developed for this study)   3. Drop-out and attendance rates | - 1. **NPI-Q** (Neuropsychiatric Inventory – Questionnaire)   2. **Cornell Scale for Depression in Dementi**a (CSDD)   3. **SSCQ** (Short Sense of Competence Questionnaire)   4. **QoL-AD** (self- and proxy-rated) | 6 month follow up | - 1. People with dementia in the iMCSP group showed significantly fewer behavioral problems at 6 months compared to MCSP(p = 0.046)   2. Caregiver Competence (SSCQ): Carers in the iMCSP group experienced a greater improvement in sense of competence(p = 0.027)   3. Among iMCSP participants:      - STAR program: 91% reported positive effect on understanding emotions.      - Act-in-ADL: 89% reported feeling more competent in supporting everyday activities.      - dem@entia: 84% found the information practical and relevant   4. No statistically significant difference in QoL-AD between groups at follow-up. However, self-rated QoL remained stable in the iMCSP group, while proxy-rated QoL declined slightly in MCSP. | **Act-in-ADL module:** Many PwD said they felt more independent and useful in daily life. Expressed satisfaction in being able to "do more things at home" with less reliance on caregivers.    **STAR module**:Participants shared that they became more aware of their emotional responses. Gained confidence in expressing themselves and felt less misunderstood.    **dem@entia platform:**Those who used it appreciated access to trustworthy information, especially in early-stage dementia. Felt more "in control" of their condition.    **Overall experience:**  PwD appreciated that the service was tailored to their specific goals, not a “one-size-fits-all” activity. | **Act-in-ADL:** Carers reported learning concrete skills to help their loved one maintain independence (e.g., step-by-step prompts). 89% reported feeling more effective and less stressed during ADL support tasks.    **STAR module**: Caregivers valued learning how to better manage their own emotions and respond to emotional outbursts. 91% said it helped them understand the emotional world of the person with dementia.    **Overall experience:**   1. Many appreciated the structure, personalization, and interdisciplinary support of iMCSP. 2. Several expressed feeling less isolated and more equipped to navigate caregiving over time. 3. One commonly cited benefit was the flexibility to choose a module that fit their current needs. |
| Netta Van't Leven et al.[65] | Three Interventions Studied:  1. Pleasant Events Program  2. Exercise and Support Intervention  3. Community Occupational Therapy for Dementia  Common elements:   - Delivered at home include: activity training, psychoeducation, and emotional support - Tailored to dyads' personal needs   Working Mechanisms Identified:  Empowerment as the core mechanism. Achieved through:   - Enabling activities without false hope - Deep exploration of dyads' activity needs - Solution-focused adaptation of daily routines - Included goal setting, activity reharsal, needs assessment and homework tasks | Face-to-face, in-home sessions by trained coaches (occupational therapists, psychology students, home care workers) | No quantitative outcomes; qualitative outcomes based on participants' perceived changes on:   - - Empowerment   - Self-efficacy   - Emotional relief   - Re-engagement in meaningful activities   Main focus on:   - - Improved activity engagement   - Better coping strategies   - Positive attitude shift     Results:   - - Demonstrated that empowerment is the primary working mechanism.   - Showed that tailored, home-based dyadic interventions:     - Increase confidence     - Improve daily functioning     - Promote sustainable coping strategies   - Supported broader use of personalized training approaches over generic care | N/A | N/A | Some interviews ocurred 6-9 months after the intervention for reflective input | N/A | - 1. Enhanced sense of autonomy and control- PwD appreciated being involved in choosing and doing meaningful activities.   2. Increased confidence in daily tasks-They reported feeling more capable and less afraid to try things independently.   3. Reduction in emotional distress-Participation in adapted routines reduced frustration, confusion, and conflict.   4. Positive emotional shifts-PwD described feeling calmer, more hopeful, and more connected to their caregivers.   5. Valued being acknowledged as capable-Felt respected and seen as partners in the intervention process.   6. Enjoyment and pleasure from re-engagement-Described the activities as enjoyable and energizing. | 1. Improved caregiving confidence-Caregivers felt more capable of responding to difficult behaviors and task-related challenges.  2. Practical skills for daily routines-Gained tools to adapt communication, initiate tasks, and maintain calm interactions.  3. Decreased emotional burden-Many reported feeling less overwhelmed, less anxious, and more emotionally balanced.  4. Renewed partnership with the PwD-Felt the intervention promoted shared responsibility and mutual engagement.  5. Better understanding of dementia’s effects-Psychoeducation and coaching helped them make sense of symptoms and adapt expectations.  6. Appreciation for structured support-Valued the coach’s professional input and the safe space for discussing struggles |
| Samira Sangi et al.[66] | Multimodal cognitive rehabilitation program including:  1. Cognitive exercises (e.g., memory, orientation, verbal comprehension)  2. Neuromuscular exercises (e.g., walking, ball activities, balance)  3. Emotional training (e.g., empathy, motivation, coping, acceptance) | - - The intervention administered by trained researchers or therapists as part of a structured program within the nursing home.   - Duration of the service: 8 weeks with 18 sessions total.   - Frequency of the service: 2-3 times per week, 1-hour sessions | - 1. Cognitive function   2. Depression   3. Daily functioning     Results:  **Cognitive function:**  Experimental group showed significant improvement on MMSE: Post-test MMSE mean = 27.86 vs. pre-test = 25.53    **Depression:**  GDS scores decreased in the experimental group: Post-test mean = 2.93 vs. pre-test = 5.47    **Daily functioning (Barthel Index):**  Post-test mean = 95.13 vs. pre-test = 91.07  → Indicates improved independence in daily living  **Statistical significance:**   - - MMSE: F = 24.30, p ≤ 0.01   - Depression: F = 17.67, p ≤ 0.01   - Barthel Index: F = 29.78, p ≤ 0.01   Effect size (partial eta squared):  η² = 0.524, meaning the intervention explained 52% of the variance in functional improvement. | N/A | - - MMSE-Mini mental state examination for the cognitive function   - GDS-geriatric depression scale for the depression   - Barthel index- for the daily functioning | Immediate post-intervention only | **Statistical significance:**   - MMSE: F = 24.30, p ≤ 0.01 - Depression: F = 17.67, p ≤ 0.01 - Barthel Index: F = 29.78, p ≤ 0.01   Effect size (partial eta squared):  η² = 0.524, meaning the intervention explained 52% of the variance in functional improvement. | N/A | N/A |
| Janne Rosvik et al.[67] | - 1. Type of service: Implementation of a person-centred care model (VIPS) in primary health care settings for people with dementia.   2. Setting: Community-based settings, including domestic nursing care and long-term institutional care.   3. Duration: The model had been in use for at least 12 months in each setting included in the study.   4. Frequency: Weekly consensus meetings are the model’s central component.   5. Additional details: Training includes a 2-day course for staff, and implementation is supported by manuals, posters, and internal supervision.   6. Delivery: Entirely integrated into daily clinical routines. | The VIPS practice model is delivered primarily by:   - Registered nurses - Auxiliary nurses - Care assistants - Managers and head nurses who organize and sustain the weekly consensus meetings central to the model     The staff who implement VIPS typically:   - Undergo a 2-day training course before starting - Use a structured meeting guide, posters, and documentation templates - Are supported by internal leaders or champions responsible for coordinating VIPS in each unit | - 1. Identification of barriers and facilitators in the implementation of the VIPS practice model.   2. Understand how leadership, training, and organisational structure influence success.     Results:   - 1. Improved quality of dementia care: Staff noted enhanced awareness and person-centred focus in day-to-day decisions.   2. Increased staff collaboration: Weekly VIPS meetings fostered shared decision-making and interprofessional dialogue.   3. Reduced misunderstandings and unnecessary medication use: Staff reported improved handling of BPSD (behavioral and psychological symptoms of dementia).   4. Empowered staff: Caregivers felt more confident and validated in their observations and actions.   Structural change: Some organizations began to restructure routines around VIPS, making it part of the standard workflow   - 1. Challenges to sustainability: High turnover, competing initiatives, lack of administrative support, and insufficient time were all noted as barriers. | N/A | N/A | Minimum 1 year follow up post-implementation | N/A | **STAFF EXPERIENCES**  Theme 1: "The VIPS model clarifies and structures dementia care". Staff valued the clarity of expectations and focus on personhood. VIPS helped them to document and reflect on care decisions more systematically.  One nurse said:“Before, I just noted things down. Now, I understand why I’m observing certain behaviors.”    Theme 2: "Leadership and continuity are critical"  Staff appreciated when leaders actively participated in VIPS meetings. Several said implementation failed in units where management wasn’t engaged.  Staff turnover negatively impacted continuity: “New staff don’t know what VIPS is… it gets lost when no one owns it.”    Theme 3: "Integration into daily routines is essential"  The model worked best when seen as part of clinical care, not an extra task. Staff said the model increased collaboration between roles, giving equal voice to assistants, nurses, and coordinators: “Now we talk about our patients together – we make shared decisions.” | N/A |
|  |  |  |  | **MULTIPLE SCLEROSIS** |  |  |  |  |  |
| Matthew Plow et al.[68] | The intervention consisted of two main components:   1. **Standardized home exercise program**  - Setting: Home-based, with two in-person sessions at the beginning - Content: Aerobic, balance, and strength training exercises using a mini-cycle and elastic bands - Frequency: 3–5 days per week - Duration: 12 weeks - Support: In-person instruction at baseline and 2 weeks later      1. **Customized pamphlet-based intervention**  - Content: Tailored print materials addressing: - Stage of change (Transtheoretical Model) - Common MS-related barriers to physical activity - Self-management strategies     Delivery: One pamphlet every 3 weeks  Supplemented by: Phone calls every 3 weeks from a trained instructor to discuss PA habits and tailor further content | - - Initial exercise training: First author (trained in MS rehabilitation)   - Pamphlet follow-up and phone calls   - Certified health and fitness instructor with training in exercise promotion for special populations   - No mention of clinicians directly delivering ongoing care | - 1. Stages of changes- measured with a stages of changes questionnaire   2. Behavioral processes of change   3. Social support   4. Self-management   5. self-efficacy   6. PA self-efficacy     Results:  Significant improvements in:   - - Stages of change placement (F = 16.64, p < 0.001)   - Behavioral processes of change (F = 9.07, p = 0.005)   - Social support from family (F = 4.08, p = 0.05)     No significant improvement in:   - - Physical activity self-efficacy   - Self-management self-efficacy | N/A | - 1. Stages of Change Questionnaire (1–5 scale from pre-contemplation to maintenance)   2. 40-item scale (behavioral and cognitive subscales, 1–5 scale)   3. Social Support for PA Scale (subscales: family and friends, 0–5)   4. Self-Efficacy for Managing Chronic Disease scale (0–10 scale)   5. Marcus PA Self-Efficacy Scale (1–5 scale; includes MS-specific item) | Short term follow up (post-intervention evaluation only, after 12 weeks) | Significant improvements in:   - Stages of change placement (F = 16.64, p < 0.001) - Behavioral processes of change (F = 9.07, p = 0.005) - Social support from family (F = 4.08, p = 0.05)     No significant improvement in:   - Physical activity self-efficacy - Self-management self-efficacy | Positive themes:   1. Felt that the pamphlets helped manage fatigue, a common MS symptom 2. Pamphlets provided simple, motivating, and consistent guidance 3. Phone calls increased accountability, especially when they came from a familiar instructor 4. Participants appreciated being treated as individuals, not just as patients 5. Many reported feeling more in control of their physical activity habits     Negative or improvement suggestions:   1. Some participants found the exercise program too challenging or repetitive 2. Others wanted more personalized materials, tailored to their physical capacity and interests 3. Visual layout and tone of pamphlets were criticized by a few as too simplistic or generic | N/A |
| Glattacker M. et al.[69] | - - The study focuses on patients' utilization of the standard healthcare services rather than a specific provider-delivered program.   - Setting: Rehabilitation services are offered in hospital-based outpatient rehabilitation centers and may also be available in specialized clinics.   - Duration: Rehabilitation episodes are variable in length, depending on clinical need; the study examines overall utilization rather than a fixed-duration program.   - Frequency: Frequency of use is measured as the number and regularity of rehabilitation sessions attended over a given period (assessed via self-report or clinical records). | Rehabilitation services in MS are delivered by multidisciplinary teams typically comprising:   - Physiotherapists - Occupational therapists - Speech and language therapists (where applicable) - Rehabilitation physicians | **Rehabilitation Service Use**   - - Assessed as the frequency and extent of rehabilitation service utilization (e.g., number of sessions attended, use vs. non-use of rehabilitation services) via self-report questionnaires and/or clinical record review.   - Illnes representations: IPQ   Results:   - - The study finds that certain dimensions of illness representations (for example, perceptions of personal control, consequences, or timeline of MS) are significantly associated with the use of rehabilitation services.   - Patients with more adaptive or positive illness beliefs are more likely to engage in rehabilitation.   The work suggests that understanding patients’ illness perceptions might help in designing strategies to improve rehabilitation uptake.   - - This study is important for tailoring rehabilitation outreach and for interventions aiming to modify illness representations in MS, thereby potentially enhancing service use and clinical outcomes. | N/A | IPQ- Illness perception questionnaire | N/A | N/A | **Patients’ experiences:** Some patients describe rehabilitation as beneficial in addressing physical and cognitive challenges; others note that their personal beliefs about the illness affect their willingness to participate.    **Barriers:** Common reported barriers include negative illness perceptions (e.g., believing that MS is uncontrollable or has severe consequences), which can deter rehabilitation use.    **Facilitators:** Patients who feel more in control or perceive a greater potential benefit are more likely to use rehabilitation services. | N/A |
| Niall Russell et al.[70] | - 1. The intervention is a group-based physical activity intervention underpinned by Social Cognitive Theory SCT.   2. It focused on behavior change strategies to increase physical activity in people wit MS, especially those who were physically inactive but ambulatroy.   3. Duration: 10 weeks   4. Frequency: Weekly group sessions combining physical activity (aerobic and strength exercises) with SCT-based education (e.g., discussions, videos, reflective tasks)   5. SCT content focused on improving self-efficacy, goal setting, and coping with barriers   6. All sessions delivered in person, with social support encouraged within the group | - - Physiotherapists delivered the group physical activity sessions   - SCT-based behavioral education was also provided by trained facilitators (part of the research team) | - 1. Beliefs about exercise and MS   2. Perceived barriers and facilitators   3. Transformation of self-identity   4. Motivation and self-efficacy   5. Social influence and peer comparison     The intervention positively shifted participants’ beliefs and behaviors:   - - Improved mood, energy, and motivation   - Increased self-efficacy and autonomy in managing physical activity   - Reduced anxiety and guilt related to inactivity   - Participants reported a redefined MS identity, viewing themselves as more capable and empowered   - Group dynamics (social support, peer modeling, shared goals) were crucial to success   - The role of the physiotherapist as a motivator was heavily emphasized     **Relevance:** This intervention model effectively addresses psychological and social barriers to physical activity in early-stage MS and could be adapted in other community-based programs | N/A | Semi-structured interviews | N/A | N/A | Positive impacts:   - Felt more in control of their MS and lifestyle - Reported less fatigue and emotional distress - Developed confidence and routine around exercise - Emphasized the emotional benefit of being part of a group, and not feeling "different"     Challenges noted:   - Some initially feared overexertion or symptom worsening - Concerns about maintaining motivation post-program | N/A |
| Andreas Falck Lahelle et al. [71] | Setting: Community-based rehabilitation delivered in municipal physiotherapy clinics. Structure:   - Group size: 3 patients per group - Duration: 6 weeks   Sessions per week:   - 3× 60-minute supervised sessions - 2× 30-minute unsupervised home-based sessions   Exercise structure:   - All participants perform the same type of exercises simultaneously, but at individually tailored levels of difficulty - PTs choose from a set of 33 predefined core stability exercises (each with 5 difficulty levels) | The service was delivered by: **Neurological physiotherapists**   - with experience in group and neurological rehab. - Some held master’s degrees and >10 years of experience. - Most worked in primary healthcare. | - 1. Group dynamics   2. Individual interaction with physiotherapists   3. Peer support     Results:   - 1. Individualization enhances group dynamics: PTs using patient-specific touch and verbal cues helped build motivation and team spirit   2. Positive group dynamics emerged when patients perceived personal success in movement tasks   3. Peer support (social + exercise-focused) arose naturally, especially when PTs encouraged sharing   4. Barriers to success: Lack of individualized attention led to disengagement; wide functional level variation within groups sometimes hindered motivation   5. Key message: Individualized care within group settings is feasible and beneficial, contrary to traditional belief that they are mutually exclusive | N/A | Assessment method:   - Non-participatory video recordings of sessions - Thematic interviews with PTs - Systematic text condensation used to extract patterns in behavior, communication, and movement interactions | N/A | N/A | Positive experiences:   - Patients appreciated individualized attention within a social group setting - Felt increased motivation, peer bonding, and emotional support - Shared experiences (e.g., medication challenges, early diagnosis fears) created a supportive micro-community     Exercise-specific interactions:   - Patients gave each other real-time encouragement and shared feedback - Noted improvements in balance, confidence, and sense of progress - Valued that the PTs encouraged open discussion and reflection | N/A |
| Evan Mansson Lexell et al.[72] | - - Duration: 2 to 12 weeks (mean = 4 weeks)   - Frequency: Daily sessions including multiple types of rehabilitation services   Services included:   - - Physical and occupational therapy   - Water and land-based exercise   - Social/psychological counseling   - Assistive device prescription   - Training in self-care, household management, work, and leisure activities   - Regular goal review using the ICF framework     Assessment used: Canadian Occupational Performance Measure (COPM) on admission and discharge | The service was delivered by:   1. Rehabilitation physician 2. Physiotherapist 3. Occupational therapist 4. Social worker   All had experience in MS rehabilitation. | - - Self-perceived performance of daily activities   - Satisfaction with performance of daily activities       Results:  Improvement observed in:  1. Performance scores: 60% of activities  2. Satisfaction scores: 60% of activities  3. Clinically significant change (≥2.0 points):  4. Performance: 18 of 43 participants (42%)  5. Satisfaction: 24 of 43 participants (56%)  6. COPM area most improved: Self-care (personal care, functional mobility)    Implication:   - - Participants report substantial improvement after interdisciplinary rehab   - PROMs like COPM provide rich insight into functional change | N/A | - 1. Canadian Occupational Performance Measure (COPM)   2. Semi-structured interview on admission and discharge   3. Each activity rated (1–10) for both performance and satisfaction     Clinically significant change = ≥2.0 points | N/A | Improvement observed in:  1. Performance scores: 60% of activities  2. Satisfaction scores: 60% of activities  3. Clinically significant change (≥2.0 points):  4. Performance: 18 of 43 participants (42%)  5. Satisfaction: 24 of 43 participants (56%)  6. COPM area most improved: Self-care (personal care, functional mobility)    Implication:   - Participants report substantial improvement after interdisciplinary rehab - PROMs like COPM provide rich insight into functional change | N/A | N/A |
| Saba Yaseen Hyarat et al.[73] | No service delivered.   1. This is a non-interventional study. Key context elements include:  - Setting: Data collected in outpatient clinics of two public hospitals in Jordan - Duration: One-time data collection (March–July 2015) - Frequency: Single survey per participant using standardized tools     Patients completed:   - Demographics questionnaire - MSQoL-54 (health-related quality of life) - PAIS-SR (Psychosocial Adjustment to Illness Scale – Self Report) | N/A | - 1. Health-Related Quality of Life (HRQoL)-measured using the MSQoL-54 (includes physical and mental composite scores, plus subscales (e.g., pain, sexual function, energy, cognition)     Higher scores = better QoL | - 1. Psychosocial Adjustment to Illness:      - PAIS-SR (Psychosocial Adjustment to Illness Scale – Self Report)   Measures 7 domains: health care orientation, vocational environment, domestic environment, sexual relationships, extended family, social environment, psychological distress  Higher scores = worse adjustment   - 1. Maladjustment = total score ≥ 62 (65.6% of participants were maladjusted)   2. MSQoL-54: 54 items, scored 0–100   3. PAIS-SR: 46 items across 7 domains, Likert scale 0–3     Results:   - 1. Participants showed poor QoL, especially in mental health (mean score = 22.3)   2. Psychosocial adjustment difficulties were high; 65.6% showed clinical maladjustment   3. Strong negative correlation between maladjustment and both physical and mental HRQoL     **Significant predictors of poor HRQoL:**   - - Female gender   - Unemployment   - Poor income   - Lower education   - More frequent relapses     **Implication:**   - - Psychosocial adjustment is a critical determinant of QoL in MS   - Need for targeted psychiatric/psychosocial interventions in MS care   - Suggests shifting some MS care to primary care/mental health services | N/A | N/A | - 1. Participants showed poor QoL, especially in mental health (mean score = 22.3)   2. Psychosocial adjustment difficulties were high; 65.6% showed clinical maladjustment   3. Strong negative correlation between maladjustment and both physical and mental HRQoL     **Significant predictors of poor HRQoL:**   - - Female gender   - Unemployment   - Poor income   - Lower education   - More frequent relapses     **Implication:**   - - Psychosocial adjustment is a critical determinant of QoL in MS   - Need for targeted psychiatric/psychosocial interventions in MS care   - Suggests shifting some MS care to primary care/mental health services | N/A | N/A |
| I. Milivojevic et. Al[74] | **Setting: Mixed:**   - Inpatient rehabilitation (28.5% of patients) - Outpatient/ambulatory rehabilitation (17.4%) - Home-based therapy (4.7%)   **Duration of rehab (mean):**   - Inpatient: 20.6 days (range: 14–21) - Outpatient: 13.6 days (range: 4–28) - Home-based: 14.3 days (range: 7–21)   **Frequency:**  Not explicitly described, but inferred as daily in inpatient settings  Ambulatory and home settings likely less frequent (details not reported) | Inpatient and outpatient rehab were delivered in hospital or clinic settings    Most referrals were made by neurologists (inpatient: 77.8%; outpatient: 18.2%) or PMR specialists (physiatrists) | Utilization of physical rehabilitation services (inpatient, outpatient, home-based) | - 1. Level of disability via Expanded Disability Status Scale (EDSS)   2. Self-reported benefit from rehabilitation on a 1–5 scale   3. Correlation between rehab use and EDSS analyzed using t-tests     Results:   - 1. 41.3% of patients received some form of rehabilitation in the last 2 years   2. Inpatient rehab was most common (28.5%)   3. Patients who received any form of rehab had higher EDSS scores (more severe impairment)      - Inpatient: EDSS 2.9      - Outpatient: EDSS 3.0      - No rehab: EDSS 1.0   4. Patient satisfaction scores:      - Inpatient = 3.4/5      - Outpatient = 3.1/5      - Home = 3.3/5   5. Rehabilitation is underutilized in early stages, despite evidence that early rehab may offer best outcomes   6. Authors recommend greater awareness and integration of rehab in MS care planning | - 1. EDSS (clinician-assessed)   2. Self-evaluation scale (1 = no improvement; 5 = significant improvement)   3. Questionnaires capturing service use history | Retrospective 2 year analysis (2010-2011) | - 1. 41.3% of patients received some form of rehabilitation in the last 2 years   2. Inpatient rehab was most common (28.5%)   3. Patients who received any form of rehab had higher EDSS scores (more severe impairment)      - Inpatient: EDSS 2.9      - Outpatient: EDSS 3.0      - No rehab: EDSS 1.0   4. Patient satisfaction scores:      - Inpatient = 3.4/5      - Outpatient = 3.1/5      - Home = 3.3/5   5. Rehabilitation is underutilized in early stages, despite evidence that early rehab may offer best outcomes   6. Authors recommend greater awareness and integration of rehab in MS care planning | N/A | N/A |

Table S4 : Risk of bias table for experimental studies

| Article | Q1 | Q2 | Q3 | Q4 | Q5 | Q6 | Q7 | Q8 | Q9 | Q10 | Q11 | Q12 | Q13 | Risk level |
| --- | --- | --- | --- | --- | --- | --- | --- | --- | --- | --- | --- | --- | --- | --- |
| STROKE |  |  |  |  |  |  |  |  |  |  |  |  |  |  |
| Mattioli et al., 2014 [20] | Yes | Unclear | Yes | No | No | Yes | Yes | Yes | Yes | Yes | Unclear | Yes | Yes | Low risk |
| Lin et al., 2019 [22] | Yes | Unclear | Yes | No | No | Yes | Unclear | Yes | Yes | No | Unclear | Yes | Yes | Low risk |
| Liu-Ambrose et al., 2015 [23] | Yes | Unclear | Yes | No | No | Yes | Yes | Yes | Yes | No | Unclear | Yes | Yes | Low risk |
| Lindley et al., 2017 [27] | Yes | Yes | Yes | No | No | Yes | Yes | Yes | Yes | Yes | Yes | Yes | Yes | Low risk |
| Heron et al., 2017 [44] | Yes | Yes | Yes | No | No | Yes | No | Yes | Yes | Yes | Yes | Yes | Yes | Low risk |
| Ntsiea et al., 2014 [46] | Yes | Yes | Yes | No | No | Yes | Yes | Yes | Yes | Yes | Yes | Yes | Yes | Low risk |
| DEMENTIA |  |  |  |  |  |  |  |  |  |  |  |  |  |  |
| Quinn et al., 2016 [52] | Yes | Yes | Yes | No | No | Yes | Yes | Yes | Yes | Yes | Yes | Yes | Yes | Low risk |
| Sakamoto et al., 2013 [55] | Yes | Unclear | Yes | No | No | Yes | Yes | Yes | Yes | Yes | Unclear | Yes | Yes | Low risk |
| Charlesworth et al., 2016 [61] | Yes | Unclear | Yes | No | No | Yes | Yes | Yes | Yes | Yes | Yes | Yes | Yes | Low risk |
| MS |  |  |  |  |  |  |  |  |  |  |  |  |  |  |
| Plow et al., 2014 [68] | Yes | Unclear | Yes | No | No | Yes | Unclear | Yes | Yes | No | No | Yes | Yes | Low risk |

Table S5 : Risk of bias table for observational studies

| Article | Q1 | Q2 | Q3 | Q4 | Q5 | Q6 | Q7 | Q8 | Q9 | Q10 | Q11 | Q12 | Q13 | Risk level |
| --- | --- | --- | --- | --- | --- | --- | --- | --- | --- | --- | --- | --- | --- | --- |
| PARKINSON |  |  |  |  |  |  |  |  |  |  |  |  |  |  |
| Cholewa et al., 2016 [11] | No | No | Unclear | Unclear | Unclear | No | No | Unclear | No | N/A | Yes | Yes | Unclear | High risk |
| Swink et al., 2020 [12] | No | No | N/A | Unclear | Unclear | No | No | Unclear | No | N/A | N/A | N/A | Unclear | Moderate risk |
| Spinal cord injury |  |  |  |  |  |  |  |  |  |  |  |  |  |  |
| Kern et al., 2019 [13] | N/A | N/A | N/A | Unclear | Unclear | No | N/A | Unclear | No | N/A | N/A | N/A | No | Low risk |
| STROKE |  |  |  |  |  |  |  |  |  |  |  |  |  |  |
| Gallacher et al., 2018 [14] | N/A | N/A | N/A | Unclear | Unclear | No | N/A | Unclear | No | N/A | N/A | N/A | No | Low risk |
| Cobley et al., 2013 [15] | N/A | No | Unclear | Unclear | Unclear | No | N/A | Unclear | No | N/A | N/A | N/A | No | Moderate risk |
| Glickman et al., 2018 [16] | No | No | Unclear | Unclear | Unclear | No | N/A | Unclear | No | N/A | Yes | Yes | Unclear | High risk |
| Ashaie et al., 2022 [17] | No | No | N/A | Unclear | N/A | No | No | Yes | No | N/A | N/A | Yes | Unclear | Low risk |
| Sinclair et al., 2014 [18] | N/A | N/A | N/A | Unclear | Unclear | No | N/A | Unclear | No | N/A | N/A | N/A | No | Low risk |
| Askew et al., 2020 [19] | No | No | N/A | Unclear | N/A | No | No | Yes | No | N/A | N/A | No | Unclear | Moderate risk |
| Martin et al., 2023 [21] | N/A | N/A | N/A | Unclear | Unclear | No | N/A | Unclear | No | N/A | N/A | N/A | No | Low risk |
| O’Callaghan et al., 2024 [24] | N/A | N/A | N/A | Unclear | Unclear | No | N/A | Unclear | No | N/A | N/A | N/A | No | Low risk |
| May et al., 2023 [25] | N/A | N/A | N/A | Unclear | Unclear | No | N/A | Unclear | No | N/A | N/A | N/A | No | Low risk |
| Koositamongkol et al., 2013 [26] | No | No | N/A | Unclear | Unclear | No | N/A | Yes | No | N/A | N/A | Yes | Unclear | Moderate risk |
| Tramonti et al., 2014 [28] | N/A | N/A | N/A | Unclear | Unclear | No | No | Unclear | No | N/A | N/A | N/A | No | Low risk |
| Fama et al., 2016 [29] | No | No | N/A | Unclear | Unclear | No | No | Unclear | No | N/A | N/A | N/A | No | Moderate risk |
| Manning et al., 2024 [30] | N/A | N/A | N/A | Unclear | N/A | Yes | N/A | Yes | No | N/A | N/A | N/A | Unclear | Low risk |
| Balasooriya-Smeekens et al., 2020 [31] | N/A | N/A | N/A | Unclear | Unclear | No | N/A | Unclear | No | N/A | N/A | N/A | No | Low risk |
| Moore et al., 2024 [32] | N/A | N/A | N/A | Unclear | Unclear | No | N/A | Unclear | No | N/A | N/A | N/A | No | Low risk |
| Bērziņa et al., 2016 [33] | No | No | N/A | Unclear | N/A | No | N/A | Yes | No | N/A | N/A | No | Unclear | Moderate risk |
| White et al., 2016 [34] | No | No | N/A | Unclear | Unclear | No | Yes | Yes | No | N/A | N/A | No | No | High risk |
| Delhey et al., 2024 [35] | No | No | N/A | Unclear | N/A | No | Yes | Yes | No | N/A | N/A | No | Unclear | Moderate risk |
| Ng et al., 2013 [36] | No | Unclear | Yes | Unclear | No | Unclear | N/A | Yes | No | N/A | Yes | Yes | Unclear | Moderate risk |
| Egan et al., 2014 [37] | No | No | N/A | Unclear | Unclear | No | Yes | No | No | N/A | N/A | No | Unclear | Moderate risk |
| Tielemans et al., 2016 [38] | N/A | N/A | N/A | Unclear | Unclear | Unclear | N/A | Yes | No | N/A | N/A | N/A | Unclear | Low risk |
| Matos et al., 2024 [39] | No | No | Unclear | Unclear | N/A | No | N/A | Unclear | No | N/A | Yes | Yes | Unclear | Moderate risk |
| Umemura et al., 2023 [40] | No | No | Yes | Unclear | N/A | Yes | Yes | Yes | No | N/A | Yes | Yes | Unclear | High risk |
| Langhammer et al., 2018 [41] | No | No | N/A | Unclear | Unclear | No | Yes | Yes | No | N/A | N/A | Yes | Unclear | Moderate risk |
| Westerlind et al., 2020 [42] | No | No | Yes | Unclear | N/A | No | N/A | Yes | No | N/A | Yes | Yes | Unclear | Moderate risk |
| Lehnerer et al., 2019 [40] | No | No | N/A | Unclear | Unclear | Yes | N/A | Yes | No | N/A | N/A | N/A | Unclear | Moderate risk |
| Wassenius et al., 2023 [45] | N/A | N/A | N/A | Unclear | Unclear | No | N/A | Unclear | No | N/A | N/A | N/A | Unclear | Low risk |
| Moon KT et al., 2022 [47] | N/A | N/A | N/A | Unclear | Unclear | No | Yes | Yes | No | N/A | N/A | N/A | Unclear | Low risk |
| Tseng, 2024 [48] | No | No | N/A | Unclear | Unclear | No | No | Unclear | No | N/A | N/A | No | No | Moderate risk |
| Clarke et al., 2023 [49] | N/A | N/A | N/A | Unclear | Unclear | Yes | N/A | Yes | No | N/A | N/A | N/A | Unclear | Low risk |
| Powers et al., 2023 [50] | No | No | N/A | Unclear | Unclear | Yes | N/A | Yes | No | N/A | N/A | Yes | Unclear | Moderate risk |
| Rosbergen et al., 2017 [51] | N/A | N/A | N/A | Unclear | Unclear | No | N/A | Unclear | No | N/A | N/A | N/A | Unclear | Low risk |
| DEMENTIA |  |  |  |  |  |  |  |  |  |  |  |  |  |  |
| Pigmouguet et al., 2016 [53] | No | No | N/A | Unclear | Unclear | No | Yes | Yes | No | N/A | N/A | Yes | Unclear | Moderate risk |
| van Haefen-van Dijk et al., 2015 [54] | No | No | Yes | Yes | Yes | No | No | Yes | No | Unclear | Yes | Yes | Yes | Moderate risk |
| Szczesniak et al., 2018 [56] | N/A | N/A | N/A | Unclear | N/A | Unclear | N/A | Unclear | No | N/A | N/A | N/A | Unclear | Low risk |
| Omote et al., 2023 [57] | N/A | N/A | N/A | Unclear | Unclear | No | N/A | Unclear | No | N/A | N/A | N/A | Unclear | Low risk |
| van Rijn et al., 2019 [58] | N/A | N/A | N/A | Unclear | Unclear | No | N/A | Unclear | No | N/A | N/A | N/A | Unclear | Low risk |
| Oesterholm et al., 2024 [59] | N/A | N/A | N/A | Unclear | Unclear | No | N/A | Unclear | No | N/A | N/A | N/A | No | Low risk |
| Chew et al., 2015 [60] | N/A | N/A | N/A | Unclear | Unclear | No | No | Yes | No | N/A | N/A | N/A | Unclear | Low risk |
| Tam-Tham et al., 2016 [62] | N/A | N/A | N/A | Unclear | Unclear | Unclear | N/A | Unclear | No | N/A | N/A | N/A | Unclear | Low risk |
| Wattmo et al., 2014 [63] | No | No | Unclear | Unclear | Unclear | No | No | Yes | No | N/A | Yes | Yes | Unclear | High risk |
| Droes et al., 2019 [64] | No | Yes | Yes | Unclear | Unclear | No | Unclear | Yes | No | N/A | Yes | Yes | Unclear | Moderate risk |
| Van ’t Leven et al., 2018 [65] | N/A | N/A | N/A | Unclear | Unclear | No | N/A | Unclear | No | N/A | N/A | N/A | No | Low risk |
| Sangi et al., 2020 [66] | No | Unclear | Yes | Unclear | Unclear | No | No | Yes | No | N/A | Yes | Yes | Unclear | Moderate risk |
| Rosvik et al., 2021 [67] | N/A | N/A | N/A | Unclear | Unclear | Unclear | N/A | Unclear | No | N/A | N/A | N/A | No | Low risk |
| MULTIPLE SCLEROSIS |  |  |  |  |  |  |  |  |  |  |  |  |  |  |
| Glattacker et al., 2018 [69] | No | Yes | N/A | Unclear | N/A | Yes | N/A | Yes | No | N/A | N/A | Yes | Unclear | Low risk |
| Russell et al., 2023 [70] | N/A | N/A | N/A | Unclear | Unclear | No | N/A | Unclear | No | N/A | N/A | N/A | No | Low risk |
| Lahelle et al., 2020 [71] | N/A | N/A | N/A | Unclear | Unclear | No | N/A | Unclear | No | N/A | N/A | N/A | No | Low risk |
| Månsson Lexell et al., 2014 [72] | N/A | N/A | N/A | Unclear | Unclear | No | Unclear | Yes | No | N/A | N/A | N/A | Unclear | Low risk |
| Hyarat et al., 2019 [73] | No | No | N/A | Unclear | N/A | No | N/A | Unclear | No | N/A | N/A | Unclear | Unclear | Moderate risk |
| Milivojevic et al., 2013 [74] | No | Unclear | N/A | Unclear | Unclear | No | N/A | Yes | No | N/A | N/A | Yes | Unclear | Moderate risk |
